# Functional MRI reveals brain-wide actions of thalamically-initiated oscillatory activities on associative memory consolidation

**DOI:** 10.1038/s41467-023-37682-8

**Published:** 2023-04-17

**Authors:** Xunda Wang, Alex T. L. Leong, Shawn Z. K. Tan, Eddie C. Wong, Yilong Liu, Lee-Wei Lim, Ed X. Wu

**Affiliations:** 1 Laboratory of Biomedical Imaging and Signal Processing, The University of Hong Kong, Pokfulam Hong Kong SAR, China; 2 Department of Electrical and Electronic Engineering, The University of Hong Kong, Pokfulam Hong Kong SAR, China; 3 School of Biomedical Sciences, Li Ka Shing Faculty of Medicine, The University of Hong Kong, Pokfulam Hong Kong SAR, China

**Keywords:** Functional magnetic resonance imaging, Optogenetics, Neural circuits, Consolidation

## Abstract

As a key oscillatory activity in the brain, thalamic spindle activities are long believed to support memory consolidation. However, their propagation characteristics and causal actions at systems level remain unclear. Using functional MRI (fMRI) and electrophysiology recordings in male rats, we found that optogenetically-evoked somatosensory thalamic spindle-like activities targeted numerous sensorimotor (cortex, thalamus, brainstem and basal ganglia) and non-sensorimotor limbic regions (cortex, amygdala, and hippocampus) in a stimulation frequency- and length-dependent manner. Thalamic stimulation at slow spindle frequency (8 Hz) and long spindle length (3 s) evoked the most robust brain-wide cross-modal activities. Behaviorally, evoking these global cross-modal activities during memory consolidation improved visual-somatosensory associative memory performance. More importantly, parallel visual fMRI experiments uncovered response potentiation in brain-wide sensorimotor and limbic integrative regions, especially superior colliculus, periaqueductal gray, and insular, retrosplenial and frontal cortices. Our study directly reveals that thalamic spindle activities propagate in a spatiotemporally specific manner and that they consolidate associative memory by strengthening multi-target memory representation.

## Abstract (web summary)

Thalamic spindle activities may support memory consolidation. Here the authors show that optogenetically-evoked somatosensory thalamic spindle-like activity enhances memory performance in male rats.

## Introduction

Large-scale spatiotemporal coordination of brain neural activities is vital for effective information processing in the brain^1,2^. As major oscillatory activities, spindle activities are thought to orchestrate neuronal populations to support critical functions, such as memory consolidation, sensory gating, and sleep/arousal regulation^3–5^. Spindle activities are prominent in NREM sleep and occur occasionally in drowsy and awake quiescent states^6–9^. Spindle activities refer to 7–15 Hz, 0.5–3 s and 4–25 cycles brief oscillatory events that exhibit distinct spindle-shaped signal envelopes in electroencephalogram (EEG) or local field potential (LFP) waveforms^4,10,11^. Spindle activities arise from local thalamo-cortical circuits, which have been long postulated to subsequently engage remote targets and establish a spatiotemporal organization for long-range inter-regional interactions to subserve memory consolidation processes^4,12^. Thus, it is imperative to interrogate the spatiotemporal propagation properties and targeting characteristics of spindle activities and their large-scale actions for fundamental understanding of how spindle activities support memory consolidation at the systems level^13–16^.

To tackle this question, previous studies have mostly examined the occurrence of scalp EEG-measured spindle activities in humans and animals^14,15,17^ but with very limited brain region specificity. Using simultaneous EEG and blood-oxygenation-level–dependent (BOLD) functional MRI (fMRI), human studies have reported the correlation of fMRI BOLD activations at several brain regions, such as thalamus and sensorimotor cortices, with EEG-measured spindle activities by searching for the temporal coherence between EEG and BOLD signals^13,18,19^. These findings suggest the large-scale targeting capability of thalamo-cortical spindle activities. Several studies postulate that spindle temporal characteristics, which are key markers of normal spindle functions^3,4,14,15^, may influence the local or long-range spatial targeting of thalamo-cortical spindle activities^3,4,13–15^. However, the propagation properties of spindle activities and their downstream brain-wide targets remain elusive. This knowledge gap arose primarily from the nature of passive observations in existing spindle mapping approaches, including the EEG activity-triggered fMRI analysis. For example, spindle activities recorded at a specific brain region reflect the culmination of multiple spindle initiations and propagation processes mediated by different thalamic nuclei, their corresponding thalamo-cortical circuits and networks^4,7,20^.

Hence, it is fundamentally difficult to determine how spindle activities observed at different regions are precisely related to single or multiple specific thalamic initiations or sites. Further, such indirect approaches are highly dependent upon recording techniques and locations. So far, EEG activity-triggered fMRI studies^13,18,19^ neither detected any spindle activities at multiple key subcortical (e.g., sensorimotor brainstem and amygdala)^21–24^ and deep cortical (e.g., entorhinal cortex) regions^21,23,25,26^, nor captured the distinction between widely distributed slow spindle activities vs. localized fast spindle activities shown in EEG/invasive recording studies^14,15,21^. Therefore, the existing EEG/fMRI approaches can help to identify some action sites of spindle activities, but they are intrinsically insensitive and inadequate for elucidating how spindle activities initiated at a specific thalamic nucleus propagate and target various downstream structures brain-wide. To overcome this barrier, we must integrate whole-brain activity mapping with direct, spatiotemporally-precise initiation of spindle activities.

Functionally, oscillatory spindle activities have long been proposed to mediate memory consolidation by orchestrating neuronal activities spatially and temporally over distributed neural circuits^5,27–29^. Invasive recording studies have demonstrated an association among local spindle activity feature changes, memory representation reactivation, and memory performance^30–32^. Meanwhile, EEG activity-triggered fMRI studies suggested that some regional (sensorimotor cortical or basal ganglia) BOLD activities correlated with post-learning spindle activities, partially resembled the local memory representation acquired during learning, and predicted memory performance^19,33,34^. These studies have advanced our understanding of the local circuitry aspect of spindle-associated memory consolidation processes, such as sensorimotor modality-specific information reactivation in local sensorimotor cortices, monosynaptic or short-range circuits for locally strengthening memory representation. Further, decreases in spindle activities have been found to be one of the hallmarks of aging-related memory consolidation deficits^14,16,35^. Studies also implicated potential therapeutic interventions to rescue such aging-related memory deficits through targeted potentiation of regional spindle activities^36,37^. Despite such progress, we have yet to delineate the brain-wide propagating spindle activities and their actions on memory consolidation at systems level. It is imperative to examine whether and how propagating spindle activities influence memory representation in a relatively local or highly distributed manner and act on the inter-regional integration or network connectivity. At present, this endeavor is hampered by our lack of experimental paradigm for precise spindle activity initiation, accurate visualization of downstream targets, and direct mapping of functional consequences within the entire brain.

Here, we interrogated the spatiotemporal targeting characteristics of thalamo-cortical spindle activities and their brain-wide actions on associative memory consolidation. Using whole-brain fMRI, optogenetic stimulation, and multisite electrophysiology recordings, we demonstrated the stimulation frequency- and length-dependent brain-wide cross-modal targeting of somatosensory thalamically-evoked spindle-like activities. Using parallel behavioral and visual fMRI experiments, we directly revealed that these evoked spindle-like activities supported visual-somatosensory associative fear memory consolidation by potentiating neural memory representation in key sensorimotor and limbic integrative regions brain-wide, especially superior colliculus, periaqueductal gray, insular, retrosplenial and frontal cortices.

## Results

We first performed whole-brain fMRI on optogenetically transfected animals^38–40^ to investigate the brain-wide propagation and targeting characteristics of optogenetically-evoked somatosensory thalamic activities at or beyond spindle frequencies and lengths. Guided by the fMRI results, we conducted complementary multisite extracellular electrophysiology recordings in a separate group of animals to examine neural activities underlying the varied optogenetically-evoked brain-wide BOLD activation patterns. Subsequently, we tested the effects of evoking brain-wide activities at slow spindle frequency (8 Hz) and long spindle length (3 s) during the memory consolidation phase on memory performance of three animal groups (i.e., Optogenetic/OG, Sham, and Naive) in fear conditioning experiments. In parallel, visual fMRI experiments were conducted in a separate group of OG and Sham animals that underwent fear conditioning to investigate the large-scale actions of the evoked activities on memory consolidation.

### Brain-wide and cross-modal targeting by somatosensory thalamic activities optogenetically-evoked at spindle frequencies and lengths

**Fig. 1:**
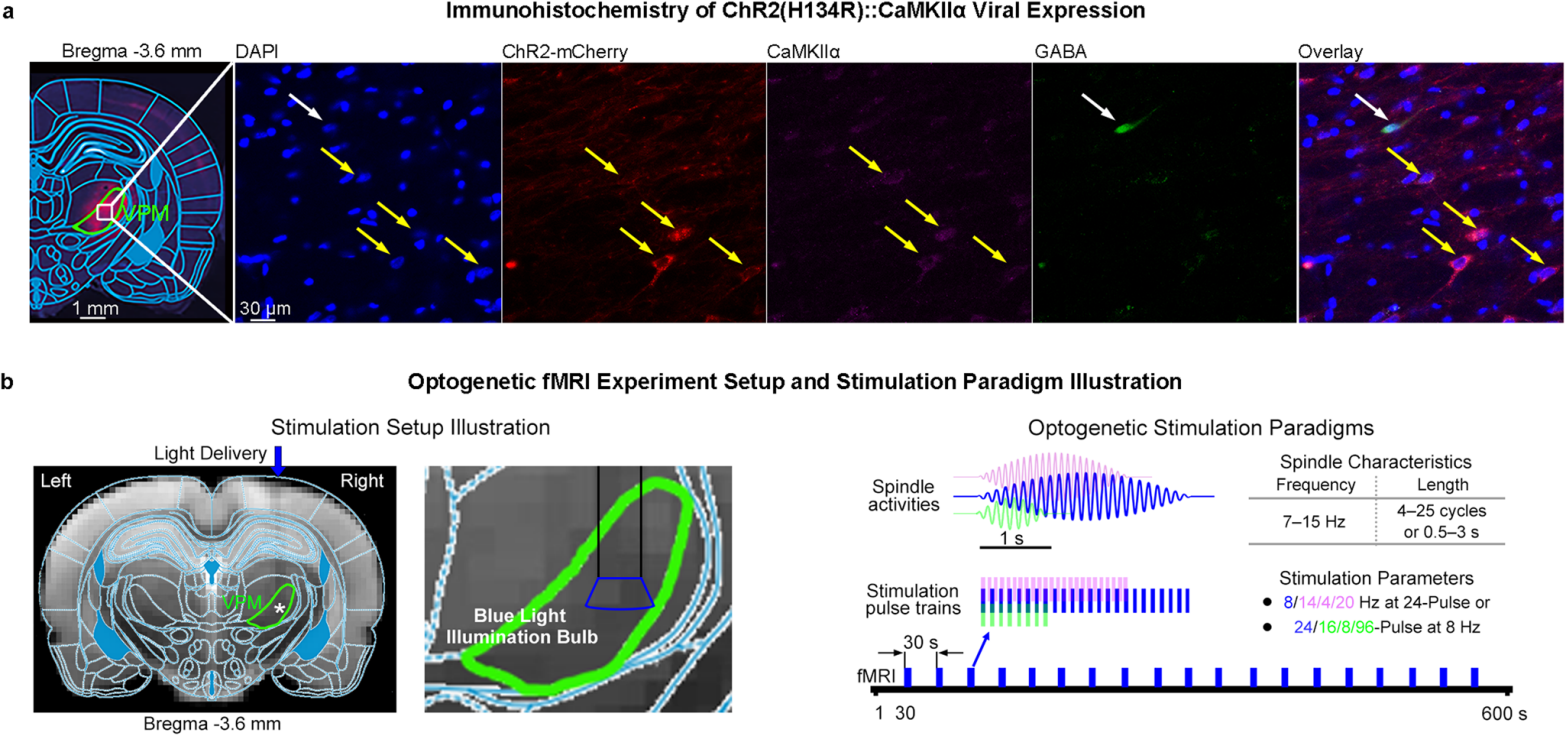
Histological characterization of ChR2::CaMKIIα viral expression in ventral posteromedial (VPM) thalamocortical excitatory neurons and optogenetic fMRI experiment setup and stimulation paradigms. **a** Confocal images of ChR2-mCherry expression in VPM with lower (Left) and higher (Right) magnification. Overlay of images co-stained for the nuclear marker DAPI, excitatory marker CaMKIIα, inhibitory marker GABA, and ChR2-mCherry revealed colocalization of ChR2-mCherry and CaMKIIα in the cell body of thalamocortical excitatory neurons (indicated by yellow arrows), not GABAergic inhibitory neurons (indicated by white arrows). **b** T2-weighted anatomical MRI image shows the location of the implanted optical fiber (asterisk, stimulation site) and illumination bulb at 10% of maximum light intensity (350 um diameter) (see *Methods* for details) overlaid at the tip of the optical fiber shows that the delivered blue light only illuminated VPM without spreading to neighboring thalamic nuclei (Left), and the illustration of a typical optogenetic fMRI experiment with different stimulation paradigms designed to evoke activities within or beyond the typical ranges of spindle frequency and length (Right). Stimulation pulse train and spindle activity at 8 Hz 24 pulses/cycles are illustrated in blue, while examples of stimulation and spindle activity at 14 Hz 24 pulses/cycles or 8 Hz 8 pulses/cycles are in purple and green, respectively.

We devised optogenetic stimulations in CaMKIIα expressing thalamocortical excitatory neurons in the somatosensory-specific ventral posteromedial thalamic nucleus (VPM) of lightly anesthetized (1.0% isoflurane) normal adult male rats (Fig. 1). Histological characterization confirmed specific ChR2-mCherry viral expression in VPM thalamocortical excitatory neurons, not GABAergic inhibitory neurons, (Fig. 1a) and in VPM projection terminals at somatosensory cortices (Fig. S1). Brief blue light pulse trains (10 ms pulse width, 40 mW/mm^2^ light intensity) with differing frequencies (8, 14, 4 and 20 Hz 24-pulse) or lengths (8-, 16-, 24-, and 96-pulse at 8 Hz, corresponding to 8, 16, 24 and 96 cycles or 1, 2, 3, and 12 s, respectively) were delivered once every 30 s (Fig. 1b). The stimulation frequencies and lengths were chosen within or beyond the range of spindle frequencies and lengths (i.e., within: 8 and 14 Hz 24-pulse, and 8 Hz 8- and 16- pulse; beyond: 4 and 20 Hz 24-pulse, and 8 Hz 96-pulse)^4,10^. We performed whole-brain fMRI to determine the brain-wide activation patterns driven by these somatosensory thalamic stimulations.

**Fig. 2:**
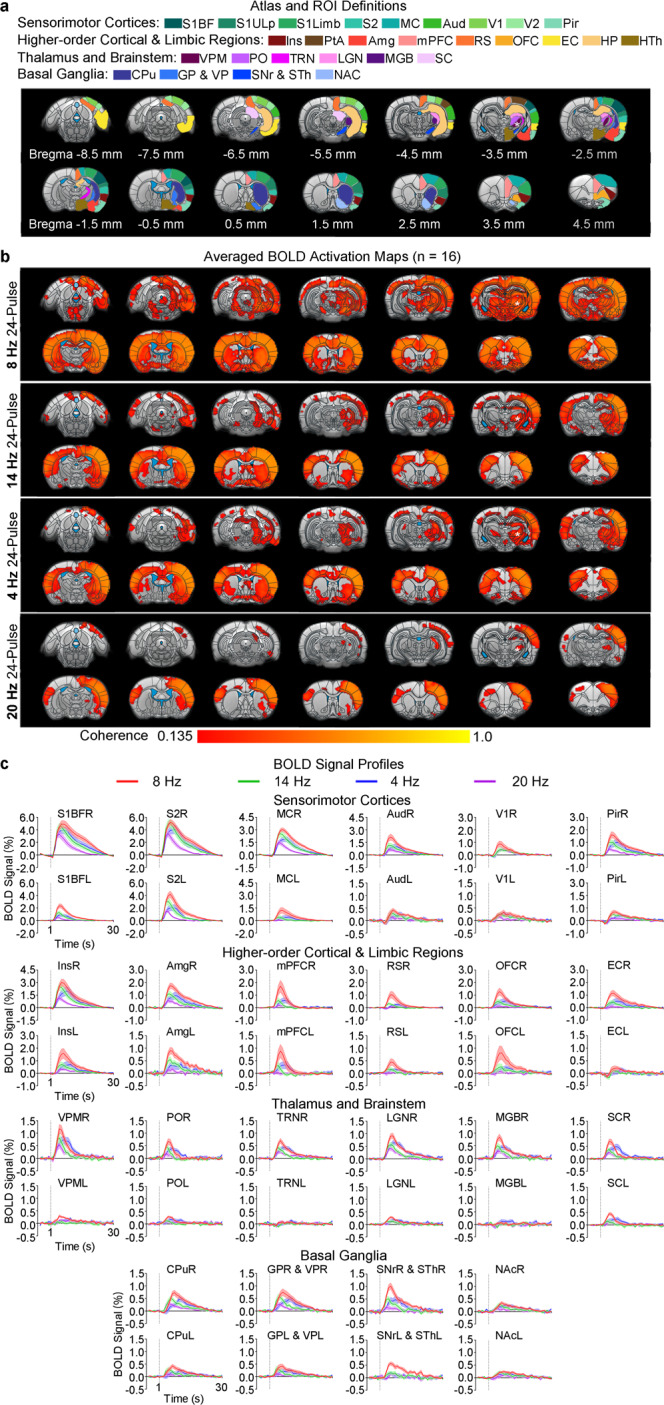
BOLD activations from 24-pulse stimulations across typical spindle frequencies reveal brain-wide cross-modal targets and their respective frequency-dependent response property. **a** Illustration of atlas-based ROI definitions in the sensorimotor cortices, higher-order cortical and limbic regions, thalamus and brainstem, and basal ganglia (asterisk, stimulation site). Sensorimotor cortices: primary somatosensory-barrel field (S1BF), limb region (S1Limb), and upper lip region (S1ULp), secondary somatosensory cortex (S2), motor cortices (MC), auditory cortex (Aud), primary and secondary visual cortex (V1 and V2), piriform cortex (Pir); higher-order cortical and limbic regions: insular area (Ins), parietal associate cortex (PtA), amygdala (Amg), medial prefrontal cortex (mPFC), retrosplenial cortex (RS), orbital frontal cortex (OFC), entorhinal and parahippocampal area (EC), hippocampus (HP), and hypothalamus (HTh); thalamus and brainstem: ventral posteromedial thalamus (VPM), posterior nuclei (PO), thalamic reticular nucleus (TRN), visual thalamus (LGN), auditory thalamus (MGB), superior colliculus (SC); basal ganglia: caudate putamen (CPu), globus pallidus and ventral pallidum (GP & VP), substantia nigra and subthalamus (SNr & STh), nucleus accumbens (NAc). **b** Averaged BOLD activation maps for 24-pulse optogenetic stimulations at different frequencies: 8, 14, 4, 20 Hz (*n* = 16; asterisk, stimulation site; two-tailed coherence tests, coherence of 0.135 corresponds to *P* < 0.001, followed by two-tailed one-sample group level *t*-tests, threshold-free cluster enhancement with family wise error rate, TFCE-FWE, corrected *P* < 0.05; see Bonferroni-corrected *P* < 0.05 in Fig. S3). Robust positive BOLD activations were observed in numerous sensorimotor-related cortical, thalamic, brainstem and basal ganglia regions, and non-sensorimotor limbic regions upon the 8 Hz 24-pulse stimulation (slow spindle frequency). Such brain-wide activations weakened and focalized with stimulations at 14/4 Hz (fast/below spindle frequency) and were further restricted to mainly sensorimotor regions at 20 Hz (above spindle frequency). See statistical comparisons in Fig. S4. **c** BOLD signal profiles extracted from atlas-based ROIs defined in (**a**) (error bar indicates ± s.e.m.). Source data are provided as a Source Data file.

Robust brain-wide BOLD fMRI activations were detected across numerous sensorimotor and non-sensorimotor regions during the 8 Hz 24-pulse, slow spindle frequency and long spindle length^4^, stimulation of VPM (Fig. 2 and Figs. S2, S3). The primary and higher-order sensorimotor-related regions that were activated included sensorimotor cortices, such as bilateral somatosensory (S1BF, S1Limb, S1ULp & S2), motor (MC), auditory (Aud), visual (V1 & V2), and piriform (Pir) cortices, insular area (Ins), parietal association cortex (PtA); lateral and posterior sensory thalamus, such as bilateral VPM, posterior medial nucleus (PO), ipsilateral thalamic reticular nucleus (TRN), bilateral lateral geniculate nucleus (LGN), and ipsilateral medial geniculate nucleus (MGB); and sensorimotor brainstem, such as superior colliculus (SC). We also observed activations at the motor control-related basal ganglia regions, including bilateral caudate putamen (CPu), pallidum (consists of globus pallidus & ventral pallidum, GP & VP), nucleus accumbens (NAc), and subthalamic nucleus and substantia nigra (STh & SNr). The notable non-sensorimotor regions that were activated included cortical and subcortical regions in the limbic system, such as bilateral amygdala (Amg), medial prefrontal cortex (mPFC), retrosplenial cortex (RS), orbital frontal cortex (OFC), entorhinal and parahippocampal area (EC), hippocampus (HP), and hypothalamus (HTh).

To examine how stimulation frequency influences brain-wide cross-modal BOLD activations, we varied the stimulation frequency (from 8 to 14, 4, and 20 Hz) while maintaining the length at 24-pulse (i.e., 24 cycles) (Fig. 2 and Figs. S2, S3). Stimulation at fast spindle frequency—14 Hz (4) decreased the strength of brain-wide BOLD activations compared to those at the 8 Hz stimulation (slow spindle frequency), especially at remote regions beyond the somatosensory thalamo-cortical circuit (i.e., bilateral Aud, V1 & V2, Pir, PtA, SC, CPu, GP & VP, NAc, STh & SNr, Amg, Ins, mPFC, OFC, RS, EC, HP, and HTh). Further, the spatial extent of BOLD activations at these remote regions became more focal, especially at bilateral OFC and RS, contralateral Amg and Pir, and the anterior parts of bilateral mPFC and CPu. Stimulation at 4 Hz, below the slow spindle frequency, also evoked weaker and more focal brain-wide BOLD activations than those evoked by 8 Hz stimulation, resulting in a similar brain-wide activation pattern as in the 14 Hz stimulation. However, the brain-wide BOLD activations evoked by stimulation at 20 Hz (i.e., above spindle frequency) were largely restricted to sensorimotor regions (i.e., bilateral somatosensory and motor cortices, ipsilateral visual and auditory cortices, thalamus, brainstem and basal ganglia) with the exception of small and weak activation clusters at ipsilateral Amg, RS and mPFC. Together with the local ipsilateral VPM and somatosensory cortical BOLD activations, polysynaptic activations in remote regions (e.g., contralateral sensorimotor cortices or bilateral limbic regions) were significantly affected by the stimulation frequency (Fig. S4).

**Fig. 3:**
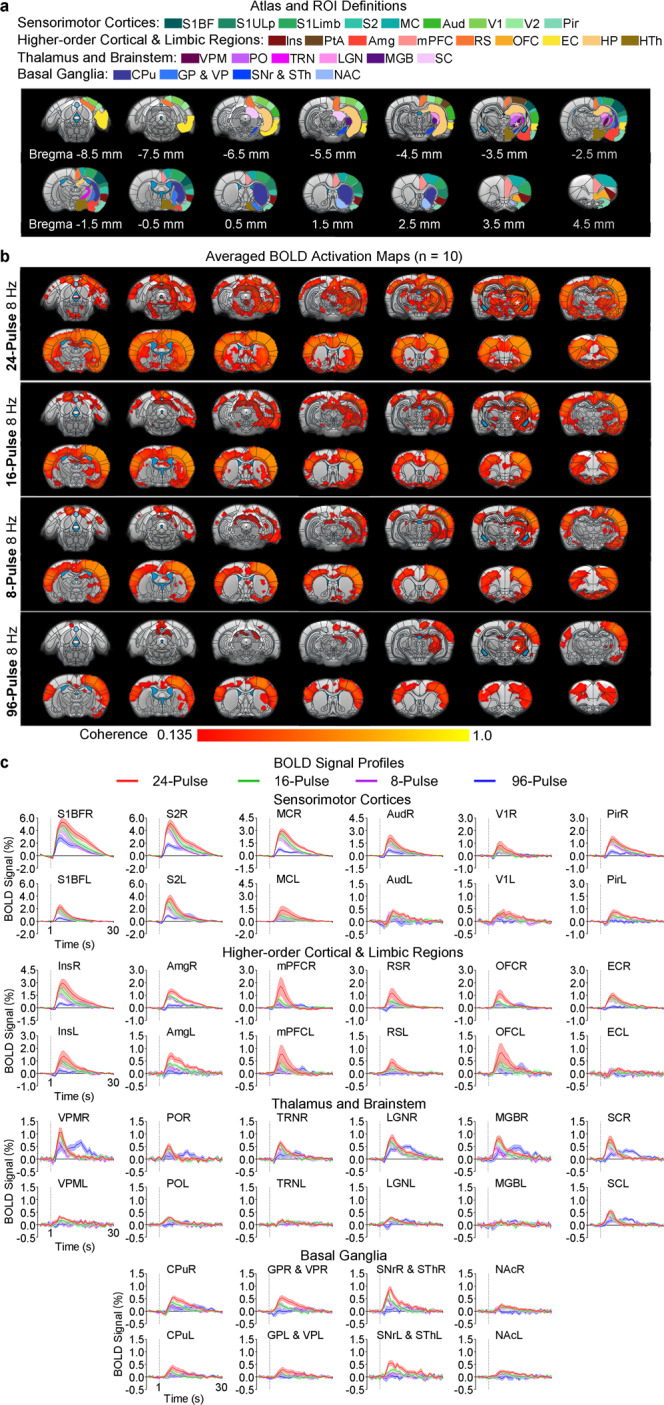
Length-dependent response properties for brain-wide cross-modal targets of somatosensory thalamically-evoked activities upon 8 Hz stimulation. **a** Illustration of atlas-based ROI definitions in the sensorimotor cortices, higher-order cortical and limbic regions, thalamus and brainstem, and basal ganglia (asterisk, stimulation site). **b** Averaged BOLD activation maps for 8 Hz optogenetic stimulations at different lengths: 8-, 16-, 24-, and 96-pulse (*n* = 10; asterisk, stimulation site; two-tailed coherence tests, coherence of 0.135 corresponds to *P* < 0.001, followed by two-tailed one-sample group level *t*-tests, TFCE-FWE corrected *P* < 0.05; see Bonferroni-corrected *P* < 0.05 in Fig. S6). Similar robust brain-wide cross-modal BOLD activations upon the 8 Hz 24-pulse stimulation were observed as in Fig. 2. Such brain-wide activations weakened and focalized with stimulations at 16-/8-pulse (reduced spindle length) and were further restricted to mainly sensorimotor regions at the 96-pulse (excessive length) stimulation. See statistical comparisons in Fig. S7. **c** BOLD signal profiles extracted from atlas-based ROIs defined in (**a**) (error bar indicates ± s.e.m.). Source data are provided as a Source Data file.

To investigate how the length of stimulation pulse trains influences brain-wide BOLD activation patterns, we varied the length (8-, 16-, 24-, or 96-pulse, corresponding to 8, 16, 24 and 96 cycles or 1, 2, 3, and 12 s, respectively) during a constant frequency at 8 Hz. Note that 8 Hz stimulation evoked the most robust activations (Fig. 3 and Figs. S5, S6). We observed the strongest and most widespread BOLD activations using the 24-pulse stimulation, which is the longest within the range of typically recorded spindle length (0.5–3 s & 4–25 cycles). 16- and 8-pulse stimulations (i.e., 2 s and 1 s or 16 and 8 cycles respectively) evoked weaker BOLD activations than the 24-pulse stimulation at deep and/or remote regions such as Amg, Ins, frontal and posterior cortical regions, hippocampus, and basal ganglia. We also found decreased spatial extent for BOLD activations at contralateral Amg and Pir, bilateral OFC, and the anterior part of bilateral mPFC and CPu. Notably, the BOLD activations upon the 96-pulse stimulation (i.e., excessive length beyond the typical range) were restricted mainly to sensorimotor regions (i.e., bilateral somatosensory and motor cortices, brainstem, ipsilateral visual and auditory cortices, thalamus, and basal ganglia), except for small clusters of weak ipsilateral Amg, RS and bilateral mPFC activations. Like stimulation frequency, stimulation length had significant influence on polysynaptic activations in remote regions beyond local ipsilateral VPM and somatosensory cortical activations (Fig. S7). These results suggest that variations in the brain-wide BOLD activation patterns due to different stimulation frequencies and lengths are driven by underlying neural activities and their subsequent interactions at systems level. Note that amplitude of all BOLD responses was stable, and we did not observe any baseline drifts in the BOLD signals over the stimulation periods across all stimulation pulse train paradigms.

To provide a control experiment for our findings of varied brain-wide cross-modal BOLD activation patterns driven by VPM stimulations, we examined the BOLD activations upon stimulating a limbic thalamic relay nucleus, medial dorsal thalamus (MD), using identical stimulation paradigms (Histology: Fig. S8; BOLD activation maps: Fig. S9). We found that the 8 Hz 24-pulse stimulation of MD activated brain-wide limbic (MD, mPFC, OFC, RS and EC) and sensorimotor (S1BF, S1Limb, S1ULp, S2, Aud, V1, V2, Ins, PtA, MC, LGN, SC, CPu, GP, SNr & STh) regions, with the strongest activations located at its primary projection target at mPFC. The brain-wide BOLD activations evoked by MD stimulations were also influenced by stimulation frequency and length. Specifically, when compared with the diffusive brain-wide cross-modal BOLD activations (limbic and sensorimotor) evoked at the 8 Hz 24-pulse stimulation, the activations were largely restricted to limbic regions when changing the stimulation frequency (Fig. S9, activated regions: 14 Hz 24-pulse, MD, mPFC, OFC, RS, S1BF, MC and CPu; 4 Hz 24-pulse, MD, mPFC and MC; 20 Hz 24-pulse, MD, mPFC and MC) or length (Fig. S9, activated regions: 8 Hz 16-pulse, MD, mPFC, RS, S1BF, S1Limb, MC, and CPu; 8 Hz 8-pulse, MD, mPFC, MC and GP; 8 Hz 96-pulse, MD, mPFC and MC).

Our results directly reveal brain-wide sensorimotor regions and non-sensorimotor limbic regions as targets of somatosensory thalamic activities evoked at spindle frequencies and lengths (7–15 Hz, 0.5–3 s & 4–25 cycles, especially the 8 Hz 24-pulse stimulation). More importantly, we demonstrate that their cross-modal target locations are frequency- and length-dependent, i.e., both sensorimotor and limbic regions at spindle frequencies and lengths while mainly sensorimotor regions at excessive frequency or length.

### Subcortical and deep cortical LFP recordings reveal spindle-like activities underlying the brain-wide cross-modal BOLD activations

**Fig. 4:**
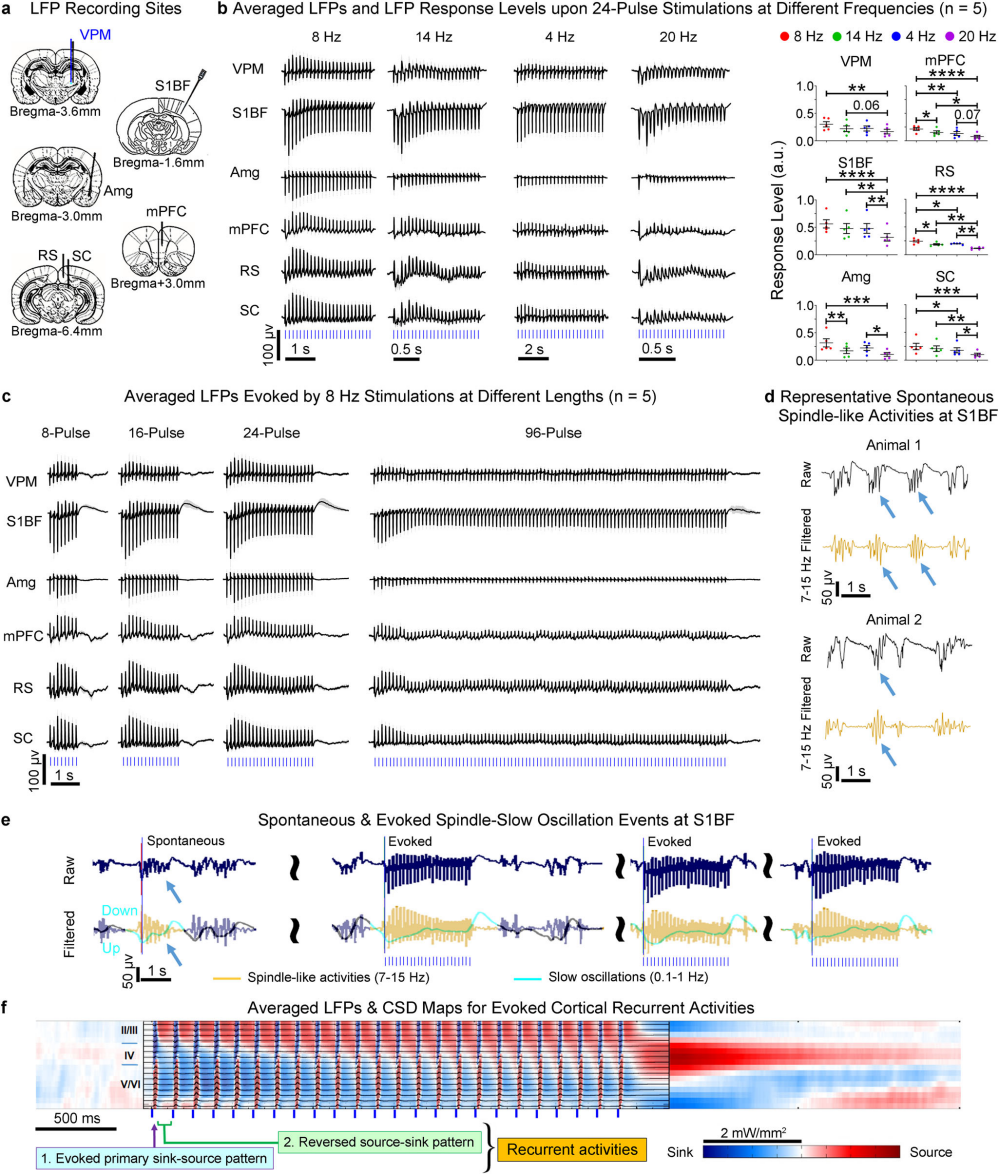
Multisite local field potential (LFP) recordings reveal the spindle-like characteristics of underlying somatosensory thalamically-evoked brain-wide cross-modal neural activities and confirm their dependence on stimulation frequency and length. **a** Illustration of electrophysiology recording sites. **b** Averaged LFPs (left) evoked by 24-pulse stimulations at different frequencies (error bar indicates ± s.e.m.), and quantification of their response level (right). Robust spindle-like activities were evoked at all recorded regions by 8 Hz 24-pulse stimulation (slow spindle frequency and long spindle length). However, evoked LFPs showed decreased response level with changes in stimulation frequency (*n* = 5; error bar indicates ± s.e.m.; one-way ANOVA with Tukey’s post hoc test; *, **, *** and **** denote *P* < 0.05, *P* < 0.01, *P* < 0.001 and *P* < 0.0001) and loss of spindle-shaped waveforms, especially in Amg, mPFC, RS, and SC. **c** Averaged LFPs evoked by 8 Hz stimulations at different lengths (error bar indicates ± s.e.m.). The prolonged stimulation that exceeded spindle length (i.e., 3 s & 25 cycles) evoked weak sustained LFPs that were not predominated by spindle-shaped waveforms. See quantification of response levels in Fig. S12. **d** Representative LFPs with detected spontaneous spindle-like activities (indicated by arrows) in S1BF channel 5 (layer IV). Comparisons between this and the first 1 s of the evoked LFPs (**b** and **c**) showed that the 8 Hz 24-pulse stimulation evoked the most spindle-like LFP signal profiles across all regions. **e** LFPs at S1BF channel 5 in an 8 Hz 24-pulse stimulation trial presented spontaneous and evoked spindle-like activities (raw or 7–15 Hz filtered traces), and spontaneous and evoked slow oscillations (the cyan curve, 0.1–1 Hz filtered). The co-occurrence of evoked spindle-like activities and slow oscillations resembled the spindle-slow oscillation coupling pattern of spontaneous spindle-like activities (indicated by arrows). **f** Averaged LFP traces overlaid with current source density (CSD) maps indicated that recurrent activities evolved with a spindle-shaped profile. Exact *P*-values are provided in Source Data. Source data are provided as a Source Data file.

Guided by our fMRI results, we examined neural activities underlying the varied brain-wide BOLD activation patterns using identical stimulation paradigms (Fig. 1b). We conducted multisite extracellular electrophysiology recordings with 5 single-channel electrodes at ipsilateral VPM, Amg, SC, deep subregions of RS and mPFC, and a multi-depth electrode (16 channels) covering the supragranular to infragranular layers (layers II/III to V/VI) of S1BF (Fig. 4a). Recording locations were strategically chosen based on our fMRI findings of brain-wide cross-modal targets, particularly at subcortical and deep cortical regions, of thalamically-evoked activities at spindle frequencies and lengths (see *Methods* for details). Multi-unit activity (MUA) recorded at VPM showed that all stimulation frequencies successfully evoked spikes in thalamocortical neurons (Fig. S10). Note that only 8 and 14 Hz 24-pulse, and 8 Hz 8- and 16-pulse stimulations evoked rhythmic burst-like activities that corroborated previously recorded spontaneous and optogenetically-evoked spindle activities^6,11,12,41^ within typical frequencies and lengths (7–15 Hz & 0.5–3 s & 4–25 cycles).

We then analyzed the LFP response levels, which are highly correlated with BOLD dynamics^8,42^, and the LFP features to characterize spindle activities^4,10,20^. Averaged bandpass (0.01–200 Hz) filtered LFPs (Fig. 4b and Fig. S11) revealed that the 8 Hz 24-pulse stimulation (slow spindle frequency and long spindle length) evoked robust LFP responses to all stimulation pulses across all examined regions. Specifically, for all the recorded locations, the 2^nd^ to 24^th^ stimulation pulses evoked negative and positive LFP peaks that were comparable to or larger than those evoked by the first pulse. When varying the frequency for 24-pulse stimulations, 14 and 4 Hz stimulations (fast spindle frequency and below spindle frequency, respectively) elicited VPM and S1BF responses at slightly decreased response levels as those evoked by 8 Hz stimulation. Note that the LFP response level for each stimulation paradigm was calculated by averaging the peak-to-peak amplitudes of evoked LFP across all the stimulation pulses. However, significantly weaker responses were evoked in other remote regions (i.e., 14 Hz vs. 8 Hz: Amg, mPFC and RS; 4 Hz vs. 8 Hz: mPFC, RS and SC; one-way ANOVA with Tukey’s post hoc tests, Fig. 4b). Further, the 20 Hz stimulation (i.e., above spindle frequency) evoked significantly weaker responses than the 8 Hz stimulation in all regions (i.e., 20 Hz vs. 8 Hz: VPM, S1BF, Amg, mPFC, RS and SC; one-way ANOVA with Tukey’s post hoc tests, Fig. 4b and Fig. S11). When varying the length for 8 Hz stimulations, 8- and 16-pulse stimulations (i.e., reduced length) evoked similar levels of LFP responses as the 24-pulse stimulation in all examined regions (Fig. 4c and Fig. S11, S12). Meanwhile, the LFP responses to the 96-pulse stimulation (i.e., excessive length) at VPM, S1BF, Amg, mPFC, RS, and SC showed significantly weaker general response levels than those in other stimulation paradigms with fewer pulses. Such decreases in brain-wide response levels due to deviating the stimulation frequency from 8 Hz or excessively increasing the length for 8 Hz stimulation corroborated our fMRI observations (Fig. 2b, c and Fig. 3b, c). These findings indicate that varied brain-wide BOLD activation patterns likely emerge from temporally-specific neural activity patterns (most robust under the 8 Hz 24-pulse stimulation) evoked at the local thalamo-cortical circuit, which subsequently determines long-range cross-modal recruitment of remote targets. Such patterns of evoked LFP responses across all recorded regions remained similar across different light anesthesia levels (Fig. S13) and stimulation light intensities (Fig. S14).

We then examined whether the evoked neural activities share similarities with spontaneous spindle activities in LFP features. Spontaneous thalamo-cortical spindle activities were detected at S1BF recordings with an established amplitude and length thresholding-based automatic spindle detection algorithm^10,20^ followed by visual inspection. The detected spontaneous thalamo-cortical spindle activities in our recordings exhibit well-documented spindle characteristics, such as bursts of oscillatory activities in the spindle frequency range (7–15 Hz); spindle-shaped signal envelopes (i.e., wide in the middle and tapers at both ends), which are especially prominent in the 7–15 Hz bandpass filtered LFP signals; and 0.5–3 s in length (a majority of them are ~1 s) (Fig. 4d, e and Fig. S15)^4,10,20^. Note that, as expected, we detected the co-occurrences of spontaneous spindle activities (7–15 Hz) and slow oscillations (<2 Hz) (Fig. 4e and Fig. S16a). The densities of spindle activities and slow oscillations under 1.0% isoflurane in the baseline period (Pre, prior to any optogenetic stimulation) were 5.34 ± 0.89 events/min (via automatic detection, Fig. S16b) and 12–36 events/min (via visual inspection), respectively, comparable to 2–12 spindle events/min and 10–50 slow oscillation events/min measured in rodent/monkey brains during natural slow-wave or NREM sleep^6–8,32^. We also found that the density of spontaneous spindle activities was similar to baseline (5.88 ± 0.83 vs. 5.34 ± 0.89 events/min; Fig. S16b) when examining periods after 8 Hz 24-pulse stimulation (Post, 27 s period after each 3 s optogenetic stimulation pulse train). Further, stimulations did not alter length and amplitude of spontaneous spindle activities (723.0 ± 28.2 ms vs. 672.5 ± 26.9 ms and 98.20 ± 12.28 μV vs. 96.01 ± 9.20 μV for Post- vs. Pre-stimulation conditions**;** Fig. S16b). The density, length, and amplitude of spontaneous spindle activities also remained unaffected across different light anesthesia levels of 0.0–1.5% isoflurane (Figs. S15 and Fig. S16c). These results demonstrate that spontaneous spindle activities were not diminished and altered in our optogenetic stimulation experiment under light anesthesia conditions.

Notably, the LFPs evoked by the 8 Hz 24-pulse stimulation not only followed the spindle frequency, but also showed the near spindle-shaped envelopes across all recorded locations, especially during the first 1 s of stimulation (Fig. 4b, c and Fig. S11). In comparison, LFPs evoked by 14 and 20 Hz stimulations did not follow the spindle frequency. For example, their 2^nd^, 4^th^, and 6^th^ stimulation pulses did not evoke negative and positive LFP peaks that were comparable to or larger than that evoked by their respective first stimulation pulse (Fig. 4b and Fig. S11). Importantly, LFPs evoked at 14, 4 and 20 Hz stimulations did not exhibit near spindle-shaped waveforms in some of the remote regions (i.e., Amg for the 14 Hz stimulation; Amg and mPFC for 4 Hz; Amg, mPFC, RS and SC for 20 Hz) (Fig. 4b). Meanwhile, decreasing the length for 8 Hz stimulations (i.e., from 24-pulse to 8- or 16-pulse) only shortened LFP response lengths but preserved the spindle-shaped LFP waveforms (Fig. 4c and Fig. S11). However, when increasing the length excessively to 96-pulse, most of the evoked LFPs in all recorded locations were no longer predominated by the spindle-like waveform. Rather, LFPs showed weakened sustained responses, and no spindle-like response was found at Amg (Fig. 4c and Fig. S11). These findings demonstrate that 8 Hz 24-pulse stimulation is an effective paradigm in evoking brain-wide thalamo-cortical spindle-like activities. In addition, other features important for spindle generation were most pronounced in the S1BF LFPs evoked at 8 Hz 24-pulse stimulation, such as co-occurrences of spindle-like activities and slow oscillations (0.1–1 Hz, especially their up-states) (Fig. 4e) and robust recurrent activities over multiple cortical layers evolving with the spindle-shaped profile (Fig. 4f and Fig. S17)^20,25,26^.

Together, our LFP findings reveal the spindle-like nature of neural activities at multiple subcortical and deep cortical regions underlying the brain-wide cross-modal BOLD activations evoked at spindle frequencies and lengths (7–15 Hz & 0.5–3 s, especially the 8 Hz 24-pulse stimulation). We confirm the fMRI findings that the evoked local thalamo-cortical spindle-like activities and their brain-wide cross-modal targeting depend on the temporal characteristics (i.e., frequency and length) of the optogenetic stimulations.

### Somatosensory thalamically-evoked spindle-like activities enhance visual-somatosensory associative memory consolidation

**Fig. 5:**
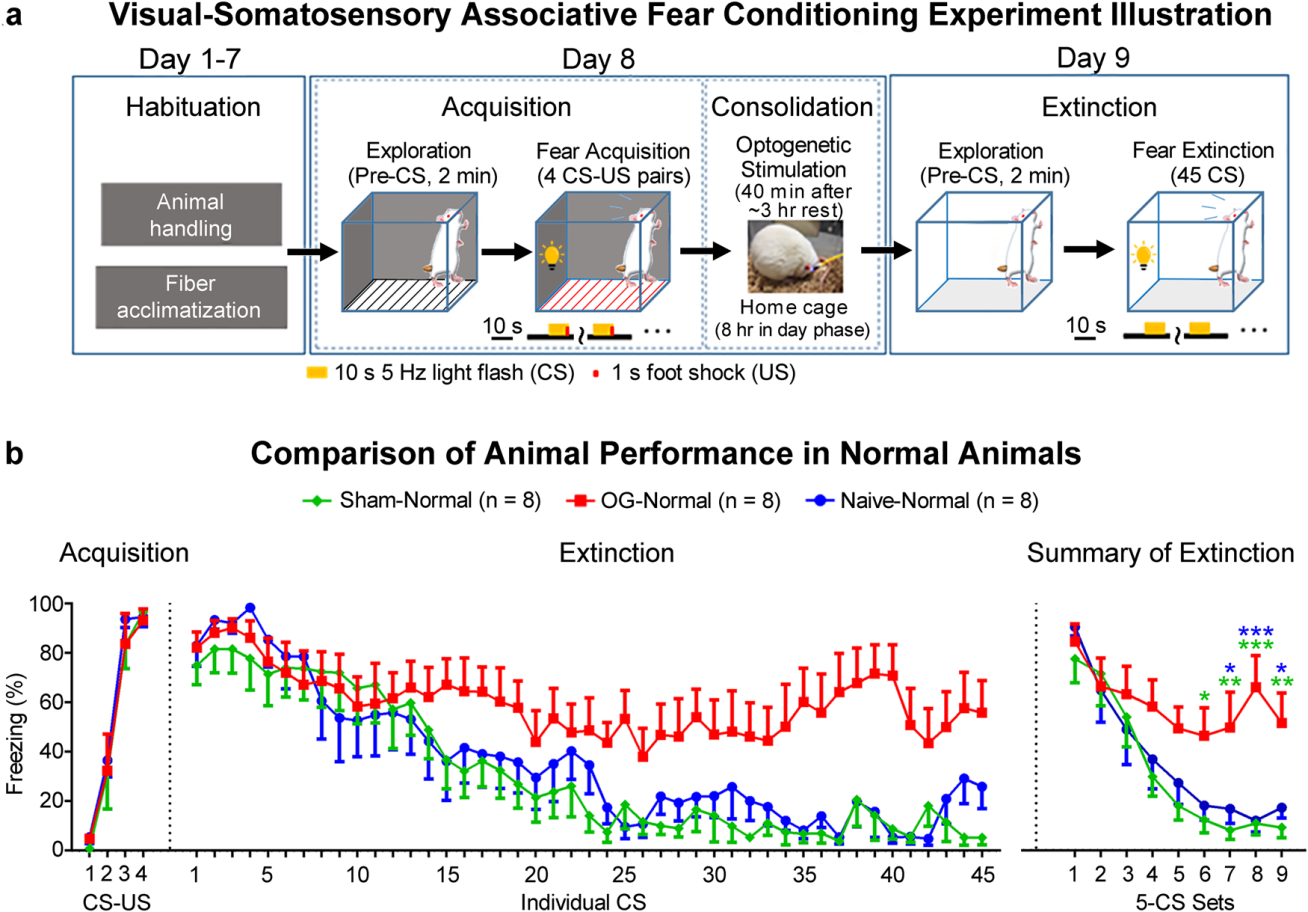
Optogenetically-initiated brain-wide spindle-like activities at VPM enhance visual-somatosensory associative memory consolidation. **a** Fear conditioning experimental design and timeline (CS, conditioned stimulus, 10 s 5 Hz light flash; US, unconditioned stimulus, foot shock). All experiments were performed in day phase. Optogenetics (OG) and Sham animals received 40 min 8 Hz 24-pulse stimulations during memory consolidation. **b** Comparisons of freezing rates in normal animals during acquisition and extinction of visual-somatosensory fear memory (*n* = 8 per group; each CS set represents an average of 5 periodic CS; error bar indicates ± s.e.m.; two-way ANOVA with post-hoc Bonferroni-corrected *t*-tests: *, ** and *** denote *P* < 0.05, *P* < 0.01 and *P* < 0.001, green markers for simple effects between OG vs. Sham, blue markers for OG vs. Naive). Exact *P*-values are provided in Source Data. Source data are provided as a Source Data file.

To examine the effects of evoked brain-wide spindle-like activities on memory consolidation, we tested the memory performances of age-matched optogenetic (OG), Sham, and Naive normal animal groups in fear conditioning experiments (Fig. 5). Animals received 4 pairs of 10 s 5 Hz light flash (CS, conditioned stimulus) co-terminated with a 1 s foot shock (US, unconditioned stimulus) for the acquisition of visual-somatosensory associative fear memory (Fig. 5a). In the following ~8 h where memory consolidation processes associated with spontaneous spindle activities typically occurs^32,43,44^, OG and Sham animals received 8 Hz 24-pulse VPM stimulation once every 30 s for 40 min (Fig. 5a; see *Methods* for details). Note that optogenetic stimulation was only presented after all animals underwent at least 3 h of rest after acquisition of visual-somatosensory associative memory. Animals were observed to stay at quiescent or sleep states (i.e., with eyes closed and curled-up body posture)^45,46^ where spontaneous spindle activities typically occur before and during sleep^6–8,32^. Memory performances (i.e., general memory recall performance and memory strength) dependent on consolidation were assessed in the extinction phase 24 h after memory acquisition by measuring animal freezing levels against the 45 periodic repetitions of CS (i.e., summarized into 9 sets, whereby each is an average of 5 periodic CS; Fig. 5b).

For the acquisition phase, mixed-design two-way ANOVA (Fig. 5b; *n* = 8 per group; post-hoc Bonferroni-corrected *t*-tests) revealed an effect of time (*F*_3,63_ = 111.5, *P* < 0.0001), but not group (*F*_2,21_ = 0.217, *P* = 0.807) nor group × time interaction (*F*_6,63_ = 0.171, *P* = 0.983). This indicates that all groups underwent similar normal fear memory acquisition. At the extinction phase, freezing levels of Sham and Naïve groups progressively decreased (Fig. 5b), in line with reported freezing levels during extinction^47,48^. Importantly, the OG group displayed a significantly slower reduction of freezing levels compared to Sham and Naïve groups [Fig. 5b; *n* = 8 per group; mixed-design two-way ANOVA with: group, *F*_2,21_ = 4.764, *P* = 0.020; set, *F*_8,168_ = 26.76, *P* < 0.0001; group × set interaction, *F*_16,168_ = 2.425, *P* = 0.003; post-hoc Bonferroni-corrected *t*-tests for simple effects between OG-Normal and Sham-Normal (Set6, *P* = 0.036; Set7, *P* = 0.007; Set8, *P* < 0.001; Set9, *P* = 0.006), and between OG-Normal and Naïve-Normal (Set6, *P* = 0.110; Set7, *P* = 0.044; Set8, *P* < 0.001; Set9, *P* = 0.035)]. Such persistent memory in OG animals reflects enhanced memory consolidation associated with spindle-like activities^46^.

These results demonstrate that thalamically-evoked brain-wide spindle-like activities during the consolidation phase enhance the consolidation of cross-modal associative memory.

### Potentiation and recruitment of brain-wide sensorimotor and limbic regions underlie enhanced associative memory consolidation

**Fig. 6:**
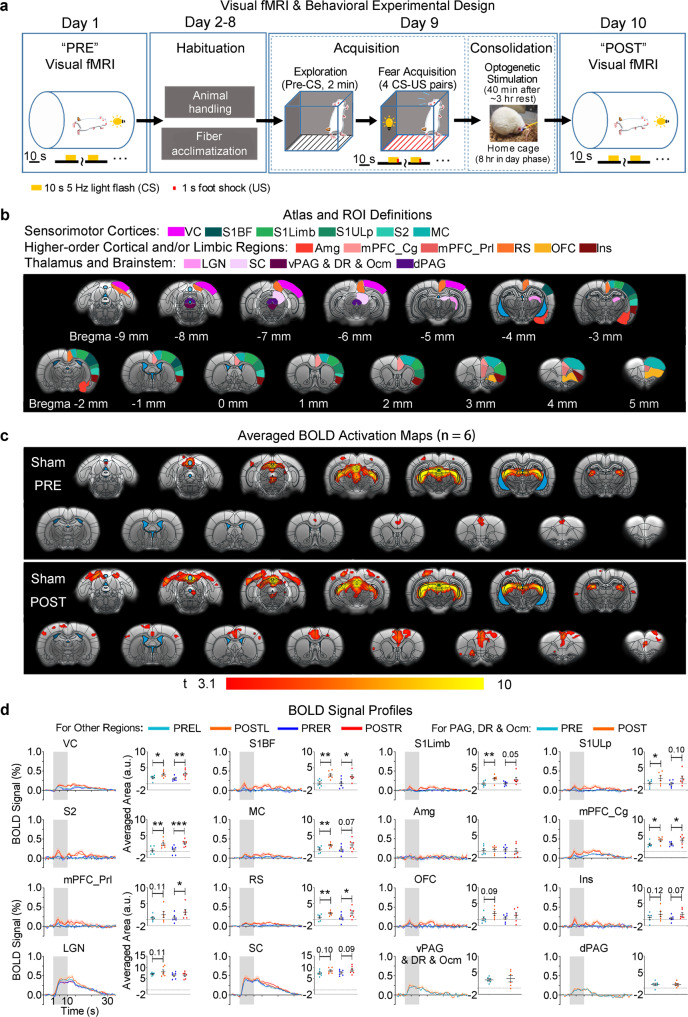
Combined visual fMRI and visual-somatosensory associative fear conditioning experiments in Sham animals (i.e., no optogenetic stimulation) reveal learning and consolidation-dependent enhancement of visual cortical BOLD activations and recruitment of cortical somatosensory, motor, and limbic regions for processing associative fear memory. **a** Visual fMRI-fear conditioning experimental design and timeline, paralleling the behavioral experiments in Fig. 5. **b** Illustration of atlas-based ROI definitions in the sensorimotor regions, higher-order cortical and/or limbic regions, and thalamus and brainstem. Visual cortex (VC), ventral periaqueductal gray (vPAG), dorsal raphe (DR), oculomotor nucleus (Ocm), cingulate and prelimbic cortices in mPFC (mPFC_Cg and mPFC_Prl, respectively), and dorsal periaqueductal gray (dPAG). **c** Sham group-averaged BOLD activation maps and (**d**) profiles and corresponding areas under the signal profiles extracted from ROIs defined in (**b**). The spatial extent of the visual fMRI activation maps and the areas under the curves of BOLD profiles together revealed increased activations in VC and activated somatosensory, motor, mPFC_Cg, mPFC_Prl and RS cortices after memory acquisition and consolidation, indicating a strengthening of brain-wide neural memory representation. (*n* = 6; error bar indicates ± s.e.m.; one-tailed paired *t*-tests; *, ** and *** denote *P* < 0.05, *P* < 0.01 and *P* < 0.001, respectively). This was accompanied by weak trends of activity increase in OFC, Ins, LGN and SC. Exact *P*-values are provided in Source Data. Source data are provided as a Source Data file.

**Fig. 7:**
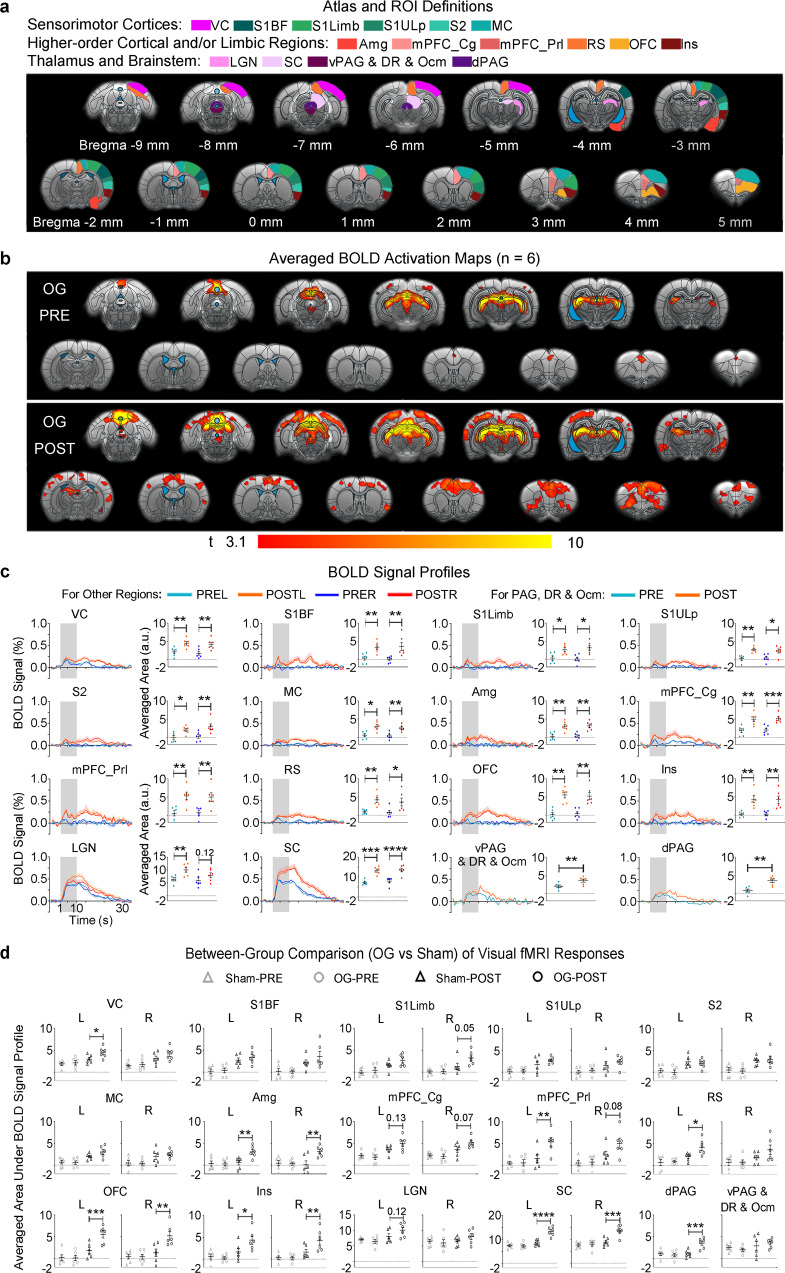
Thalamically-evoked spindle-like activities in optogenetic (OG) animals during memory consolidation phase lead to BOLD response potentiation in brain-wide sensorimotor and limbic regions for strengthening visual-somatosensory associative fear memory representation. The experimental design is shown in Fig. 6a. **a** Illustration of atlas-based ROI definitions in the sensorimotor cortices, higher-order cortical and/or limbic regions, and thalamus and brainstem. **b** OG group-averaged BOLD activation maps and **c** profiles and corresponding areas under the signal profiles extracted from ROIs defined in (**a**). Besides VC, S1, S2, MC, RS and mPFC that were identified in the Sham group, both the spatial extent of the visual fMRI activation maps and the areas under the curves of BOLD profiles showed increased activations in subcortical visual & oculomotor regions (LGN, SC, and vPAG & DR & Ocm) and further recruited sensorimotor midbrain and limbic regions that were not found (dPAG and Amg) or not prominent (OFC and Ins) in Sham animals, indicating further strengthening of neural memory representation (*n* = 6; error bar indicates ± s.e.m.; one-tailed paired *t*-tests; *, **, *** and **** denote *P* < 0.05, *P* < 0.01, *P* < 0.001 and *P* < 0.0001). **d** Between-group comparisons (two-way ANOVA with post-hoc Bonferroni-corrected *t*-tests) of averaged areas under the vfMRI BOLD signal profiles. Between-group comparisons showed that the OG group had significantly stronger enhanced visually-evoked BOLD activations than the Sham group at SC, dPAG, Amg, mPFC-Prl, RS, OFC and Ins in POST condition (*n* = 6 per group; error bar indicates ± s.e.m.; *, **, *** and **** denote *P* < 0.05, *P* < 0.01, *P* < 0.001 and *P* < 0.0001, respectively). See further analyses in Fig. S19. Exact *P*-values are provided in Source Data. Source data are provided as a Source Data file.

We then performed parallel fMRI experiments and investigated the exact large-scale actions of evoked spindle-like activities on fMRI-measured memory activity or neural memory representation (Figs. 6 and 7) that underlie the behaviorally observed enhancement of cross-modal memory consolidation in Fig. 5. Visual fMRI (vfMRI) experiments were conducted 8 days before (PRE) and 1 day after (POST) all animals underwent identical conditioning procedure (Fig. 6a; i.e., habituation, acquisition and consolidation phases) as in the fear conditioning experiments conducted in a separate group of animals (Fig. 5). We employed 10 s 5 Hz light flash identical to the CS as the visual stimuli and kept the animals’ eyes open with tapes in vfMRI. Before (PRE) memory acquisition and consolidation, visually-evoked BOLD activations in both OG and Sham groups were generally restricted to visual and oculomotor-related regions, including the visual cortex (VC), LGN, SC, ventral periaqueductal gray (vPAG) and oculomotor nucleus (Ocm), and cingulate cortex in mPFC (mPFC_Cg) (Figs. 6 and 7). Both groups replicated the memory acquisition pattern in the fear conditioning experiments (Fig. S18; *n* = 6 per group; mixed-design two-way ANOVA with post-hoc Bonferroni-corrected *t*-tests: time, *F*_3,30_ = 66.73, *P* < 0.0001; group, *F*_1,10_ = 0.203, *P* = 0.661; group × time interaction, *F*_3,30_ = 0.284, *P* = 0.837).

After (POST) memory acquisition and consolidation, the Sham group showed increased BOLD activation in bilateral VC (Fig. 6; *n* = 6; ipsilateral VC, *P* < 0.01; contralateral VC, *P* < 0.05; one-tailed paired *t*-tests), accompanied by a trend toward increases in LGN and SC activations (*n* = 6; contralateral LGN, *P* = 0.11; ipsilateral SC, *P* = 0.09; contralateral SC, *P* = 0.10; one-tailed paired *t*-tests). Meanwhile, additional increased visually-evoked BOLD activations were found in cortical somatosensory, motor and limbic regions, which have been shown to be relevant for the processing of visual-somatosensory associative fear memory^49–53^ (Fig. 6; *n* = 6; ipsilateral S1BF, *P* < 0.05; contralateral S1BF, *P* < 0.01; contralateral S1ULp, *P* < 0.05; contralateral S1Limb, *P* < 0.01; ipsilateral S2, *P* < 0.001; contralateral S2, *P* < 0.01; contralateral MC, *P* < 0.01; bilateral mPFC_Cg, *P* < 0.05; ipsilateral prelimbic cortex in mPFC, mPFC_Prl, *P* < 0.05; ipsilateral RS, *P* < 0.01; contralateral RS, *P* < 0.05; one-tailed paired *t*-tests). We noticed that some other regions also exhibited trends toward increased evoked BOLD responses, albeit with weaker amplitudes (Fig. 6d; *n* = 6; ipsilateral S1ULp, *P* = 0.10; ipsilateral S1Limb, *P* = 0.05; ipsilateral MC, *P* = 0.07; contralateral mPFC_Prl, *P* = 0.11; contralateral OFC, *P* = 0.09; ipsilateral Ins, *P* = 0.07; contralateral Ins, *P* = 0.12; one-tailed paired *t*-tests). This result suggests that brain-wide regions including both local sensorimotor cortical regions (VC, S1, S2 and MC) and remote limbic cortical regions (mPFC_Cg, mPFC_Prl and RS) were increased or potentiated to support unaltered cross-modal associative memory consolidation. Other subcortical sensorimotor regions (LGN and SC), cortical sensorimotor and limbic regions (Ins and OFC) likely also participated in such brain-wide memory consolidation.

OG group also showed increased VC activations post memory acquisition and consolidation (Fig. 7; *n* = 6; bilateral VC, *P* < 0.01; one-tailed paired *t*-tests). Notably, we observed significantly increased activations at visual thalamus and midbrain regions, some of which displayed trends of increases in the Sham group (Fig. 7; *n* = 6; contralateral LGN, *P* < 0.01; ipsilateral SC, *P* < 0.0001; contralateral SC, *P* < 0.001; vPAG & DR & Ocm, *P* < 0.01; one-tailed paired *t*-tests). Further, OG group displayed additional increased visually-evoked BOLD activations in various somatosensory, motor, higher-order cortical and limbic regions (Fig. 7; *n* = 6; bilateral S1BF, *P* < 0.01; bilateral S1Limb, *P* < 0.05; ipsilateral S1ULp, *P* < 0.05; contralateral S1ULp, *P* < 0.01; ipsilateral S2, *P* < 0.01; contralateral S2, *P* < 0.05; ipsilateral MC, *P* < 0.05; contralateral MC, *P* < 0.01; dorsal periaqueductal gray, dPAG, *P* < 0.01; bilateral Amg, *P* < 0.01; ipsilateral mPFC-Cg, *P* < 0.001; contralateral mPFC_Cg, *P* < 0.01; bilateral prelimbic cortex in mPFC, mPFC_Prl, *P* < 0.01; ipsilateral RS, *P* < 0.05; contralateral RS, *P* < 0.01; ipsilateral OFC, *P* < 0.01; contralateral OFC, *P* < 0.01; bilateral Ins, *P* < 0.01; one-tailed paired *t*-tests). These regions involved all somatosensory, motor, higher-order cortical and limbic regions that were potentiated in unaltered memory consolidation in the Sham group, including those that previously only exhibited weak potentiation (i.e., ipsilateral S1ULp, ipsilateral S1Limb, ipsilateral MC, contralateral mPFC_Prl, bilateral OFC and Ins) (Fig. 7b vs. Fig. 6c). Among these regions, only Amg and dPAG were not potentiated in Sham animals.

Direct between-group comparisons indicated that the OG group displayed significantly stronger enhancement on visually-evoked BOLD responses than the Sham group over multiple sensorimotor and limbic regions [Fig. 7d; *n* = 6 per group; mixed-design two-way ANOVA with time × group interaction (bilateral Amg, *P* < 0.05; contralateral mPFC_Prl, *P* < 0.05; contralateral RS, *P* < 0.05; ipsilateral OFC, *P* < 0.05; contralateral OFC, *P* < 0.01; bilateral Ins, *P* < 0.05; bilateral SC, *P* < 0.001; dPAG, *P* < 0.05) and FDR post hoc tests for simple effects between OG-POST and Sham-POST (bilateral Amg, *P* < 0.01; contralateral mPFC_Prl, *P* < 0.01; contralateral RS, *P* < 0.05; ipsilateral OFC, *P* < 0.01; contralateral OFC, *P* < 0.001; ipsilateral Ins, *P* < 0.01; contralateral Ins, *P* < 0.05; ipsilateral SC, *P* < 0.001; contralateral SC, *P* < 0.0001; dPAG, *P* < 0.001)]. Some other regions exhibited trends toward stronger enhancement on visually-evoked BOLD responses when comparing the OG with the Sham groups [Fig. 7d; *n* = 6 per group; mixed-design two-way ANOVA with time × group interaction (contralateral VC, *P* = 0.08; ipsilateral S1Limb, *P* = 0.14; bilateral mPFC_Cg, *P* < 0.05; ipsilateral mPFC_Prl, *P* = 0.11; contralateral LGN, *P* = 0.05) and FDR post hoc tests for simple effects between OG-POST and Sham-POST (contralateral VC, *P* < 0.05; ipsilateral S1Limb, *P* = 0.05; ipsilateral mPFC_Cg, *P* = 0.07; contralateral mPFC_Cg, *P* = 0.13; ipsilateral mPFC_Prl, *P* = 0.08; contralateral LGN, *P* = 0.12)]. Note that, as expected, visually-evoked BOLD responses in Sham-PRE and OG-PRE were at similar levels (Fig. 7d; *n* = 6 per group; mixed-design two-way ANOVA with FDR post hoc test). Such further enhancement of vfMRI responses in the OG group compared to Sham group reflects the effect of augmented memory consolidation.

We then examined whether the further enhancement of vfMRI responses in the OG group compared to Sham group (Fig. 7d) shared similar levels of response enhancement as those observed in the Sham group that were caused by learning and consolidation-dependent potentiation effects (Fig. 6d). We treated Sham-PRE and OG-PRE conditions as the baseline condition without prior behavioral training, and compared it with Sham-POST and OG-POST conditions after behavioral training. We found that the further enhancement of vfMRI responses in the OG-POST condition and the increase of vfMRI responses in the Sham-POST condition significantly followed a linearly increasing trend in almost all potentiated regions (Fig. S19; *n* = 6 per group; one-way ANOVA with post-hoc tests for linear trend: *P* < 0.0001: bilateral SC/mPFC_Cg/OFC, ipsilateral Ins, contralateral S1BF/MC/mPFC_Prl/RS and dPAG; *P* < 0.001: bilateral VC/S1Limb, ipsilateral S1BF/S2/mPFC_Prl and contralateral LGN/S1ULp/Amg/Ins; *P* < 0.01: ipsilateral S1ULp/MC/Amg/RS and contralateral S2; n.s.: ipsilateral LGN and vPAG). This finding suggests that evoked spindle-like activities can potentiate brain-wide regions for strengthening cross-modal associative memory representation in similar ways as the learning and memory consolidation processes reflected in the Sham group.

These imaging results directly indicate that associative memory consolidation was mediated through response potentiation across multiple circuits or regions brain-wide. In the presence of optogenetically-evoked spindle-like activities, multiple key sensorimotor (i.e., SC, PAG and Ins) and limbic (i.e., Amg, mPFC-Prl, RS and OFC) integrative regions are further potentiated or recruited, which augmented such large-scale memory consolidation processes at a systems level.

## Discussion

Using optogenetic stimulation, fMRI and electrophysiology recordings, we revealed the previously unidentified, brain-wide cross-modal targeting of somatosensory thalamically-evoked spindle-like activities and their dependence upon stimulation frequency and length. Importantly, our parallel behavioral and visual fMRI experiments demonstrated that cross-modal spindle-like activities facilitated the consolidation of visual-somatosensory associative fear memory by recruiting and strengthening brain-wide memory representation in sensorimotor and limbic integrative regions, especially SC, PAG, Ins, RS and frontal cortices. Together, our findings indicate that sensory thalamo-cortical spindle activities can target brain-wide cross-modal regions in a frequency- and length-dependent manner. They support systems level associative memory consolidation through potentiating key sensorimotor and limbic integrative regions brain-wide to augment memory representation.

In our study, we carefully controlled the stimulation parameters as in the five optogenetic spindle studies by others so far^6,7,11,32,41^, where spindle-like activities were evoked from the thalamus. Our stimulation parameters were also designed with reference to the temporal characteristics of spontaneous spindle activities, such as frequency (7–15 Hz), length (0.5–3 s and 4–25 cycles) and occurrence density (2–12 events/min). Previous optogenetic spindle studies^6,7,11,32,41^ demonstrated the similarities of evoked spindle-like activities to spontaneous spindle activities, mainly through the examination of representative LFP/EEG waveforms^6,7,11,32,41^ and their spectra/spectrogram^6,11,41^, and comparison of behavioral outcomes^6,7,32^, so to generalize their evoked neural activities as spindle activities. We adopted a similar approach to demonstrate that the key features of our optogenetically-evoked spindle-like activities were similar, which include their electrophysiological characteristics at local thalamic and remote cortical/subcortical regions (Fig. 4 and Figs. S10, S11, S13 & S16a), and their functional/behavioral effects on memory consolidation (Fig. 5). Further, unlike the previous optogenetic spindle studies, we performed additional analyses on the optogenetically-evoked spindle-like activities and showed their similarities to spontaneous spindle activities in terms of co-occurring slow oscillations and recurrent activities across multiple cortical layers^4,10,20,25,26^ (Fig. 4e, f and Fig. S17). We also confirmed that the brain-wide propagation characteristics of evoked spindle-like activities were not affected by light anesthesia (Fig. S13) and reduced optogenetic stimulation light intensities (Fig. S14).

Spindle activities occur during sleep and at other states, such as under drowsy, awake quiescence, and light anesthesia states^6–9^. Converging evidence indicates that the influence of anesthesia on characteristics of spindle activities are dosage- and stage-dependent^54–57^. For example, spindle activities are suppressed under deep anesthesia stages (e.g., high-dose isoflurane at >1.5%), but are slightly decreased under light anesthesia (e.g., low-dose isoflurane at 0.5–1.0%)^8,54,56,58,59^. Further, the basic characteristics of spindle activities such as frequency, length and brain-wide responses are largely preserved under light anesthesia states, such as with low dosage isoflurane (0.5–1.0%)^22,60^, dexmedetomidine plus low dosage isoflurane^58^, remifentanil^8^, urethane or ketamine plus xylazine^11,24,61^, and barbiturate^17,61,62^. We found that in our experiments under light anesthesia (0.5–1.0% isoflurane), the spontaneous spindle activity levels (i.e., occurrence density) were not diminished in the baseline period (prior to any optogenetic stimulation) and periods immediately after the stimulation pulse trains (Figs. S13, S15 and S16b, c). In fact, they were comparable to the documented range of spindle density (2–12 events/min) during natural sleep^6,7,11,32^.

Thus, our experimental findings of optogenetically-evoked spindle-like activities observed under light isoflurane anesthesia are highly relevant and sufficiently similar to the natural properties of spindle activities under unanesthetized conditions.

### Brain-wide cross-modal targets of thalamically initiated spindle-like activities

Combining whole-brain fMRI mapping and electrophysiology analyses, our results constitute the first evidence demonstrating that spindle-like activities initiated at a specific sensory thalamic nucleus, especially those at slow spindle frequency and long spindle length, can recruit brain-wide cross-modal targets. Notably, these targets encompass a wide range of regions that were collectively suggested by prior EEG or EEG activity triggered-fMRI mapping studies (i.e., sensorimotor cortical and thalamic regions: S1, S2, Aud, V1, V2, MC, PtA, Ins, VPM, PO, TRN, LGN, and MGB; basal ganglia: CPu, GP & VP, and NAc; limbic regions: mPFC, OFC, HTh, HP)^13–15,17–19^. These regions, and their corresponding systems and modalities targeted by somatosensory thalamo-cortical spindle-like activities are consistent with different functions of spontaneous spindle activities, such as memory consolidation (sensorimotor and limbic regions), sensory gating (sensorimotor regions), and sleep/arousal regulation (basal ganglia)^3,4,29^. Further, our study revealed multiple subcortical and deep cortical targets for somatosensory thalamo-cortical spindle-like activities, which were only implicated individually in invasive electrophysiology studies, including sensorimotor regions—SC^22^, Pir^63^, STh & SNr^24^, and non-sensorimotor limbic regions—Amg^21^, RS^64^, and EC^21,23^. Among these regions, SC, which receives diverse inputs from sensorimotor cortical, thalamic and basal ganglia (e.g., SNr) regions, can be particularly important in subserving spindle activities to mediate functional processes that require multisensory integration and sensorimotor association^22,65^. The remaining targets either belong to or have direct/indirect connections with sensorimotor regions. Specifically, Pir is an olfactory region that has reciprocal connections with Amg and EC^66^. Meanwhile, Amg and EC both have reciprocal connections with other sensory modalities^66^. RS is reciprocally connected to HP, mPFC and VC^64,67^. STh receives diverse inputs from sensorimotor cortices and thalamus, and innervates SNr^24^. These regions may support different spindle-associated functions, such as Pir in sensory gating^66^, STh & SNr in sleep/arousal regulation^24,68^, and Amg, RS and EC in memory consolidation^64,66,67^. Although our optogenetic fMRI results did not provide direct access to memory representations per se, they did capture various regions and networks that were likely involved in different memory representations, which cannot be fully revealed by any simplified memory experiments. For example, while activated regions in basal ganglia (e.g., CPu) are less likely to be involved in representing sensory-related memory compared to the sensorimotor thalamo-cortical regions and limbic regions^49–53^, they could participate in procedural memory (e.g., motor sequences)^27,34,69,70^.

Our results reveal a unique brain-wide cross-modal recruitment property of thalamo-cortical spindle-like activities. Their robust cross-modal recruitment of brain-wide targets (e.g., sensorimotor and limbic) utilized polysynaptic connections, as no ChR2 expression was found in projection synaptic terminals at remote regions other than those expected at somatosensory cortices. Interestingly, such cross-modal recruitment ability also appears to exist in neural activities initiated from a different modality, such as, the limbic thalamic relay nucleus—medial dorsal thalamus (MD), at slow spindle frequency and long spindle length. Specifically, the 8 Hz 24-pulse stimulation at MD also recruited both limbic and sensorimotor regions brain-wide. Note that both MD stimulation and VPM stimulation evoked the most robust activations at their respective primary projection targets (i.e., mPFC in Fig. S9 vs. S1BF in Figs. 2, 3 and Figs. S2, S5) at a similar level. However, compared to brain-wide BOLD activations upon VPM stimulation, the BOLD activations in remote regions upon MD stimulation were generally weaker and less diffusive (i.e., sensorimotor regions for MD, Fig. S9 vs limbic regions for VPM, Figs. 2, 3 and Figs. S2, S5). These results corroborated an idea widely proposed that spindle propagation and distribution are partly circuit-dependent/mediated^4,20,21^. Importantly, the diversity in brain-wide activations despite the similarity in local activation levels at mPFC and S1, respectively, demonstrated that the robust brain-wide propagation of thalamically-evoked spindle-like activities from VPM was not due to animal preparation (e.g., anesthesia) or exaggerated artificial stimulation of local circuits. Overall, our findings suggest that thalamo-cortical spindle activities, especially at slow spindle frequency and long spindle length, may be critical for coordinating information processing and integration across brain-wide neural circuits during different functions, including memory consolidation, sensory gating, and sleep/arousal regulation^3,4,29^.

### Oscillation frequency and length determine brain-wide cross-modal targeting by thalamo-cortical spindle-like activities

Converging studies implicated oscillation frequency and length as important temporal characteristics of spindle activities. For example, both spindle frequency and length change with age or neurological diseases, while spindle length increases following learning^14,15^. These studies implicate the dynamic regulation of spindle frequency and length in normal brain functions. In the healthy brain, slow (e.g., 8 Hz) spindle activities show more global synchronization than the fast spindle activities (e.g., 14 Hz)^14,15,21^, while the length of spindle activities reflects thalamo-cortical network states that could modulate spindle synchronization^11,71^. However, the precise mechanism by which the frequency and length of thalamo-cortical spindle activities influence spatial targeting remains unclear. Our data establish that brain-wide, cross-modal targeting of thalamo-cortical spindle activities depends on their oscillation frequency and length.

We observed robust BOLD activations over brain-wide sensorimotor and limbic regions, and spindle-like LFP responses across all recorded regions (especially during the 1^st^-8^th^ stimulation pulses) during slow spindle frequency stimulations (i.e., 8 Hz 24-pulse). Changing the stimulation frequency from 8 Hz decreased BOLD activations (4 and 14 Hz) or restricted them mainly to sensorimotor regions (20 Hz), and reduced the effectiveness of evoking brain-wide spindle-like LFP activities. This phenomenon likely arises from the critical ~100 ms time window constraining the precise recurrence of consecutive recurrent activities within a thalamo-cortical circuit and the need for sufficient converging inputs into the circuit for spindle generation and synchronization^4,61,72^. For 14 and 20 Hz, recurrent input and output signals evoked at S1BF (i.e., sink and source in current source density maps, CSD, respectively, Fig. S17, corresponding LFPs at Fig. S11) were not facilitated like at 8 Hz stimulation or were partially suppressed (e.g., CSD evoked by the 2^nd^ and 4^th^ stimulation pulses). We concluded that this occurred as the input signal of a consecutive pulse being suppressed by the output signal of the preceding pulse (i.e., CSD sink and source at layer II/III, respectively), which was likely caused by the short-term depression (decreased S1BF and VPM LFP responses to these pulses) associated with short inter-pulse-intervals (i.e., <100 ms)^73^. For 4 Hz, the recurrent CSD activities evoked by the 2^nd^ to 7^th^ stimulation pulses, especially in layers IV-V/VI of S1BF, were not facilitated (Fig. S17). With the long inter-pulse-intervals, the recurrent sink of a preceding pulse did not converge with the primary sink of the consecutive pulse, providing insufficient input for the circuit to facilitate responses. Such non-facilitated or partially suppressed recurrent activities evoked at 14, 4 and 20 Hz stimulations, compared with those in 8 Hz stimulation, indicated the insufficient recruitment of S1BF and TRN neurons in the local thalamo-cortical spindle generation circuit^4,74^. Consequently, 14, 4 and 20 Hz stimulations could not provide robust cortical-TRN inputs for the spindle generation in the local thalamo-cortical circuit^4,17,61,74^ and cortico-cortical outputs for the recruitment of remote brain regions^74^. These findings provide direct evidence that the frequency of local thalamo-cortical activities is critical for both spindle generation within thalamo-cortical circuits and spindle synchronization over brain-wide regions.

Our results showed that the excessive increase of stimulation length above spindle length (i.e., 96-pulse, >3 s & 25 cycles) did not result in enhanced brain-wide BOLD activations but restricted them to mainly sensorimotor regions. We posit that mechanisms modulating both spindle termination and their brain-wide synchronization underlie this phenomenon^11,71,75^. Specifically, we suspect that thalamo-cortico-thalamic and intracortical recurrent interactions crucial for amplifying and sustaining the spatiotemporal synchronization of spindle activities become desynchronized over time, especially during the later phase of a spindle event (e.g., at ~3 s)^71,75,76^. This desynchronization decouples cortical and thalamic firing, restricts spindle propagation, and eventually terminates the spindle event^71,76^. Such desynchronization may appear as weakened thalamo-cortical recurrent activities^71^. In the 96-pulse stimulation, the desynchronization appeared strong and led to a near disappearance of recurrent CSD activities after 3 s of stimulation (Fig. S17), reflecting the typical 5–10 s spindle refractory period^4,17,77^. The weak recurrent activities, especially in layer V/VI of S1BF, were unable to recruit sufficient neurons in the local thalamo-cortical circuit^74^. Further, such desynchronization can progressively depolarize thalamic neurons, which then decreases the necessary thalamic bursting for spindle generation^71^. Altogether, the strong desynchronization at the 96-pulse stimulation resulted in low VPM and S1BF LFP response levels and weakened thalamo-cortical recurrent activities to recruit and synchronize neurons in remote regions^71,74–76^. Note that BOLD activations decreased as expected when reducing stimulation length from 24-pulse to 8- and 16-pulse, which was primarily due to the relationship between BOLD responses and the length of stimulations^78^, not the mechanisms governing spindle termination and synchronization. Our results indicate that both oscillation length and frequency play important roles in driving the brain-wide targeting of thalamo-cortical spindle activities through inherent neural mechanisms underlying spindle generation, synchronization, and termination.

We observed similar temporal characteristic dependence for the brain-wide BOLD activations evoked by MD stimulations (Fig. S9). Compared with cross-modal BOLD activations (limbic and sensorimotor) evoked at 8 Hz 24-pulse, changing the stimulation frequency or length restricted the BOLD activations to limbic regions (Fig. S9). Our results from both VPM and MD stimulations are consistent with the distinction between slow global vs. fast local spindle activities revealed by EEG and invasive electrophysiology studies^14,15,21^. At present, it is only known that the propagation targets of slow vs. fast spindle activities can overlap if they are generated from an identical thalamic nucleus within a thalamo-cortical circuit despite differences in their frequencies (e.g., from VPM within the somatosensory thalamo-cortical circuit). Previous studies also postulated that limbic and sensorimotor thalamo-cortical circuits may separately drive frontal and central-parietal (i.e., sensorimotor cortices) dominance for slow and fast spindle activities, respectively^13–15,21^, neglecting the fact that slow and fast spindle activities could overlap at multiple regions. Our results showed that different spindle-like activities could arise from the same thalamic nucleus (e.g., VPM) and propagate along the same thalamo-cortical circuit differently, with temporal characteristics restricting their propagation distances and spatial extent. They could also arise from different nuclei but reach the same targets depending on their temporal characteristics, as suggested by the overlapping cortical activations for VPM stimulation at fast spindle frequency (i.e., 14 Hz 24-pulse, Fig. 2 and S2) and MD stimulation at slow spindle frequency and long spindle length (i.e., 8 Hz 24-pulse, Fig. S9). Such information cannot be provided by any passive observation approaches in existing spindle mapping studies, highlighting the importance of our approach which integrates whole-brain activity mapping with direct, spatiotemporally-precise initiation of spindle-like activities. Taken together, our findings suggest that despite spindle activities having the identical or different thalamo-cortical initiation circuit(s), their critical temporal characteristics (i.e., frequency and length) drive their subsequent brain-wide targeting sites.

### Strengthening of brain-wide memory representation and integration supports visual-somatosensory associative memory consolidation

Our study demonstrates that spindle-like activities facilitate consolidation of visual-somatosensory associative fear memory over brain-wide sensorimotor and limbic regions. We first found in Sham animals the significant enhancement of visually-evoked BOLD activations, i.e., response potentiation, in local sensorimotor cortical regions (VC, S1, S2 and MC) and limbic cortical regions (Cg and Prl of mPFC and RS) post learning and consolidation. This was accompanied by trends of increases in visually-evoked OFC, Ins, LGN and SC BOLD responses. Such brain-wide response potentiation reflected that the visual-somatosensory associative fear memory representation was stored and consolidated in both local sensorimotor cortices and remote limbic integrative regions^5,27,52,79^. During memory consolidation, these brain-wide regions may be coordinated by spontaneous spindle activities to strengthen the memory representation, where sensorimotor cortices primarily subserve sensorimotor modality-specific memory representation reactivation, while limbic regions mainly support cross-modal representational information reactivation and integration^5,27–29^. Importantly, we revealed the significant potentiation of similar brain-wide regions in the OG group, including those that only showed weak potentiation in Sham animals, and in Amg and PAG, which were not found in Sham animals. While all these regions together constitute a network of brain-wide limbic and sensorimotor regions that corroborate regions collectively revealed by previous associative fear memory studies^49–53,80–82^, our results also indicate that commonly potentiated regions in the Sham and OG animal groups (i.e., mPFC, RS, VC, S1 and S2) likely contain more neural populations involved in associative fear memory representation than other regions. Overall, our results suggest that spontaneous spindle activities and optogenetically-evoked thalamo-cortical spindle-like activities can target and potentiate brain-wide regions in a similar distributed manner to strengthen cross-modal associative memory representation at systems level.

Further, our results also reflect a distinction in these brain regions in directly participating in spindle-associated memory consolidation. When directly comparing OG animals to Sham animals, we discovered significantly enhanced response potentiation in multiple key sensorimotor and limbic integrative regions such as SC, PAG, Ins, Amg, Prl of mPFC, RS and OFC. Such selective enhanced potentiation and newly recruited regions of potentiation in OG animals indicate facilitation of memory consolidation processes following additionally-evoked thalamo-cortical spindle-like activities, e.g. via information integration. Of note, SC is a midbrain structure vital for multisensory integration^65^ and innate fear responses^83^, but surprisingly, has not been implicated in associative memory consolidation. SC may participate in strengthening visual-somatosensory-fear association via its robust large-scale functional interactions with widespread regions mediated by spindle activities^22^. Another key potentiated sensorimotor integrative center in midbrain, PAG, is known to receive projections and integrate information from SC, Amg and mPFC for associative fear memory acquisition and responses^50,51,84^, though has not been directly shown for systems level memory consolidation. Recent studies found that Ins integrates sensory and limbic-related interoceptive information to mediate associative fear memory during extinction^85–87^. The potentiation of Ins in our results suggests that similar processes may support systems memory consolidation during post-learning spindle activities. Besides SC, PAG and Ins, potentiating and recruiting key limbic integrative regions (Amg, Prl of mPFC, RS and OFC) align with the circuits collectively shown to integrate sensorimotor and limbic information in systems consolidation of fear memory^49–53^. Enhanced response potentiation were mainly exhibited in higher-order cortical and limbic regions (e.g., mPFC, RS, OFC, Ins, and Amg) and brainstem sensorimotor integration centers (SC and PAG), but less in primary sensorimotor cortical or thalamic regions subserving sensorimotor modality-specific memory representation^5,27–29^. This is in line with a recent report of the higher general probability of holding memory engrams crucial for memory consolidation in higher-order cortical and limbic regions than primary sensorimotor cortical and thalamic regions^82^. Given the known functions and connections of these regions, we suggest that they could be specifically engaged to integrate inter-regional information into brain-wide knowledge networks for essential cross-modal representation strengthening in associative memory consolidation^5,27,28^. Based on these results, we speculate that inter-regional interactions between these regions and distributed neuronal populations over other key targets of the additionally-evoked thalamo-cortical spindle-like activities participated in such processes^16,32,88,89^. At the systems level, such interactions could be reflected in variations of long-range functional connectivity measured by rsfMRI^18,69,90^ or electrophysiology^23,70^.

rsfMRI constitutes the most robust, quantitative and non-invasive measurement tool for large-scale, brain-wide functional connectivity or networks that can reflect inter-regional integration of memory information^2,18,69,90^. Examining the effects of thalamo-cortical spindle-like activities on brain-wide rsfMRI connectivity will provide important clues about how they may organize inter-regional information integration to promote memory consolidation at systems level^5,27,28^. Despite not providing direct access to memory representations per se as the animals involved did not undergo experimental learning, our preliminary rsfMRI results demonstrate the facilitating effects of somatosensory thalamically-evoked spindle-like activities on brain-wide functional connectivity across their multiple key sensorimotor and limbic targets, and regions potentiated in vfMRI memory tests, including inter-regional connectivity among these regions and their interhemispheric connectivity (Figs. S20 and S21). We note that the enhanced inter-regional rsfMRI connectivity was primarily linked to SC, PAG, Ins, Prl and Cg of mPFC, RS and OFC (Fig. S20b, c). These findings support our notion that these regions are engaged in spindle-associated memory consolidation to integrate inter-regional information. We found that only SC, PAG and Ins among these regions showed solely strengthened inter-regional rsfMRI connectivity, further highlighting the regional specificity of optogenetically-evoked spindle-like activities in facilitating inter-regional information integration to promote associative memory consolidation. In addition, sensorimotor cortices, CPu and HP constitute other key centers of such enhanced inter-regional information integration as expected (Figs. S20b and S21)^50,51^. Recent research indicates the role of EEG-detected post-learning spindle activities in modulating specific long-range HP-cortical functional connectivity in the default mode network associated with memory consolidation^70,90^. Our rsfMRI results also showed that connectivity between regions corresponding to nodes in the default mode network (e.g., mPFC, RS, OFC and HP) were enhanced following evoked spindle-like activities. Our observations revealed the enhancements of memory-evoked activations and rsfMRI connectivity over similar key brain-wide targets of thalamically initiated spindle-like activities, especially in deep and small subcortical regions (i.e., SC and PAG) that are difficult to study in human rsfMRI. Together with our optogenetic fMRI and vfMRI results, we propose that thalamo-cortical spindle-like activities not only engage sensorimotor cortical neuronal populations within their brain-wide targets for memory representation strengthening, but also modulate large-scale inter-regional functional connectivity over key targets to shape information integration.

Spindle activities change with age and may be used as a marker of aging-related memory deficits^14,16,35^. Our preliminary results showed a decrease of general memory recall performance in accelerated aging^91^ animals (i.e., Sham-Aging animals in Fig. S22b vs. Sham-Normal animals in Fig. 5b during the extinction phase), indicating impaired memory consolidation functions. Importantly, optogenetically-evoked spindle-like activities improved the general memory recall performance of accelerated aging animals (Fig. S22b), likely involving similar brain-wide targets as in normal animals (Fig. S23 vs. Figs. 2, 3 and Figs. S2, S5). These results reveal that targeted initiation of brain-wide spindle-like activity propagation from somatosensory thalamus can alleviate aging-related memory consolidation deficit. Our findings in normal and accelerated aging animals together suggest that thalamo-cortical spindle activities and the regions (e.g. SC and mPFC) involved in spindle-associated memory consolidation could be potential therapeutic targets in aging-related memory deficits^16,35^.

In summary, our results demonstrate that thalamically-evoked spindle-like activities propagate to numerous sensorimotor and non-sensorimotor limbic regions in a frequency- and length-dependent manner. We show that these spindle-like activities promote cross-modal associative memory consolidation by potentiating and engaging a subset of key integrative regions brain-wide. Together, our findings reveal the cross-modal spatiotemporal targeting characteristics of thalamo-cortical spindle activities. We further discover their causal actions during memory consolidation at systems level. Our study also demonstrates the initiation of low frequency oscillatory activities brain-wide as a potential intervention to enhance memory performance or rescue memory decline in future studies.

## Methods

### Animal subjects

All animal experiments were approved by the University of Hong Kong’s Committee on the Use of Live Animals in Teaching and Research (CULATR). Eleven groups of age-matched adult male Sprague Dawley rats were used in this study (i.e., Six groups comprising of VPM optogenetically transfected animals, one group comprising of MD optogenetically transfected animals, and four groups are normal animals). Five of optogenetically transfected animal groups underwent MRI experiments (optogenetic fMRI-VPM: *n* = 16 for testing different stimulation frequencies at 24-pulse length, *n* = 10 of them were used for testing different stimulation lengths at 8 Hz; and *n* = 7 of them for testing fMRI responses in accelerated aging animal model after behavioral experiments; optogenetic fMRI-MD: *n* = 6; visual fMRI/vfMRI: *n* = 6; optogenetic rsfMRI: *n* = 6), one group underwent pure behavioral experiments (fear conditioning: *n* = 8), whereas the other group underwent electrophysiology recording experiments (optogenetic: *n* = 5, batch 1). Three groups of normal animals were used as control animals (*n* = 8 for each Sham- and Naïve-Normal group, and *n* = 7 for Sham-Aging group) in the fear conditioning experiments, while the other group was used as Sham control animals in the vfMRI experiments (*n* = 6). Additionally, a separate group of optogenetically transfected animals underwent control electrophysiology experiments under different light anesthesia levels and at different optogenetic stimulation light intensity levels (*n* = 4, batch 2). We did not consider sex in our experiments and did not perform sex-based analysis in this study as no consensus has been made in the field on the sex differences of spindle activities and their effects on memory consolidation.

### Virus packaging, and stereotactic surgery for viral injection

Recombinant Adeno-associated virus (AAV) vectors were serotyped with AAV5 coat proteins and produced by the vector core at the University of North Carolina at Chapel Hill, Chapel Hill, NC. Viral titer (in particles per milliliter) was 4 × 10^12^ for AAV5-CaMKIIα-ChR2(H134R)-mCherry. Maps are available online from http://www.stanford.edu/group/dlab/optogenetics.

All stereotactic surgeries for viral injection were performed with rats at 6–7 weeks of age. Rats were anesthetized with an intraperitoneal bolus injection of a ketamine (90 mg/kg) and xylazine (40 mg/kg) mixture. Following a small craniotomy, viral injections were performed at two depths in VPM (–3.6 mm posterior to Bregma, +3.0 mm medial-lateral right hemisphere, –6.0 and –6.3 mm from the surface of dura) or in MD (–2.8 mm posterior to Bregma, +0.9 mm medial-lateral right hemisphere, –5.2 and –5.5 mm from the surface of dura). Three microliters of viral constructs (1.5 μL at each depth) were delivered through a 5-μL syringe and 33-gauge beveled needle at 150 nL/min. The injection needle was held in place for 10 min before being slowly retracted from the brain. Scalp incision was sutured, and animals were kept on a heating pad until recovery from anesthesia. Buprenorphine (0.05 mg/kg, subcutaneous) was administered post-injection twice daily for 72 h to minimize discomfort. Enrofloxacin was also administered orally for 72 h to minimize infection and inflammation post-surgery. Animals recovered for 4–5 weeks before conducting MRI, electrophysiology and behavioral experiments.

### Animal preparation for optogenetic fMRI, vfMRI, and rsfMRI experiments

Surgery was performed under 1.2–2.0% isoflurane to implant an opaque custom-made plastic optical fiber cannula (*d* = 450 μm) at VPM/MD with subsequent high-resolution anatomical MRI verification under 1.0–1.2% isoflurane maintenance^38–40,92,93^. Animals were only stabilized at 2% isoflurane during the first 15 min of surgery for craniotomy to minimize the duration of exposure to high dosage anesthesia. The animals were subsequently maintained with 1.2–1.5% isoflurane for the remaining 15–30 min surgical procedure (i.e., dura removal and optical fiber implantation). The animals were then maintained under 1.0–1.2% isoflurane during MRI setup and anatomical scans. Note that the fiber cannula was made opaque using heat-shrinkable sleeves to prevent light leakage during optogenetic stimulation^38–40,92,93^ and the eyes of the animals were blindfolded in optogenetic fMRI and rsfMRI experiments to avoid undesired visual stimulation^92,94,95^. Animal eyes were kept open with tapes in the vfMRI experiments. Our previous studies have demonstrated that such preparation protocol with eyes either blindfolded^92^ or uncovered^39^ showed no evoked neural activities and fMRI responses in the visual pathway during light delivery to VPM for optogenetic stimulation in opsin-free control animals. The fiber tip surface was beveled to facilitate fiber insertion and minimize injury to brain tissue. Dental cement was applied to fix the fiber cannula on the skull. The optical fiber tip was typically situated in the center of the VPM/MD nucleus through stereotaxic implantation and verified by high-resolution anatomical MRI. The spatial spread from the fiber tip for 473 nm blue light is rather small (200 μm and 350 μm at 50% and 10% of initial light intensity, respectively) and has little or no spreading in the backward and lateral directions^96^, confining the optogenetic stimulation within the VPM/MD nucleus. Buprenorphine (0.05 mg/kg, subcutaneous) was administered post-implantation subcutaneously, and one drop of 2% lidocaine was applied to the chords to minimize discomfort before endotracheal intubation and MRI experiments. The animals were mechanically ventilated at a rate of 60 breaths per minute with 1.0% isoflurane in room-temperature air using a ventilator (TOPO, Kent Scientific, Torrington, CT). During all fMRI experiments, animals were placed on a plastic holder, and their heads were fixed with a tooth bar and ear bars. Continuous physiological monitoring was performed using sensors of an MRI-compatible system (SA Instruments, Stony Brook, NY). Rectal temperature was maintained at ∼37.0 °C using a water circulation system. Vital signs were within normal physiological ranges (rectal temperature: 36.5–37.5 °C, heart rate: 350–420 beats/min, breathing: ~60 breaths/min and not synchronized to optogenetic stimulation, oxygen saturation: >95%) throughout the length of the experiments^38,39,93^.

### MRI scanner-synchronized optogenetic and visual stimulation

An Arduino programming board synchronized the scanner trigger and the lasers for optogenetic and visual stimulation. Computers and light delivery systems were kept outside the magnet, and optical patch cables (5–10 m) delivered light into the bore of the scanner. For optogenetic stimulation, blue light was delivered using a 473 nm Diode-pumped solid-state laser measured before scanning as 8 mW at the fiber-tip (450 μm, NA = 0.5) corresponding to a light intensity of 40 mW/mm^2^. For visual stimulation, blue light was delivered using a 473 nm DPSS laser via a separate optical patch cable. The emitted light was measured before the start of scanning as 0.5 mW at the fiber tip (400 μm, NA = 0.39). Laser stimulation protocols varied depending upon the fMRI experiment parameters.

To examine the brain-wide propagation and targeting characteristics of thalamo-cortical spindle activities, stimulation pulse trains with varied frequencies (4, 8, 14, and 20 Hz for 24-pulse) or lengths (8-, 16-, 24-, and 96-pulse for 8 Hz) were used (light intensity = 8 mW: 40 mW/mm^2^; 10 ms pulse width). We covered slow and fast spindle frequencies (8 and 14 Hz, respectively), and frequencies below or above (4 and 20 Hz, respectively) the typical range of spindle frequency. Lengths for 8 Hz stimulations were chosen within or above the typical range of spindle length (i.e., 1, 2, 3, or >3 s, equal to 8, 16, 24, or >25 cycles). Two sessions were acquired for each stimulation pulse train paradigm. The sequence of varied stimulation paradigms was randomized across animals. For each session, the same stimulation pulse train was repeated every 30 s. The interval between two consecutive stimulation pulse trains was set as 30 s to ensure that the density of the evoked spindle-like activities was within the natural density range of spontaneous spindle activities (2–12 events/min)^10,21,32^. Note that identical optogenetic stimulation paradigms were employed for VPM and MD experiments.

To examine whether and how optogenetically-evoked spindle-like activities act on brain-wide functional responses to memory consolidation-associated visual stimulation (i.e., memory representation), vfMRI experiments were conducted 8 days before (PRE) and 1 day after (POST) animals underwent the same habituation, acquisition and consolidation phases as in the fear conditioning experiments. For optogenetic stimulation, 8 Hz 24-pulse paradigm delivered once every 30 s was used as it is the most effective paradigm in evoking brain-wide spindle-like activities. For visual stimulation, 10 s 5 Hz light flash was employed (light intensity = 0.5 mW: 50% duty cycle). Blue light flashing at 5 Hz was delivered via an optical fiber placed 3.5 cm in front of the eyes for binocular visual stimulation in vfMRI. The vfMRI paradigm used consisted of six blocks of 10 s visual stimulation and 30 s rest. Sixteen sessions (eight for PRE, eight for POST) were acquired in total for each animal.

To probe the effects of optogenetically-evoked spindle-like activities on brain-wide long-range rsfMRI connectivity, a total of eight sessions were acquired for each animal, four before (PRE) and four after (POST) optogenetic stimulation. For each session of optogenetic stimulation (OG-On), the 8 Hz 24-pulse stimulation paradigm was employed.

### MRI acquisition procedure

All MRI experiments were performed on a 7 T MRI scanner (ParaVision v5.1, PharmaScan 70/16, Bruker Biospin GmbH, Ettlingen, Germany) using a transmit-only birdcage coil in combination with an actively decoupled receive-only surface coil. A single channel receive-only surface coil was used for the optogenetic fMRI, vfMRI, and rsfMRI experiments. After placing the animal in the magnet, scout and anatomical RARE T2-weighted (T2W) images were first acquired for accurate positioning and reference with field of view (FOV) = 32 × 32 mm^2^, matrix = 256 × 256, RARE factor = 8, echo time (TE) = 36 ms, repetition time (TR) = 4200 ms. Sixteen contiguous 1.0-mm slices were positioned in the transverse orientation according to the rat brain atlas^38–40^ to cover the majority of the brain. All fMRI, vfMRI, and rsfMRI data were obtained at the same geometry as the anatomical reference T2W images, using a single-shot gradient-echo echo-planar imaging (GE-EPI) sequence with FOV = 32 × 32 mm^2^, matrix = 64 × 64, flip angle = 56° (optogenetic fMRI and vfMRI) or 50° (rsfMRI), TE = 20 ms, TR = 1000 ms (optogenetic fMRI and vfMRI) or 750 ms (rsfMRI). A total of 600 s volumes were collected during each fMRI or rsfMRI session, while 270 s volumes were acquired for each vfMRI session.

### Optogenetic fMRI, vfMRI, and rsfMRI data analyses

All fMRI/rsfMRI preprocessing was performed using the standard procedure established in our previous studies^38,39,93^ For each fMRI/rsfMRI session, all EPI images were first corrected for slice timing differences and then realigned to the mean image of the first fMRI/rsfMRI session using SPM12 fMRI toolbox (Wellcome Department of Imaging Neuroscience, University College London, UK). fMRI scans suffering from motion artifacts (>0.05 mm voxel shifts detected by realignment) and sudden physiological changes (i.e., abrupt changes in respiration pattern, heart rate, and oxygen saturation level) were discarded. After resampling EPI and T2 images to 128 × 128 × 16, the EPI images from the same animal were registered to their T2W images before the T2W images from each animal were coregistered to a representative brain using affine transformation and Gaussian smoothing to maximize normalized mutual information (SPM12). The transformation matrix was then applied to coregister fMRI/rsfMRI EPI images. Voxel-wise linear detrending with least-squares estimation was subsequently performed temporally to eliminate the baseline drift caused by physiological noises and system instability.

For optogenetic fMRI, data from repeated fMRI sessions were averaged within animals, in-plane smoothed [full width at half maximum (FWHM) = 1 pixel], and high-pass filtered (128 s). A voxel-wise coherence analysis^97^ was applied for optogenetic fMRI data, in which coherence value was defined as the magnitude of the frequency component of interest/frequency of block (|F(f0)|, f0 is 1/30 in our experiment), divided by the sum-of-squares of all frequency components $\sqrt{{\sum}_{f}|{F(f)}|^2}$. The coherence value is between 0 and 1, which can be converted to *P*-values. Employing *P* < 0.001 thresholding (coherence value >0.135) then yielded the BOLD activation maps upon optogenetic stimulation for each paradigm/condition. After comparison of activation maps between animals to ensure result quality, realigned, registered, and resliced fMRI images corresponding to the same stimulation paradigm were averaged across animals. Similar smoothing, filtering, and coherence analyses were applied to map group-averaged activations. BOLD signal profiles were extracted from atlas-based ROIs for further evaluation and comparison of BOLD activations. The statistical threshold for the averaged BOLD activation coherence maps was selected to ensure their consistency with ROI extracted BOLD signal profiles. Averaged BOLD activation maps were then overlayed with group-level masks generated from one-sample group level *t*-tests using nonparametric inference with threshold-free cluster enhancement^98,99^ multiple comparison correction of family-wise-error rate (TFCE-FWE, *P* < 0.05) or from Bonferroni correction (*P* < 0.05). Statistical comparisons for the BOLD activations were performed between 8 Hz 24-pulse stimulation and other stimulation paradigms using two-sample *t*-tests with Gaussian random field (GRF, voxel level *P* < 0.05 and cluster level *P* < 0.001)^100^ or TFCE-FWE (*P* < 0.05) corrections.

For vfMRI, data from repeated fMRI sessions were averaged within-animal, in-plane smoothed [FWHM = 1 pixel], and high-pass filtered (128 s). A general linear model (GLM) was applied to calculate the activation coefficient (β) maps for each stimulus. Student’s *t*-test was performed to identify activated voxels using the threshold *P* < 0.001 (*t* value >3.1). After comparison of activation maps between animals to ensure result quality, one animal that showed weak vfMRI activations was excluded from further analyses and experiments. Realigned, registered and resliced images corresponding to the same fMRI condition (i.e., PRE vs. POST) were averaged across animals. Similar smoothing, filtering and GLM processes were applied to map group-averaged activations. BOLD signal profiles for each condition/animal group were extracted from identical regions of interest (ROIs) delineated from the rat brain atlas. The activation strength of the BOLD signal profile in each ROI was then quantified by computing the area under the profile. Comparisons between conditions (i.e., PRE vs. POST) were done using one-tailed paired *t*-tests to identify the learning-dependent (or learning & optogenetic stimulation-induced) changes in fMRI activations to visual stimuli in each animal group. Between-group comparisons were done by performing two-way ANOVA with FDR post hoc tests to reveal regions that showed stronger activations to visual stimuli in the OG group than in the Sham group. One-way ANOVA with post-hoc tests for linear trend was employed to examine whether the further enhancement of vfMRI responses in the OG group compared to Sham group shared similar levels of response enhancement as those observed in the Sham group caused by learning and consolidation-dependent potentiation effects. Note that, to examine between-group differences, post-hoc tests of visual fMRI data employed an FDR procedure using the areas under the BOLD signal profiles in each individual ROI. The FDR correction was used to control the multiple comparisons for different groups and conditions, not for different brain regions. FDR procedure was chosen to best reflect the differences shown in the BOLD signal profiles. This procedure was further controlled by cross-validation between the activation maps and BOLD signal profile analysis to avoid erroneous detection of between-group differences and subsequent misinterpretation.

For rsfMRI data acquired PRE and POST optogenetic stimulation, a temporal band-pass filter (0.005–0.1 Hz) without spatial smoothing was applied. Global signal regression was applied for removing non-neuronal global variance. To examine functional connectivity changes before (PRE) and after (POST) optogenetically evoking spindle-like activities, seed-based analyses were applied to map and quantify rsfMRI connectivity for primary somatosensory (S1), secondary somatosensory (S2), visual (VC), auditory (Aud), motor cortex (MC), cingulate (Cg), retrosplenial (RS), prelimbic (Prl), orbitofrontal (OFC), insular (Ins) cortices, superior colliculus (SC), lateral geniculate nucleus (LGN), amygdala (Amg), periaqueductal gray (PAG), caudate putamen (CPu), hippocampus (HP). For interhemispheric rsfMRI connectivity in S1, S2, VC, Aud, MC, Cg, RS, Prl, OFC, Ins, SC, LGN, Amg, CPu, and HP, two atlas-defined 2 × 2-voxel regions were chosen as the ipsilateral and contralateral seed, respectively. For rsfMRI connectivity in PAG, two seeds were chosen from its dorsal and ventral divisions. For better visualization of the inter-regional connectivity maps between Cg and RS, ipsi- and contralateral seeds were combined into Cg-Bilateral and RS-Bilateral seeds. Reference signal profiles were generated by calculating the regionally averaged signals from the voxels within each seed. For every single rsfMRI session, Pearson’s correlation coefficients (CCs) were calculated between the reference signal profiles and the BOLD signals of every other voxel to generate correlation coefficient maps (CC maps) for each seed location. CC maps from repeated rsfMRI sessions were averaged within animals to generate individual CC maps. After comparisons of individual CC maps between animals to ensure result quality, the group-averaged rsfMRI functional connectivity maps were generated by averaging individual CC maps across animals and applying a CC-threshold (CC > 0.1, corresponding to *P* < 0.05) on the group-averaged CC maps. Subsequently, 6 × 6-voxel ROIs centered on each seed location were then used to extract the CC values from the connectivity maps (e.g. contralateral S1 ROI for maps generated using ipsilateral S1 seed). For the bilaterally-combined Cg or RS seeds (i.e., Cg-Bilateral and RS-Bilateral), the ROIs were combined from their ipsi- and contralateral 6 × 6-voxel ROIs. Interhemispheric/inter-regional rsfMRI functional connectivity was then quantified by averaging the CC values of the corresponding two ROIs. Following one-sample *t*-tests (*P* < 0.05) within each condition (i.e., “Pre” and “Post”), two-tailed paired *t*-tests with FDR correction (*P* < 0.05) were then applied to identify the significantly altered rsfMRI connectivity. FDR correction was employed for the rsfMRI connectivity pairs that showed significant CC values not equal to zero in one-sample *t*-tests (i.e., less than 34 x 34 ROI pairs).

### In vivo electrophysiology experiments and data analyses

Electrophysiology recordings were performed under the same anesthesia protocols and similar physiological conditions as in the fMRI/rsfMRI experiments. Craniotomies were made, and dura matter was removed with reference to ipsilateral S1 barrel field (S1BF, –1.6 mm anterior to Bregma, +5.4 mm ML), Amg (–3.0 mm anterior to Bregma, +5.0 mm ML), mPFC (+3.0 mm anterior to Bregma, +0.6 mm ML), and RS & SC (–6.4 mm anterior to Bregma, +1.0 mm ML). Amg, RS, and SC were chosen to confirm the first-time detection (i.e., using BOLD fMRI mapping) of deep and/or small regions (i.e., Amg, SC, Pir, RS, EC, STh & SNr; Fig. 2) as targets of thalamo-cortical spindle-like activities. Among them, SC represented the subcortical sensorimotor regions, while Amg and deep subregions in RS (granular area) and mPFC (prelimbic area) represented the remaining subcortical and deep cortical limbic regions, respectively. mPFC—the prominent anterior projection target of limbic thalamic nuclei^101^, was chosen to echo RS (posterior cortical limbic region) for the purpose of verifying the robust cross-modal recruitment of cortical limbic regions over anterior and posterior brain. To cover cortical layers II/III to V/VI at ipsilateral S1BF, a linear microelectrode silicon array (16 recording channels equally spaced at 100 μm; 1.5 MΩ impedance; NeuroNexus Technologies, Ann Arbor, MI) was inserted perpendicular to the cortical surface approximately 2.5 mm below the dura matter with micromanipulators (Narishige, Amityville, NY). For other regions, single-channel electrodes with 2.0 MΩ and 10 μm tip diameter were implanted (Amg: –8 mm DV; mPFC: –3 mm DV; RS: –2.0 mm DV; SC: –3 mm DV). Fiber and a single-channel electrode were constructed into an optrode and were implanted followed identical procedures as described for optogenetic fMRI/rsfMRI experiments. S1BF recordings were acquired using a multi-channel neurophysiology recording system (sampling frequency: 24 k Hz; notch-filter: 50 Hz, 100 Hz, and 150 Hz; Synapse v86, Tucker Davis Technologies/TDT, Alachua, FL). Synchronized laser stimulation was controlled by the same system, and light pulses were recorded simultaneously with the neural data. Electrophysiological data from VPM, Amg, mPFC, RS, and SC were acquired with a 32-channel OpenEphys data acquisition system (v0.4.4.0, digitized at 30 k Hz and bandpass filtered between 0.1–8 k Hz). The experimental paradigms were the same as used in optogenetic fMRI experiments and stimulations were controlled by the TDT system.

After recordings, raw data from VPM were bandpass filtered at 300–8 k Hz to generate MUA traces^38,39^ and to determine whether the stimulations at all frequencies successfully excited the VPM thalamocortical excitatory neurons. Raw data from all recordings were bandpass filtered (0.01–200 Hz) and down-sampled to 1 k Hz for LFP data analyses using MATLAB R2018a. After confirming the reproducibility of evoked neural activities across the stimulation blocks within each animal, LFPs were averaged across blocks to generate a single LFP trace for each stimulation paradigm. They were then averaged across animals. Averaged LFP traces were presented with standard error mean to reflect the reproducibility across animals. As shown by our CSD analyses and literature^93,102–104^, the layer IV in S1BF is the location that first received VPM thalamic input, therefore, the LFPs from layer IV were selected to visualize the evoked spindle-like activities and spontaneous spindle-like activities in S1BF. To quantify the LFP response levels for different stimulation frequencies, peak to peak amplitudes for the LFP responses to each stimulation pulse were measured and averaged across pulses for each paradigm. Comparisons between different paradigms were done by performing one-way ANOVA followed by Tukey’s post hoc test.

To detect spontaneous thalamo-cortical spindle-like activities, an established amplitude and duration thresholding-based automatic spindle detection algorithm^10,20^ was employed for S1BF LFP data, followed by visual inspection. Specifically, the LFP data were first bandpass filtered at 7–15 Hz spindle frequency, and the envelope was determined by calculating the root mean squared moving averaged signals with a 400 ms moving window. The mean (µ) and standard deviation (σ) of the envelope signal were calculated. Potential spindle-like events were detected when the envelope exceeded the duration threshold defined as µ + 1.5 × σ for at least 500 ms but not longer than 3 s and the amplitude threshold defined as µ + 2.5 × σ for at least one data point. Visual inspection was then performed to ensure that the detected spindle-like events exhibited the spindle-shaped signal envelopes (i.e., wide in the middle and tapers at both ends), especially in the 7–15 Hz bandpass filtered LFP signals. To detect slow oscillations that co-occurred with spindle-like activities, LFP signals were bandpass filtered at 0.1–1 Hz and subsequently overlaid with the spindle band (7–15 Hz bandpass filtered) signals. LFP power spectral density was calculated via Welch’s method with 2 s windows and 50% overlap using the *pwelch* function in MATLAB. One-way ANOVA with post-hoc Bonferroni-corrected *t*-tests was employed for statistical comparisons of density, duration, and amplitude of detected spontaneous spindle activities across different conditions.

We employed high-resolution CSD analysis to identify spindle-associated cortical layer-specific activities in S1BF LFP data. In brief, CSDs were calculated as the second spatial derivative of LFPs and visualized by color-coded plots with linear interpolation^102,104^. Layer estimation for ipsilateral S1BF was performed upon determining the optogenetically evoked prominent primary CSD sinks at ~13 ms after stimulation onset in the middle channels and defining layer IV by the 4 channels covering the primary sink^93,102–104^. Therefore, each stimulation pulse evoked primary CSD sink at layer IV, indicating that the thalamic input signal first arrived at layer IV. This corroborates literature that the majority of cortical spindle activities arise from middle and deep cortical layers^25,26^. The primary evoked vertically-positioned sink-source layer distribution pattern showed sink at layer IV shifted to layer II/III, and source at layer V/VI before ~50 ms, indicating the involvement of multiple cortical layers in intracortical (layer IV → layer II/III) and cortico-thalamic (layer V/VI → thalamus) communication during spindle generation. The recurrent activities were determined by observing a reversed vertically-positioned source-sink layer distribution pattern compared with the primary evoked one: the sink was transferred to layer V/VI, and source to layer II/III after 50 ms. Averaged LFPs were overlaid with CSD to visualize the concurrence of the spindle-shaped profiles in LFPs and CSD activities.

### Visual-somatosensory associative fear conditioning experiments and data analyses

Before OG or Sham animals underwent fear conditioning experiments, chronic fiber implantation was performed using similar procedures that were utilized for the MRI experiments. Animals recovered for 1 week and subsequently underwent 15 min of animal handling and fiber acclimatization for consecutive 1 week in the habituation phase. Naïve animals also underwent the same habituation phase.

Memory acquisition and extinction were performed in day phase using a startle and fear conditioning system (Panlab Harvard Apparatus, Massachusetts, USA). During the acquisition phase, each animal was first placed in a black chamber with grid floor. After 2 min of exploration and adaptation, the animal received 4 pairs of 10 s 5 Hz light flash (CS, conditioned stimulus) co-terminated with a 1 s 0.6 mA foot shock (US, unconditioned stimulus) at randomized intervals of 75 to 135 s for the acquisition of visual-somatosensory associative fear memory. The animal was moved back to the home cage after 2 min of rest. Based on the previously reported critical window for spindle-associated memory consolidation as 2–6 h after acquisition^32,43,44^, we stimulated the OG and Sham animals at ~3 h after memory acquisition. Specifically, when animals had rest for ~3 h in the consolidation phase and were observed to be asleep (with eyes closed and curled-up body posture)^45,46^ or immobile/quiet resting (i.e., quiescent state) for a ~30 min period, periodic 8 Hz 24-pulse stimulations were delivered once every 30 s for 40 min. The behavioral state of the animal during stimulation was comparable to the drowsy, asleep and awake quiescent states in previous literature whereby spontaneous spindle activities were detected^6–9^. 8 Hz 24-pulse stimulation paradigm was used as it is the most effective paradigm in evoking brain-wide spindle-like activities. Both the light flash and optogenetic stimulation were controlled by the same system used in electrophysiology experiments. Twenty-four hours following memory acquisition, all groups underwent the extinction phase. Animals were placed in a white chamber with flat metal floor, a different context to the one received during the acquisition phase. After 2 min of exploration, they received 45 periodic repetitions of 10 s 5 Hz light flash (at intervals of 10 s) in the absence of foot shock. After each acquisition or extinction phase, the chambers were cleaned with 70% ethanol and allowed to fully dry to minimize olfactory cues.

To assess the memory performance, freezing levels during each CS presentation were reported for memory acquisition and extinction. Freezing rates were calculated as relative duration (percentage) of freezing during periods of CS presentations using an automatic weight transducer system (Packwin v2.0 and StartFear System, Harvard Apparatus, Holliston, Massachusetts, USA). Animals that showed over-generalized fear (freezing rate during exploration > 40%, two Naïve-Normal, two OG-Normal and two Sham-Normal animals) and failure of memory acquisition (freezing rate during the presentation of the final CS-US pair <50%, one Sham-Normal, one Sham-Aging and two OG-Normal animals) were considered as outliers and were excluded from further experiments and analyses. One OG-Normal animal that did not stay at quiescent or sleep states (i.e., with eyes closed and curled-up body posture) during stimulation was excluded from further experiments. Comparisons of freezing levels during acquisition were done by performing mixed-design two-way ANOVA with post-hoc Bonferroni-corrected *t*-tests to ensure successful memory acquisitions among all groups. Freezing levels during memory extinction were first summarized into 9 sets, whereby each is an average of 5 periodic CS, and were then tested by mixed-design two-way ANOVA with post-hoc Bonferroni-corrected *t*-tests.

For animals that underwent vfMRI experiments, baseline vfMRI sessions (PRE) were acquired one day before the habituation phase while the other vfMRI sessions (POST) were acquired during the same phase as memory extinction in the pure behavioral experiments. The vfMRI animals underwent the same habituation, acquisition, and consolidation phases as the animals in the pure behavioral experiments. Freezing levels during each CS presentation were reported for memory acquisition. Animals met the same outliner exclusion criteria as in the pure behavioral experiments or displayed weak baseline vfMRI activations were excluded from further analysis. Comparisons of freezing levels during acquisition were done by two-way ANOVA with post-hoc Bonferroni-corrected *t*-tests to ensure successful memory acquisitions in vfMRI animals and to validate that any between-conditions differences (i.e., POST vs. PRE) in the vfMRI results were learning/memory-dependent.

### Accelerated aging animal model induction protocol

To examine the effects of optogenetically-evoked brain-wide spindle-like activities in aging animals, VPM optogenetically transfected animals were treated with daily injection of D-galactose (50 mg/kg, subcutaneous) for 8 weeks^91^ before chronic fiber implantation, behavioral and fMRI experiments.

### Histology, immunohistochemistry, and confocal imaging

To confirm the specific expression of ChR2-mCherry in the VPM/MD excitatory neurons, histology was performed according to the previously published procedure^38,39^. Upon completion of experiments, a selected number of optogenetically transfected animals (*n* = 3 for VPM; *n* = 2 for MD) were anesthetized with pentobarbital and then transcardially perfused with ice-cold 4% paraformaldehyde (PFA) in phosphate buffered saline (PBS). The brains were equilibrated in 20% sucrose in PBS at 4 °C overnight. Axial sections (40 μm) were prepared on a freezing microtome (model 860, AO Scientific Instruments). Consecutive sections were mounted and examined with a laser confocal microscope (Carl Zeiss LSM780). For immunohistochemistry, free-floating sections were processed with 5% normal goat serum and 0.3% Triton X-100 in PBS with primary antibodies against rabbit polyclonal to CaMKIIα (1:400; Abcam) at 4 °C for 24 h. After washing with PBS, sections were then incubated for 2 h at room temperature with secondary antibodies Alexa Fluor 647 conjugate goat anti-rabbit IgG and Alexa Fluor 488 conjugate goat anti-guinea pig IgG (both 1:500; Molecular Probe). Slices were then washed and mounted using FluoroShield mounting medium with DAPI (Abcam). Double or triple immunofluorescence was assessed with a laser confocal microscope (Carl Zeiss LSM780).

### Reporting summary

Further information on research design is available in the Nature Portfolio Reporting Summary linked to this article.

## Supplementary information

Supplementary Information

Reporting Summary

## Data Availability

The fMRI, electrophysiology and behavioral data generated in in this study are under active use by the reporting laboratory; all the raw data that support the findings of this study are available from the corresponding author upon request. Source data for line graphs and scatter plots of evoked BOLD fMRI signals, electrophysiological traces and behavioral results in the main figures are provided with this paper in the Source Data file. Source data are provided with this paper.

## Source data

Source Data

## Code availability

The MATLAB (MathWorks, USA) was used to process the fMRI, electrophysiology, and behavioral data. The SPM12 fMRI toolbox (Wellcome Department of Imaging Neuroscience, University College London, UK) was used to process the fMRI data. The EEGlab toolbox (Swartz Center for Computational Neuroscience, University of California San Diego, USA) was used to process the electrophysiological data.
