# Supplementary material for: Functional MRI reveals brain-wide actions of thalamically-initiated oscillatory activities on associative memory consolidation
Source: Nat Commun. 2023 Apr 17;14:2195. doi: 10.1038/s41467-023-37682-8 (PMC10110623; doi:10.1038/s41467-023-37682-8)
Supplement: Supplementary file 1 — Supplementary Information [file 41467_2023_37682_MOESM1_ESM.pdf]

Supplementary Information (SI Figures)

Functional MRI Reveals Brain-wide Actions of Thalamically-Initiated Oscillatory Activities on Associative Memory Consolidation

Xunda Wang[1,2*], Alex T. L. Leong[1,2*], Shawn Z. K. Tan[3], Eddie C. Wong[1,2], Yilong Liu[1,2], Lee-Wei Lim[3], Ed X. Wu[1,2,3†]

[1]Laboratory of Biomedical Imaging and Signal Processing, The University of Hong Kong, Pokfulam, Hong Kong SAR, China

[2]Department of Electrical and Electronic Engineering, The University of Hong Kong, Pokfulam, Hong Kong SAR, China

[3]School of Biomedical Sciences, Li Ka Shing Faculty of Medicine, The University of Hong Kong, Pokfulam, Hong Kong SAR, China

[*]These authors contributed equally to this work.

[†]Correspondence should be addressed to Ed X. Wu, Ph.D.:

Laboratory of Biomedical Imaging and Signal Processing, Department of Electrical and Electronic Engineering, School of Biomedical Sciences, The University of Hong Kong, Pokfulam, Hong Kong, Hong Kong SAR, China.

Fax: +852-2859-8738.

Tel: +852-3917-7096.

Email: ewu@eee.hku.hk

Keywords: fMRI, optogenetic, thalamo-cortical, spindle activities, cross-modal, spatiotemporal characteristics, memory consolidation

SI Figures

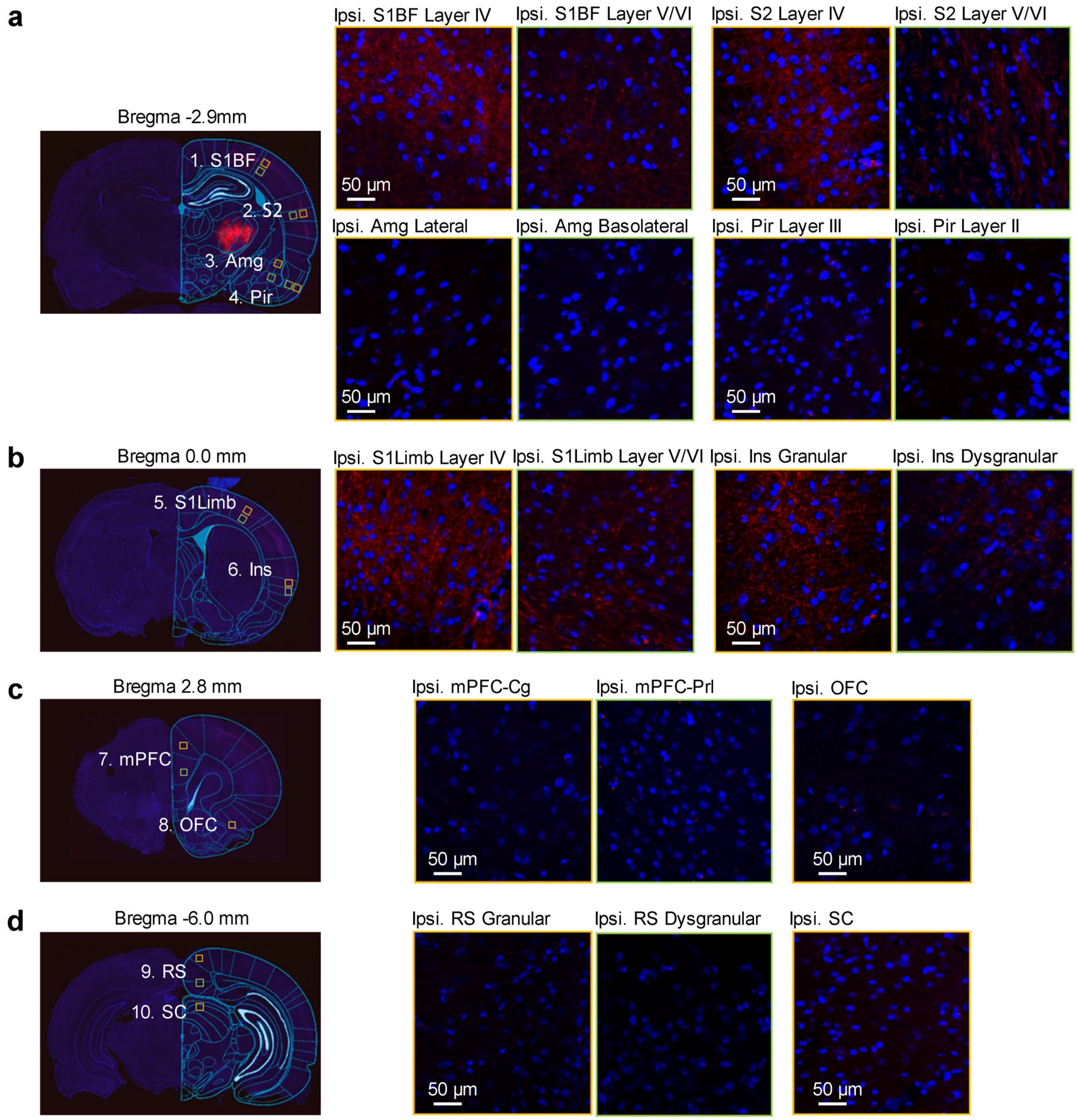

Figure S1. Histological characterization of ChR2::CaMKIIα viral expression shows transfection only in projection terminals at ventral posteromedial (VPM) projection targets (S1 and S2), not at other remote regions (i.e., Amg, Pir, Ins, mPFC, OFC, RS and SC). VPM thalamo-cortical projections synapse in **a** 1. layer IV and V/VI of S1BF and 2. layer IV and V/VI

of S2, indicated by no colocalization between mCherry and DAPI. Confocal images of ChR2-mCherry expression in 3. lateral and basolateral nuclei of the amygdala (Amg), 4. layer II and III of piriform cortex (Pir); **b** 5. layer IV and V/VI of S1Limb, 6. granular and dysgranular insular areas; **c** 7. cingulate cortex (Cg) and prelimbic cortex (Prl) in the medial prefrontal cortex (mPFC), 8. OFC; **d** 9. granular and dysgranular retrosplenial areas, 10. superior colliculus (SC) also show no colocalization between mCherry and DAPI (S1Limb and Ins) or show no visible expression (Amg, Pir, mPFC, OFC, RS and SC); lower-magnification (left) and higher-magnification (right).

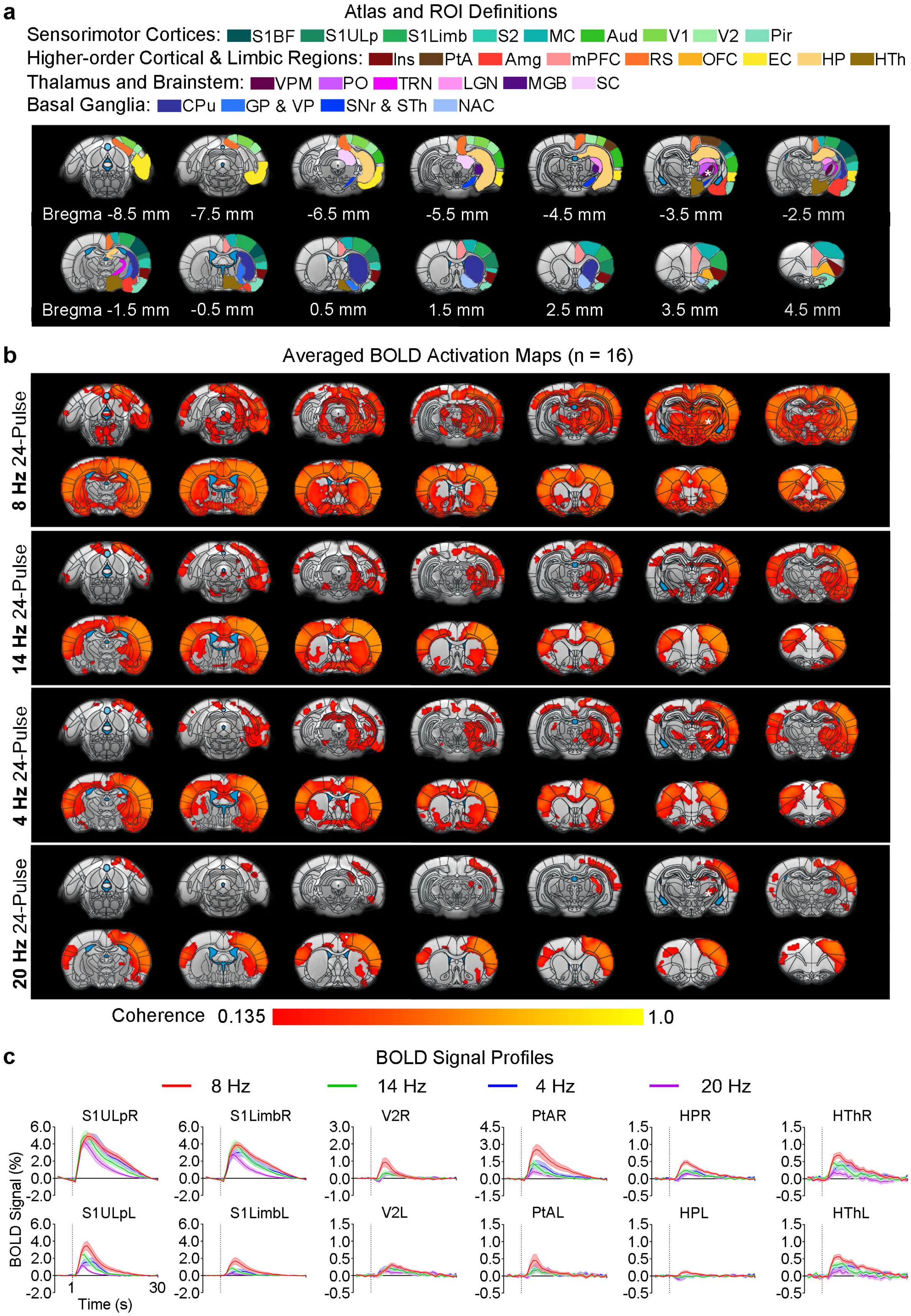
a
Atlas and ROI Definitions
Sensorimotor Cortices: S1BF S1ULp S1Limb S2 MC Aud V1 V2 Pir
Higher-order Cortical & Limbic Regions: Ins PtA Amg mPFC RS OFC EC HP HTh
Thalamus and Brainstem: VPM PO TRN LGN MGB SC
Basal Ganglia: CPu GP & VP SNr & STh NAC
Bregma -8.5 mm
-7.5 mm
-6.5 mm
-5.5 mm
-4.5 mm
-3.5 mm
-2.5 mm
Bregma -1.5 mm
-0.5 mm
0.5 mm
1.5 mm
2.5 mm
3.5 mm
4.5 mm
b
Averaged BOLD Activation Maps (n = 16)
8 Hz 24-Pulse
14 Hz 24-Pulse
4 Hz 24-Pulse
20 Hz 24-Pulse
Coherence 0.135
1.0
c
BOLD Signal Profiles
8 Hz
14 Hz
4 Hz
20 Hz
S1ULpR
S1LimbR
V2R
PtAR
HPR
HThR
S1ULpL
S1LimbL
V2L
PtAL
HPL
HThL
BOLD Signal (%)
Time (s)
1
30
6.0
4.0
2.0
0.0
-2.0
3.0
1.0
-1.0
4.5
1.5
-1.5
0.5
-0.5

Figure S2. BOLD activations in other sensory cortices, higher order cortical and limbic regions upon 24-pulse stimulations across typical spindle frequencies reveal brain-wide cross-modal targets and their frequency-dependent response property. a Illustration of atlas-based ROI definitions in the sensorimotor cortices, higher-order cortical and limbic regions, thalamus and brainstem, and basal ganglia (asterisk, stimulation site). **b** Averaged BOLD activation maps for 24-pulse optogenetic stimulations at different frequencies: 8, 14, 4, 20 Hz ($n = 16$; asterisk, stimulation site; two-tailed coherence tests, coherence of 0.135 corresponds to $P < 0.001$, followed by two-tailed one-sample group level *t*-tests, threshold-free cluster enhancement with family wise error rate, TFCE-FWE, corrected $P < 0.05$; see Bonferroni-corrected $P < 0.05$ in **Figure S3**). **c** BOLD signal profiles extracted from other atlas-based sensorimotor cortical and non-sensorimotor limbic ROIs defined in (**a**) (error bar indicates ± s.e.m.).

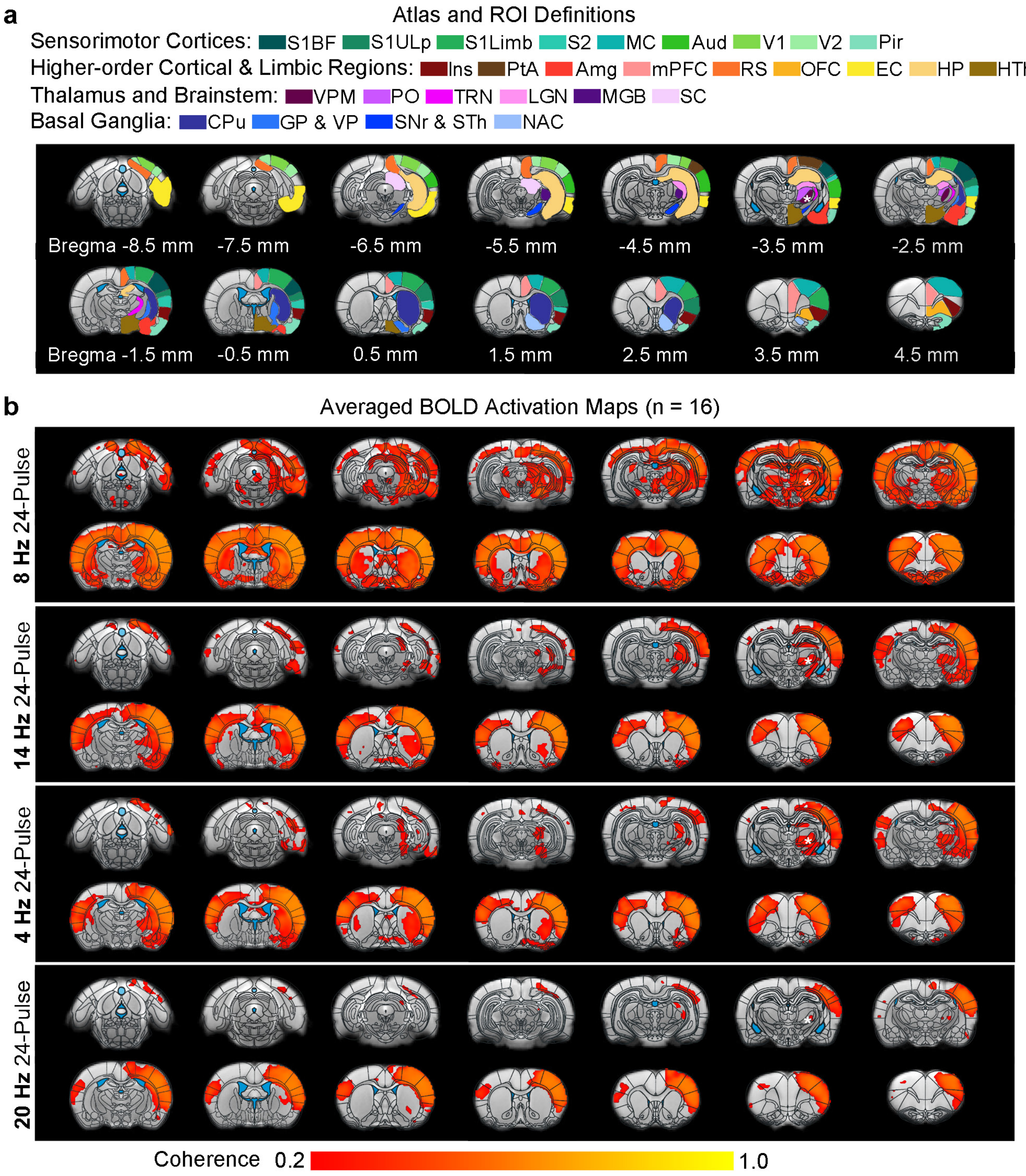

Figure S3. BOLD activations from 24-pulse stimulations across typical spindle frequencies after voxel-based Bonferroni multiple comparisons correction confirmed brain-wide cross-modal targets and their respective frequency-dependent response property. a Illustration of atlas-based ROI definitions in the sensorimotor cortices, higher-order cortical and limbic regions,

thalamus and brainstem, and basal ganglia (asterisk, stimulation site). **b** Averaged BOLD activation maps for 24-pulse optogenetic stimulations at different frequencies: 8, 14, 4, 20 Hz (n = 16; asterisk, stimulation site; two-tailed coherence tests, coherence of 0.2 corresponds to Bonferroni-corrected $P < 0.05$).

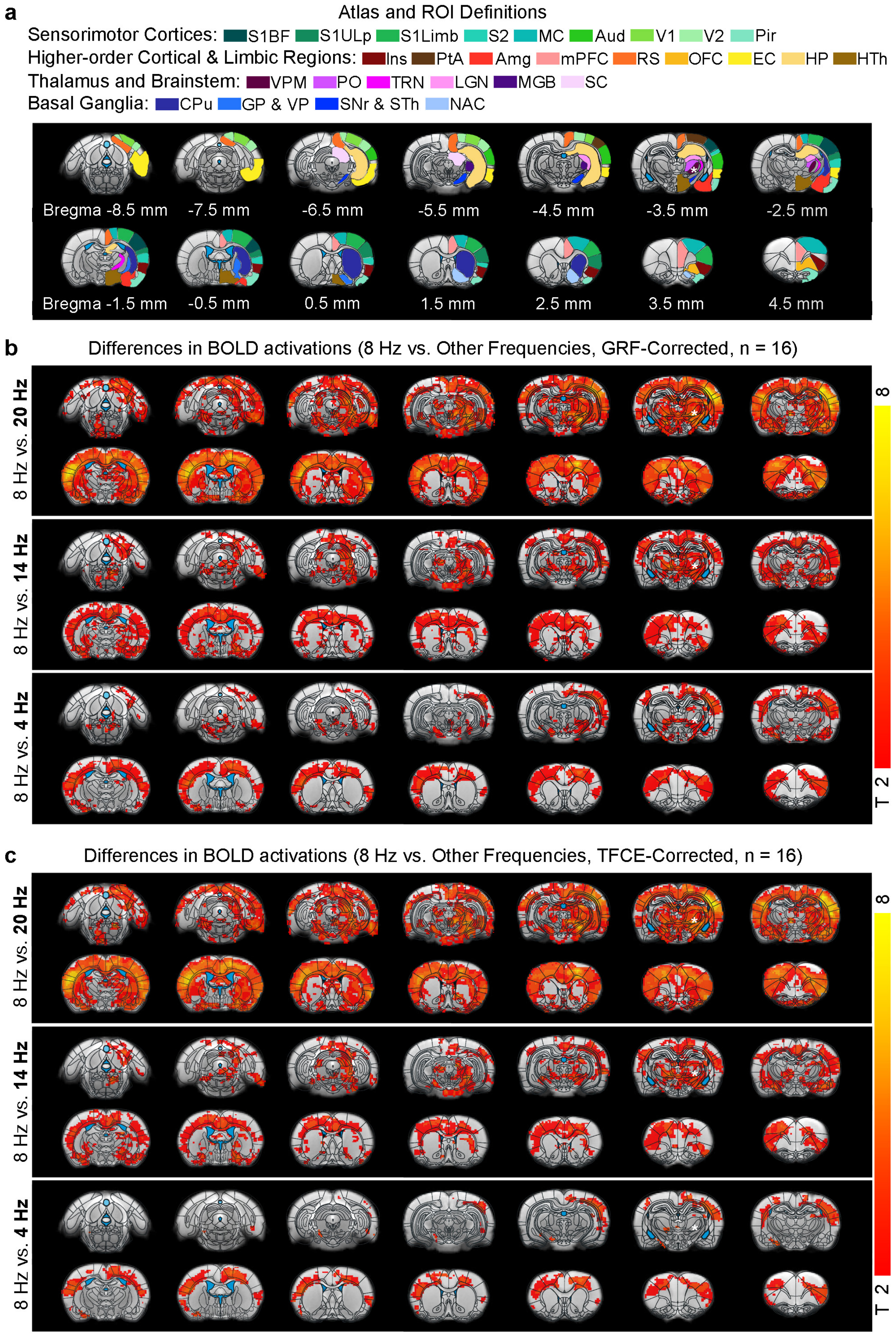

a
Atlas and ROI Definitions
Sensorimotor Cortices: S1BF S1ULp S1Limb S2 MC Aud V1 V2 Pir
Higher-order Cortical & Limbic Regions: Ins PtA Amg mPFC RS OFC EC HP HTh
Thalamus and Brainstem: VPM PO TRN LGN MGB SC
Basal Ganglia: CPu GP & VP SNr & STh NAC
Bregma -8.5 mm
-7.5 mm
-6.5 mm
-5.5 mm
-4.5 mm
-3.5 mm
-2.5 mm
Bregma -1.5 mm
-0.5 mm
0.5 mm
1.5 mm
2.5 mm
3.5 mm
4.5 mm
b
Differences in BOLD activations (8 Hz vs. Other Frequencies, GRF-Corrected, n = 16)
8 Hz vs. 20 Hz
8 Hz vs. 14 Hz
8 Hz vs. 4 Hz
T 2
8
c
Differences in BOLD activations (8 Hz vs. Other Frequencies, TFCE-Corrected, n = 16)
8 Hz vs. 20 Hz
8 Hz vs. 14 Hz
8 Hz vs. 4 Hz
T 2
8

61

Figure S4. Statistical comparison of BOLD activations between 8 Hz and other frequencies of 24-pulse stimulations with cluster-based multiple comparisons correction based on Gaussian random field (GRF) and TFCE-FWE approaches. a Illustration of atlas-based ROI definitions in the sensorimotor cortices, higher-order cortical and limbic regions, thalamus and brainstem, and basal ganglia (asterisk, stimulation site). **b** Significant differences in BOLD activations between 8 Hz and other frequencies (20, 14, and 4 Hz) of 24-pulse stimulations after GRF correction (n = 16; asterisk, stimulation site; two-tailed two-sample *t*-tests, voxel level $P < 0.05$ and cluster level $P < 0.001$). **c** Significant differences in BOLD activations between 8 Hz and other frequencies (20, 14, and 4 Hz) of 24-pulse stimulations after TFCE-FWE correction (n = 16; asterisk, stimulation site; two-tailed two-sample *t*-tests, corrected $P < 0.05$).

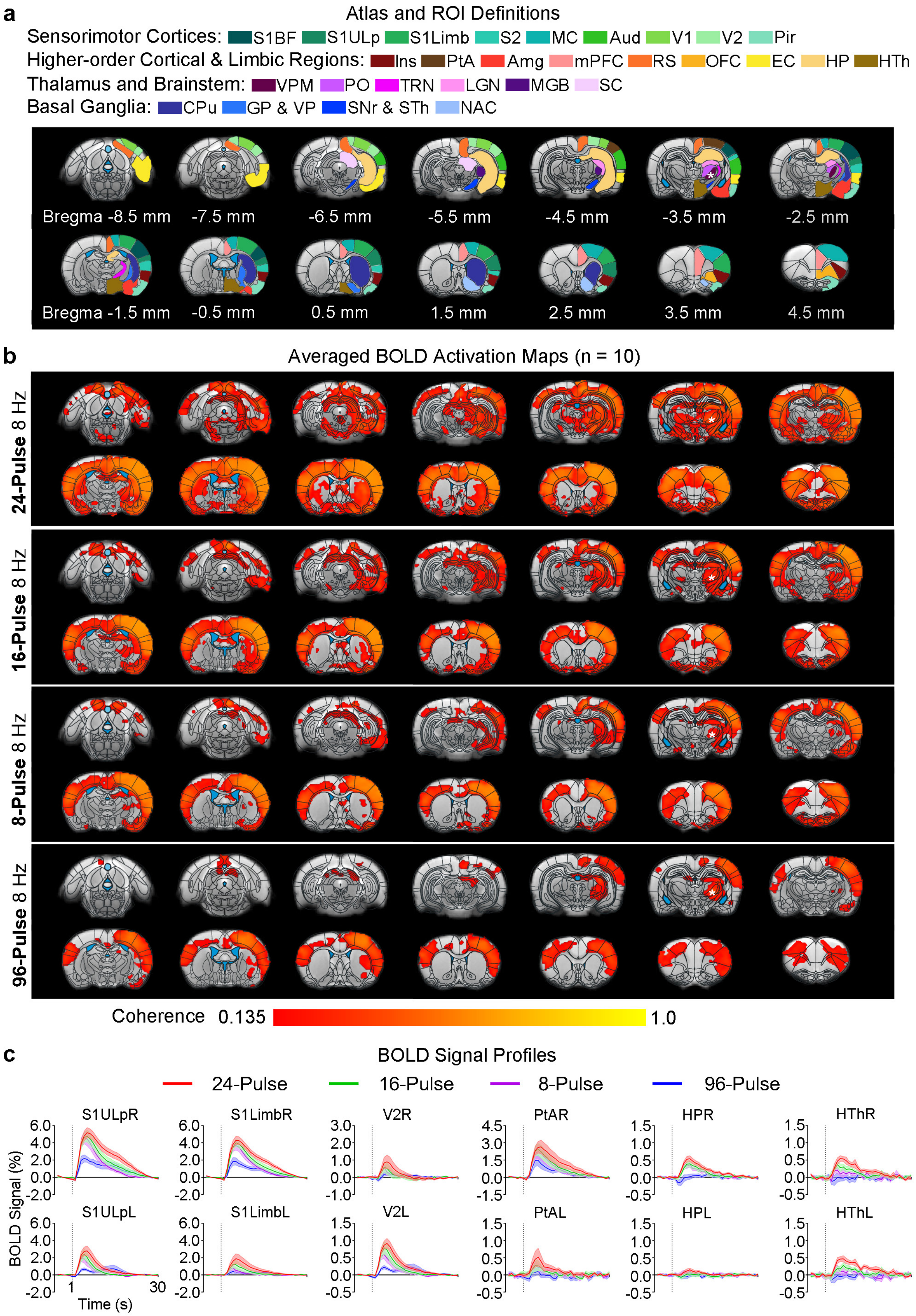

a
Atlas and ROI Definitions
Sensorimotor Cortices: S1BF S1ULp S1Limb S2 MC Aud V1 V2 Pir
Higher-order Cortical & Limbic Regions: Ins PtA Amg mPFC RS OFC EC HP HTh
Thalamus and Brainstem: VPM PO TRN LGN MGB SC
Basal Ganglia: CPu GP & VP SNr & STh NAC
Bregma -8.5 mm
-7.5 mm
-6.5 mm
-5.5 mm
-4.5 mm
-3.5 mm
-2.5 mm
Bregma -1.5 mm
-0.5 mm
0.5 mm
1.5 mm
2.5 mm
3.5 mm
4.5 mm
b
Averaged BOLD Activation Maps (n = 10)
24-Pulse 8 Hz
16-Pulse 8 Hz
8-Pulse 8 Hz
96-Pulse 8 Hz
Coherence 0.135
1.0
c
BOLD Signal Profiles
24-Pulse
16-Pulse
8-Pulse
96-Pulse
S1ULpR
S1LimbR
V2R
PtAR
HPR
HThR
S1ULpL
S1LimbL
V2L
PtAL
HPL
HThL
BOLD Signal (%)
Time (s)

Figure S5. BOLD activations in other sensory cortices, higher order cortical and limbic regions reveal the length-dependent response property for the brain-wide cross-modal targets of somatosensory thalamically-evoked activities upon 8 Hz stimulations. a Illustration of atlas-based ROI definitions in the sensorimotor cortices, higher-order cortical and limbic regions, thalamus and brainstem, and basal ganglia (asterisk, stimulation site). **b** Averaged BOLD activation maps for 8 Hz optogenetic stimulations at different lengths: 8-, 16-, 24-, 96-pulse (n = 10; asterisk, stimulation site; two-tailed coherence tests, coherence of 0.135 corresponds to $P < 0.001$, followed by two-tailed one-sample group level t-tests, TFCE-FWE corrected $P < 0.05$; see Bonferroni-corrected $P < 0.05$ in **Figure S6**). **c** BOLD signal profiles extracted from atlas-based sensorimotor cortical and non-sensorimotor limbic ROIs defined in (**a**) (error bar indicates ± s.e.m.).

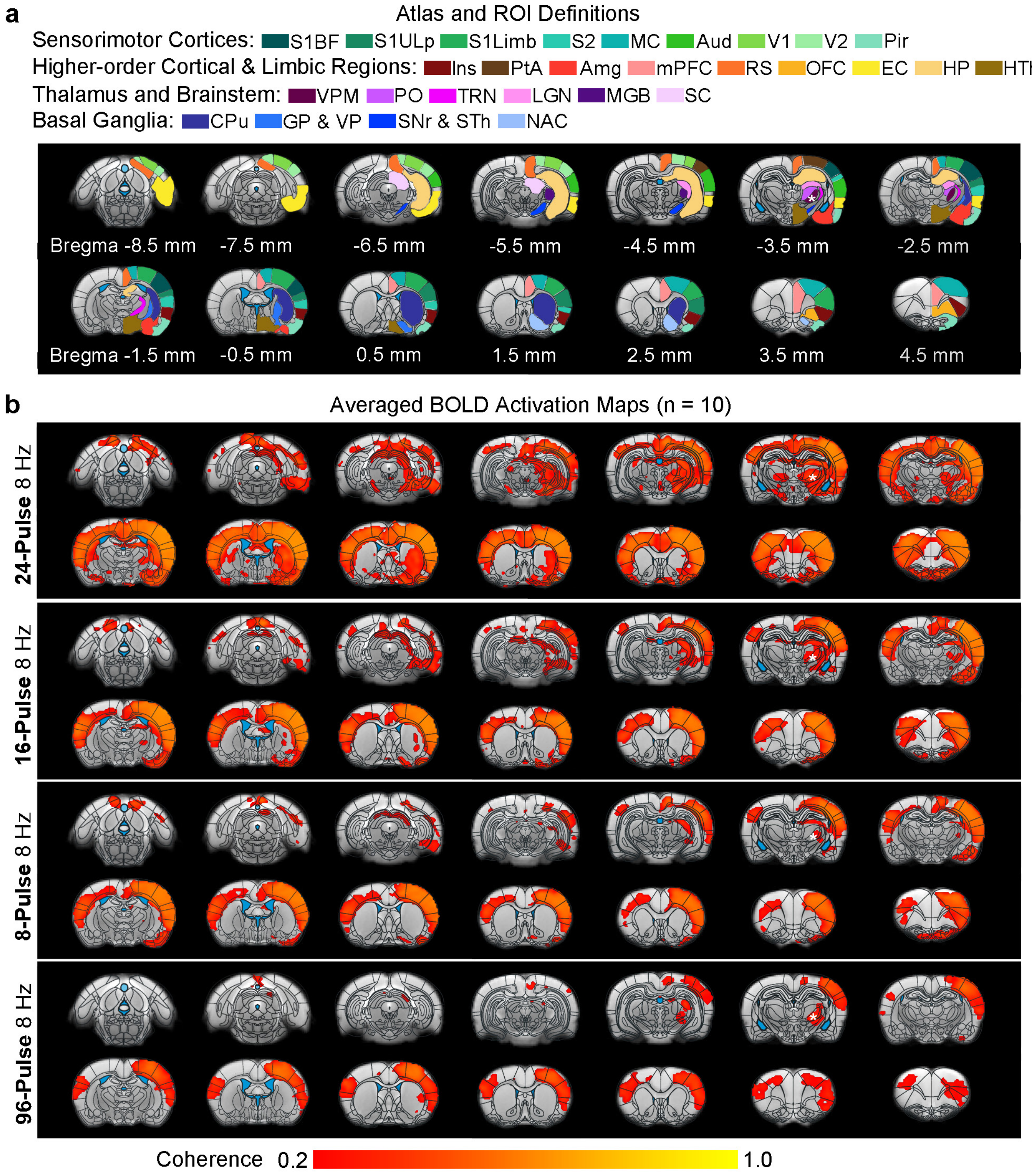

Figure S6. BOLD activations after Bonferroni correction confirmed length-dependent response properties for brain-wide cross-modal targets of somatosensory thalamically-evoked activities upon 8 Hz stimulation. a Illustration of atlas-based ROI definitions in the sensorimotor cortices, higher-order cortical and limbic regions, thalamus and brainstem, and basal

ganglia (asterisk, stimulation site). **b** Averaged BOLD activation maps for 8 Hz optogenetic stimulations at different lengths: 8-, 16-, 24-, and 96-pulse (n = 10; asterisk, stimulation site; two-tailed coherence tests, coherence of 0.2 corresponds to Bonferroni-corrected $P < 0.05$).

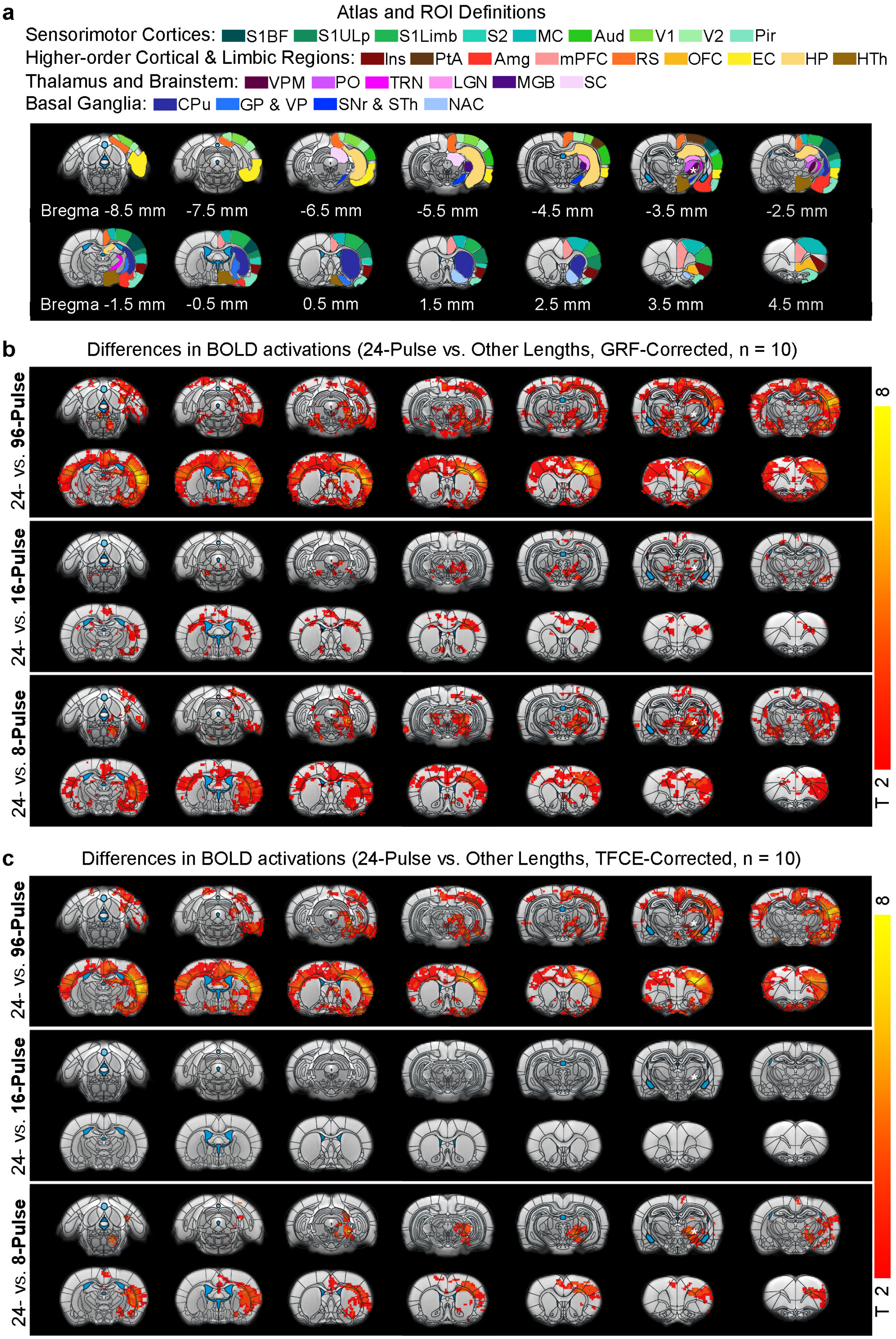
a
Atlas and ROI Definitions
Sensorimotor Cortices: S1BF S1ULp S1Limb S2 MC Aud V1 V2 Pir
Higher-order Cortical & Limbic Regions: Ins PtA Amg mPFC RS OFC EC HP HTh
Thalamus and Brainstem: VPM PO TRN LGN MGB SC
Basal Ganglia: CPu GP & VP SNr & STh NAC
Bregma -8.5 mm
-7.5 mm
-6.5 mm
-5.5 mm
-4.5 mm
-3.5 mm
-2.5 mm
Bregma -1.5 mm
-0.5 mm
0.5 mm
1.5 mm
2.5 mm
3.5 mm
4.5 mm
b
Differences in BOLD activations (24-Pulse vs. Other Lengths, GRF-Corrected, n = 10)
24- vs. 96-Pulse
24- vs. 16-Pulse
24- vs. 8-Pulse
8
T 2
c
Differences in BOLD activations (24-Pulse vs. Other Lengths, TFCE-Corrected, n = 10)
24- vs. 96-Pulse
24- vs. 16-Pulse
24- vs. 8-Pulse
8
T 2

Figure S7. Statistical comparison of BOLD activations between 24-pulse and other lengths of 8 Hz stimulations with multiple comparisons correction based on GRF and TFCE-FWE approaches. a Illustration of atlas-based ROI definitions in the sensorimotor cortices, higher-order cortical and limbic regions, thalamus and brainstem, and basal ganglia (asterisk, stimulation site). **b** Significant differences in BOLD activations between 24-pulse and other lengths (96-, 16-, and 8-pulse) of 8 Hz stimulations after GRF cluster-level correction (n = 10; asterisk, stimulation site; two-tailed two-sample *t*-tests, voxel level $P < 0.05$ and cluster level $P < 0.001$). **c** Significant differences in BOLD activations between 24-pulse and other lengths (96-, 16-, and 8-pulse) of 8 Hz stimulations after TFCE-FWE correction (n = 16; asterisk, stimulation site; two-tailed two-sample *t*-tests, corrected $P < 0.05$).

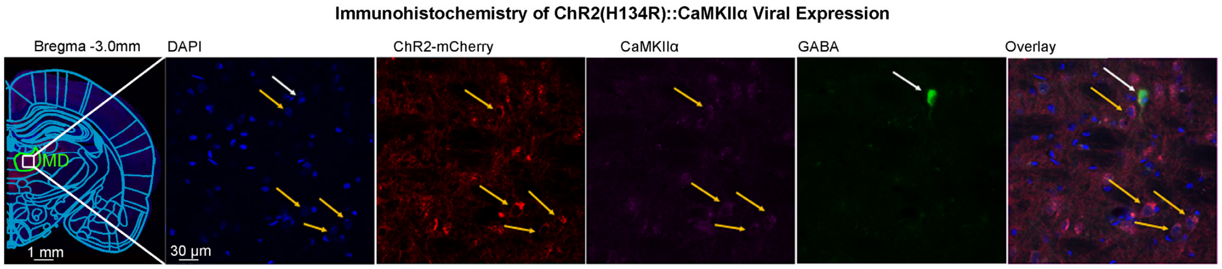

Figure S8. Histological characterization of ChR2::CaMKIIα viral expression in MD thalamocortical excitatory neurons. Confocal images of ChR2-mCherry expression in MD with lower (Left) and higher (Right) magnification. Overlay of images co-stained for the nuclear marker DAPI, excitatory marker CaMKIIα, inhibitory marker GABA, and mCherry revealed colocalization of mCherry and CaMKIIα in the cell body of MD excitatory thalamo-cortical neurons (indicated by yellow arrows), not GABAergic inhibitory neurons (indicated by white arrows).

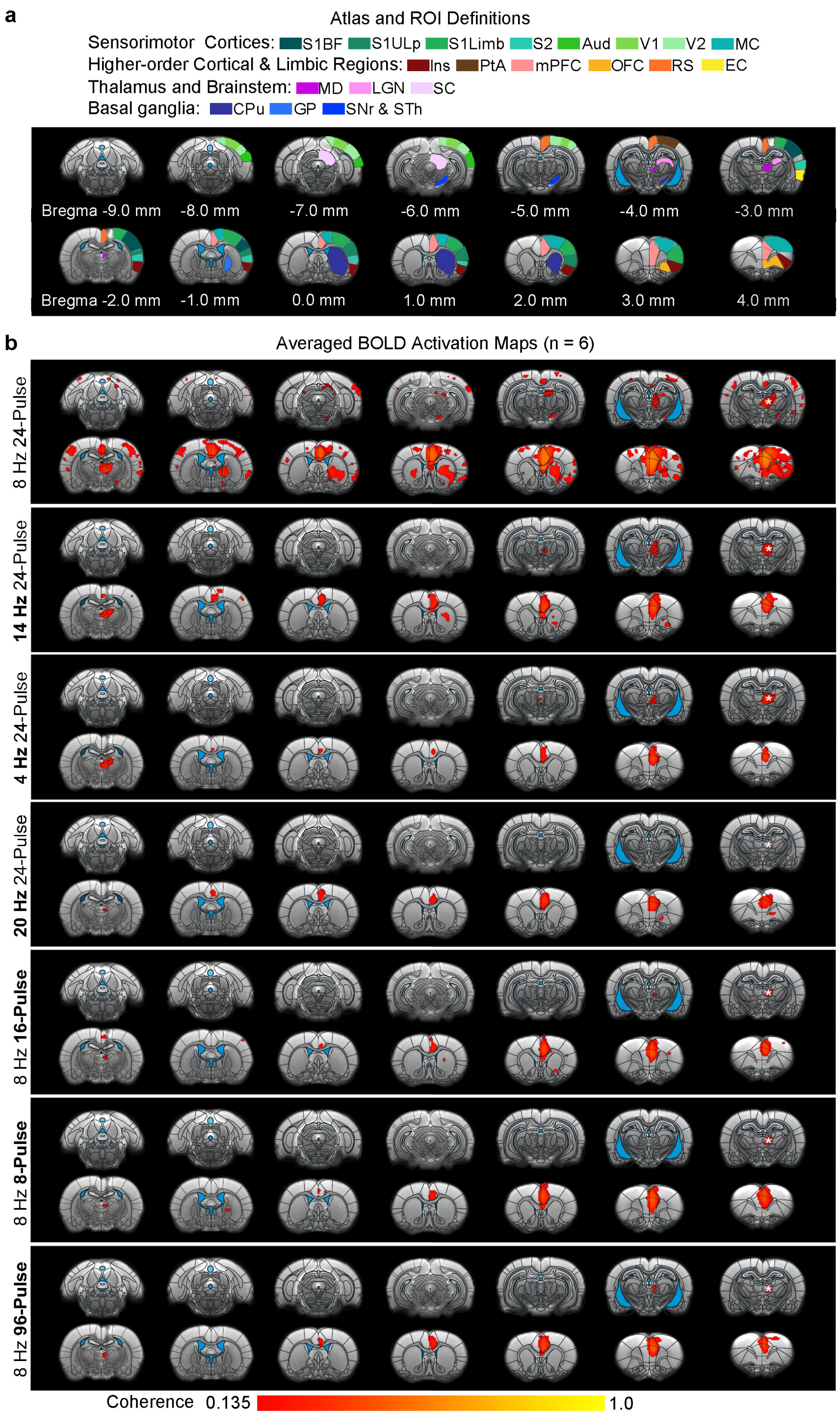

Figure S9. Brain-wide BOLD activations upon optogenetic stimulation of medial dorsal thalamus (MD) show similar frequency- & length-dependent cross-modal recruitment properties as those evoked by VPM stimulations. a Illustration of atlas-based ROI definitions

for the activated regions (asterisk, stimulation site). **b** Averaged BOLD activation maps for 24-pulse optogenetic stimulations at different frequencies (i.e., 8, 14, 4, 20 Hz) or 8 Hz stimulations with different pulse lengths (i.e., 8-, 16-, 24-, 96-pulse) ($n = 6$; asterisk, stimulation site; two-tailed coherence tests, coherence of 0.135 corresponds to $P < 0.001$, followed by two-tailed one-sample group level *t*-tests, TFCE-FWE corrected $P < 0.05$). As expected, the strongest activations were located at the limbic thalamo-cortical circuit (MD and frontal cortices—the primary projection targets of MD. Importantly, 8 Hz 24-pulse stimulation evoked the most widespread and robust brain-wide BOLD activations across limbic (MD, mPFC, OFC, RS and EC) and sensorimotor (S1BF, S1Limb, S1ULp, S2, Aud, V1, V2, Ins, PtA, MC, LGN, SC, CPu, GP, SNr & STh) regions, while changes of stimulation frequency or length restricted the BOLD activations to limbic regions.

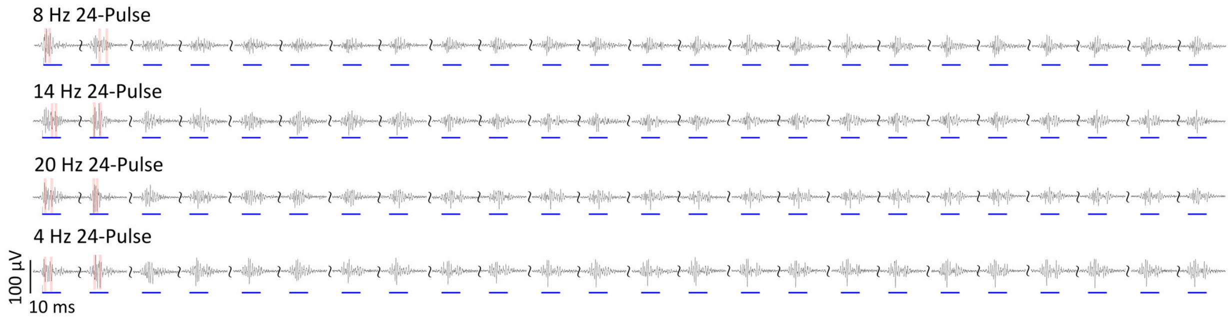

Figure S10. Representative multi-unit activity (MUA) recordings show successfully evoked bursts of neuronal spikes at VPM upon different frequencies of optogenetic stimulations. Note that thalamic burst-like activities as indicated by multiple spikes over the 10 ms stimulation pulse period were evoked at all frequencies. Representative spikes are marked in red for the first two stimulation pulses for each frequency.

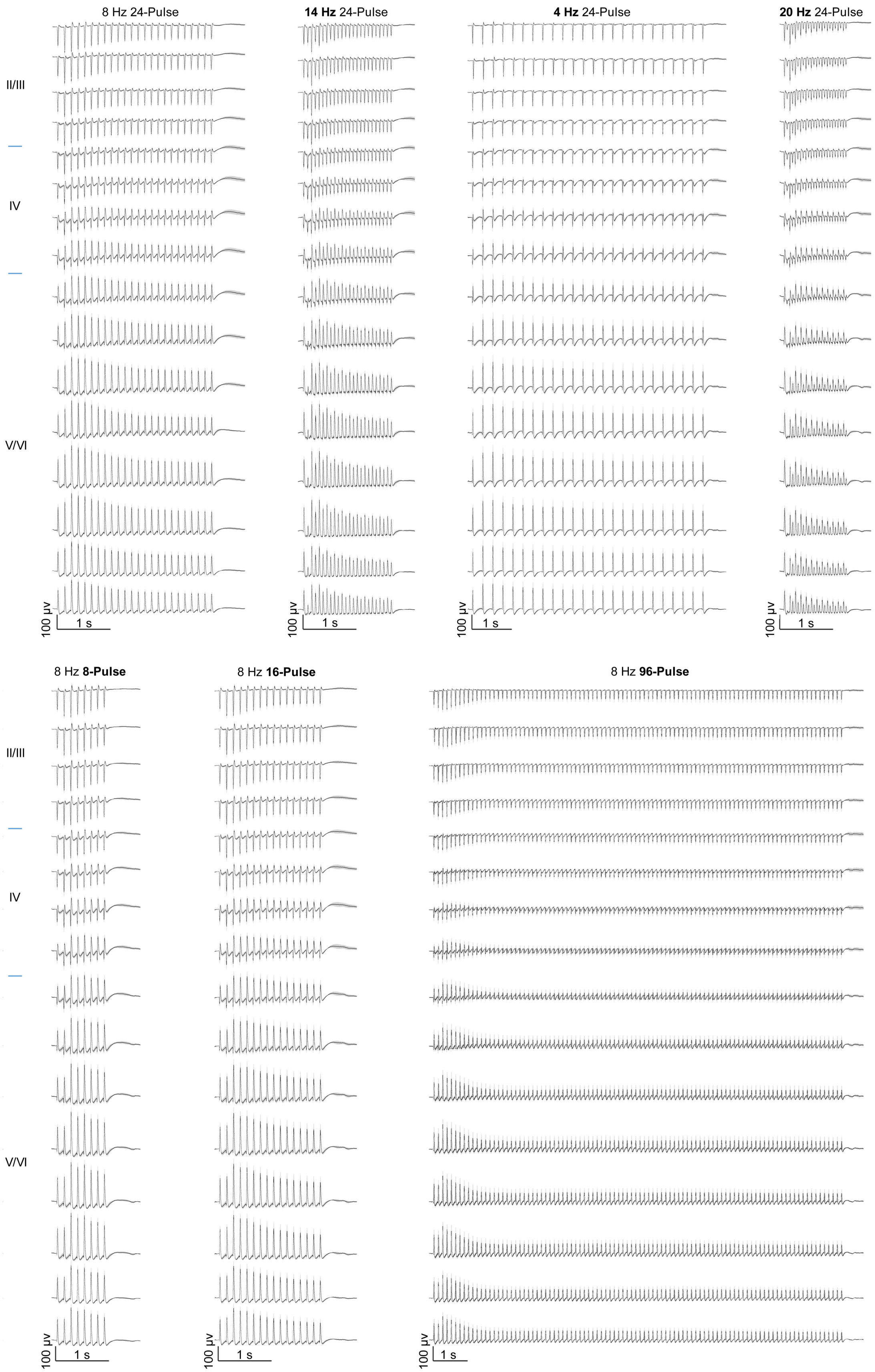

8 Hz 24-Pulse
14 Hz 24-Pulse
4 Hz 24-Pulse
20 Hz 24-Pulse
II/III
IV
V/VI
100 µv
1 s
8 Hz 8-Pulse
8 Hz 16-Pulse
8 Hz 96-Pulse

131

Figure S11. Multi-depth local field potential (LFP) recordings at S1BF reveal somatosensory thalamically-evoked spindle-like activities and confirm their dependence on stimulation frequency and length. Averaged LFPs evoked by optogenetic stimulations at different frequencies and lengths showed that robust spindle-like activities were evoked by the 8 Hz 24-pulse stimulation paradigm (error bar indicates ± s.e.m.). However, with the changes of stimulation frequency to 14, 4, and 20 Hz and or increase of stimulation length to 96-pulse, the evoked LFPs showed decreased responses levels (14 and 20 Hz 24-pulse) or loss of spindle-shaped waveform (layer V/VI responses at 20 Hz 24-pulse and 8 Hz 96-pulse stimulations).

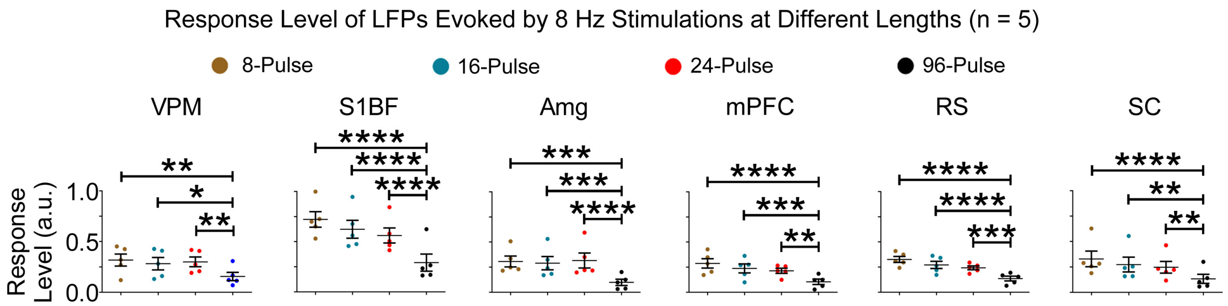

Figure S12. Quantification of multisite LFP response levels reveals the dependence of somatosensory thalamically-evoked brain-wide cross-modal spindle-like neural activities on stimulation length. The prolonged stimulation (96-pulse) that exceeded spindle length (i.e., 3 s or 25 cycles) evoked LFPs with significantly weaker response levels across all regions compared to those at other lengths (8-/16-/24-pulse) (n = 5; error bar indicates ± s.e.m.; one-way ANOVA with Tukey's post hoc test; *, **, *** and **** denote $P < 0.05$, $P < 0.01$, $P < 0.001$ and $P < 0.0001$). Exact P-values are provided in Source Data.

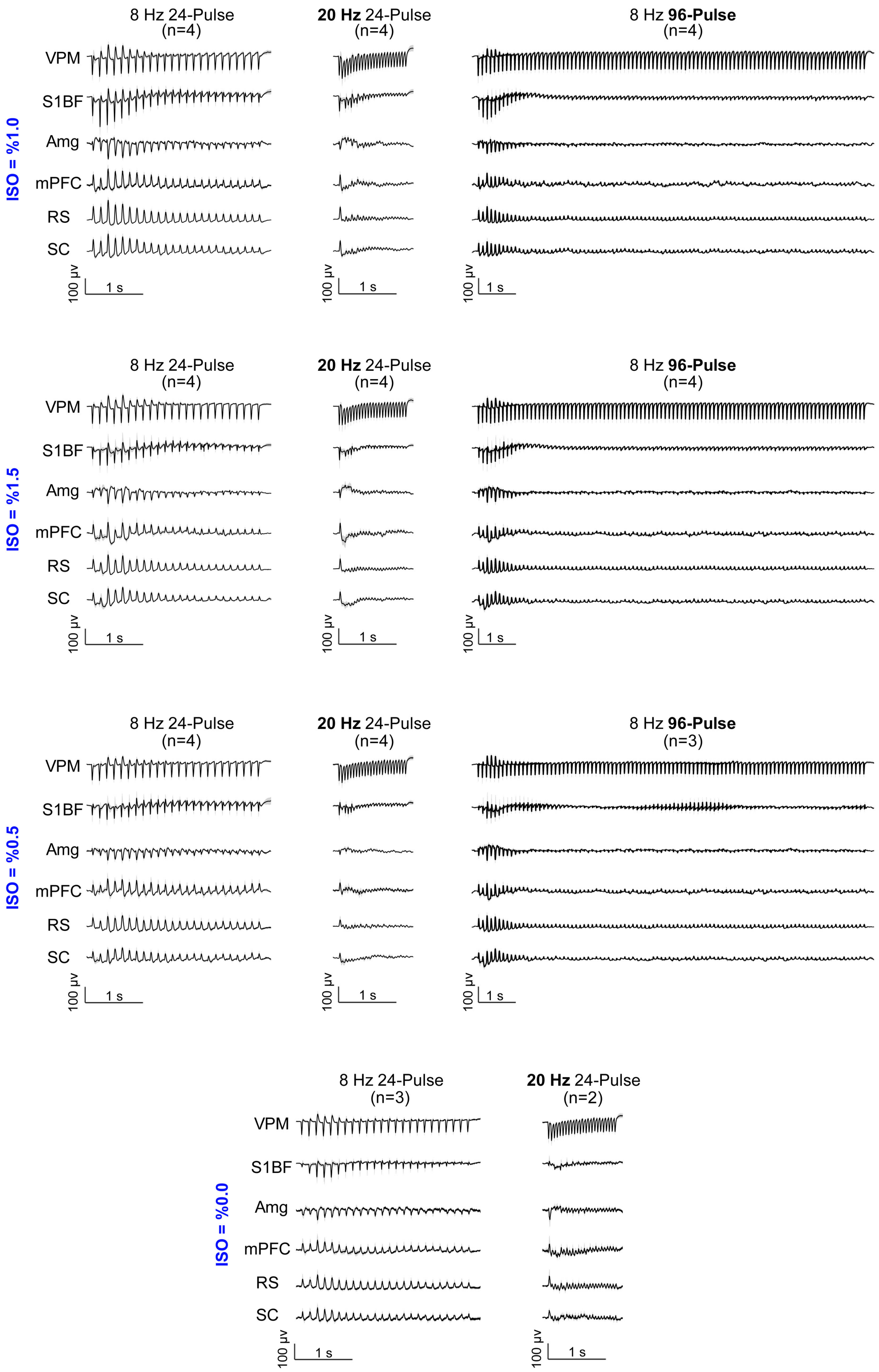
8 Hz 24-Pulse (n=4)
20 Hz 24-Pulse (n=4)
8 Hz 96-Pulse (n=4)
ISO = %1.0
VPM
S1BF
Amg
mPFC
RS
SC
100 μv
1 s
8 Hz 24-Pulse (n=4)
20 Hz 24-Pulse (n=4)
8 Hz 96-Pulse (n=4)
ISO = %1.5
VPM
S1BF
Amg
mPFC
RS
SC
100 μv
1 s
8 Hz 24-Pulse (n=4)
20 Hz 24-Pulse (n=4)
8 Hz 96-Pulse (n=3)
ISO = %0.5
VPM
S1BF
Amg
mPFC
RS
SC
100 μv
1 s
8 Hz 24-Pulse (n=3)
20 Hz 24-Pulse (n=2)
ISO = %0.0
VPM
S1BF
Amg
mPFC
RS
SC
100 μv
1 s

Figure S13. Averaged LFP responses under different light anesthesia levels reveal similar brain-wide propagation properties of evoked spindle-like activities. LFPs at four isoflurane levels (1.0, 1.5, 0.5 and 0.0%) were recorded with the sequence of exposure to different isoflurane levels randomized across animals (error bar indicates ± s.e.m.). Note that recordings were typically made immediately after the cessation of isoflurane (0.0%) before the animal is awake. Altering anesthesia levels caused no apparent changes in the spindle-shaped waveforms of the 8 Hz 24-pulse stimulation evoked LFPs across different recorded locations. Both the increase of stimulation frequency from 8 Hz to 20 Hz or the stimulation length from 24-pulse to 96-pulse resulted in decreased LFP responses across multiple recording locations (i.e., S1BF, Amg, mPFC, RS, and SC).

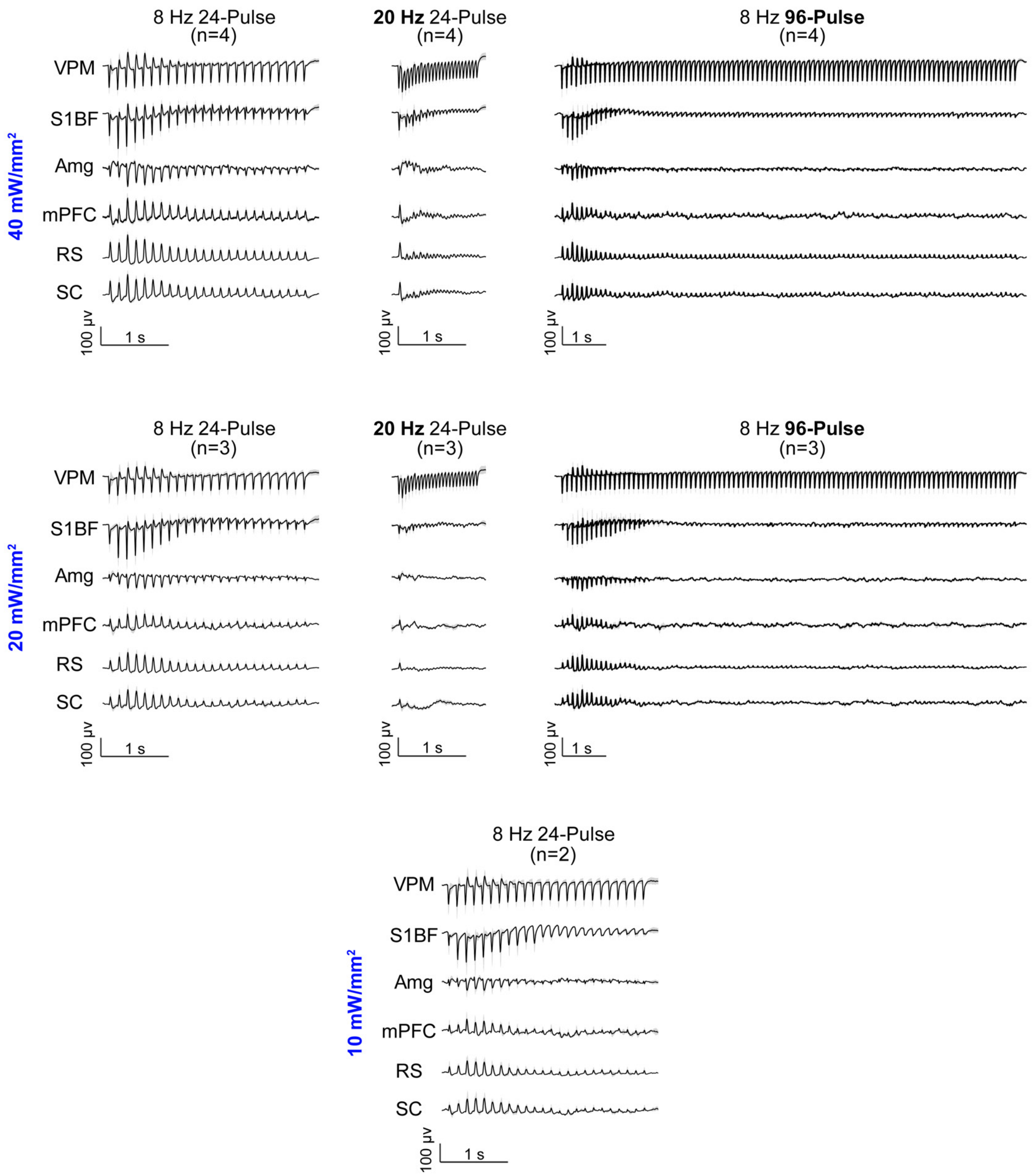

Figure S14. Averaged LFP responses when stimulating at reduced optogenetic light intensities reveal unaffected brain-wide propagation properties of evoked spindle-like activities. Reducing light intensity to 20 mW/mm^2 or 10 mW/mm^2 for the 8 Hz 24-pulse stimulation of VPM caused no apparent change in the spindle-shaped waveforms of the evoked LFPs across different recorded locations (error bar indicates ± s.e.m.). Meanwhile, reducing light intensity also did not affect the response characteristics of evoked LFPs whereby the increase of

stimulation frequency or stimulation length from 8 Hz to 20 Hz or from 24-pulse to 96-pulse, respectively, led to an overall decrease of neural activity propagation and brain-wide LFP responses.

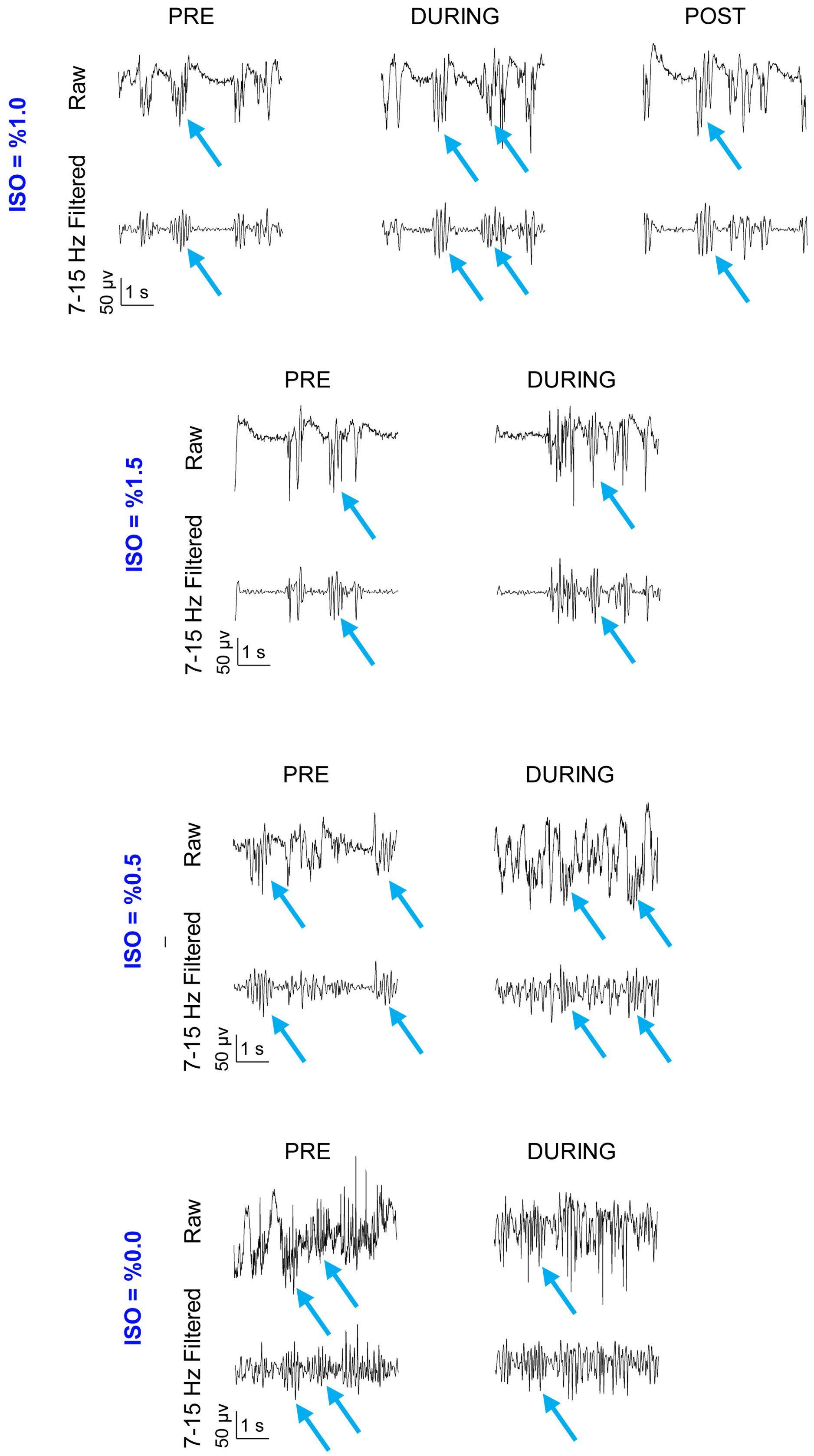
PRE
DURING
POST
ISO = %1.0
Raw
7-15 Hz Filtered
50 µv
1 s
PRE
DURING
ISO = %1.5
Raw
7-15 Hz Filtered
50 µv
1 s
PRE
DURING
ISO = %0.5
Raw
7-15 Hz Filtered
50 µv
1 s
PRE
DURING
ISO = %0.0
Raw
7-15 Hz Filtered
50 µv
1 s

Figure S15. Representative LFPs show the presence of spontaneous spindle activities under different anesthesia levels in S1BF. LFPs at four isoflurane levels (1.0, 1.5, 0.5 and 0.0%) were recorded with the sequence of exposure to different isoflurane levels randomized across animals. Spontaneous spindle activities were detected (indicated by blue arrows) under different anesthesia conditions albeit with varied background neural activities. Their analysis of density, length and amplitude is shown in **Figure S16**. Note that as expected background neural activities are much more prevalent after cessation of isoflurane (0.0%, recorded before animal wake up).

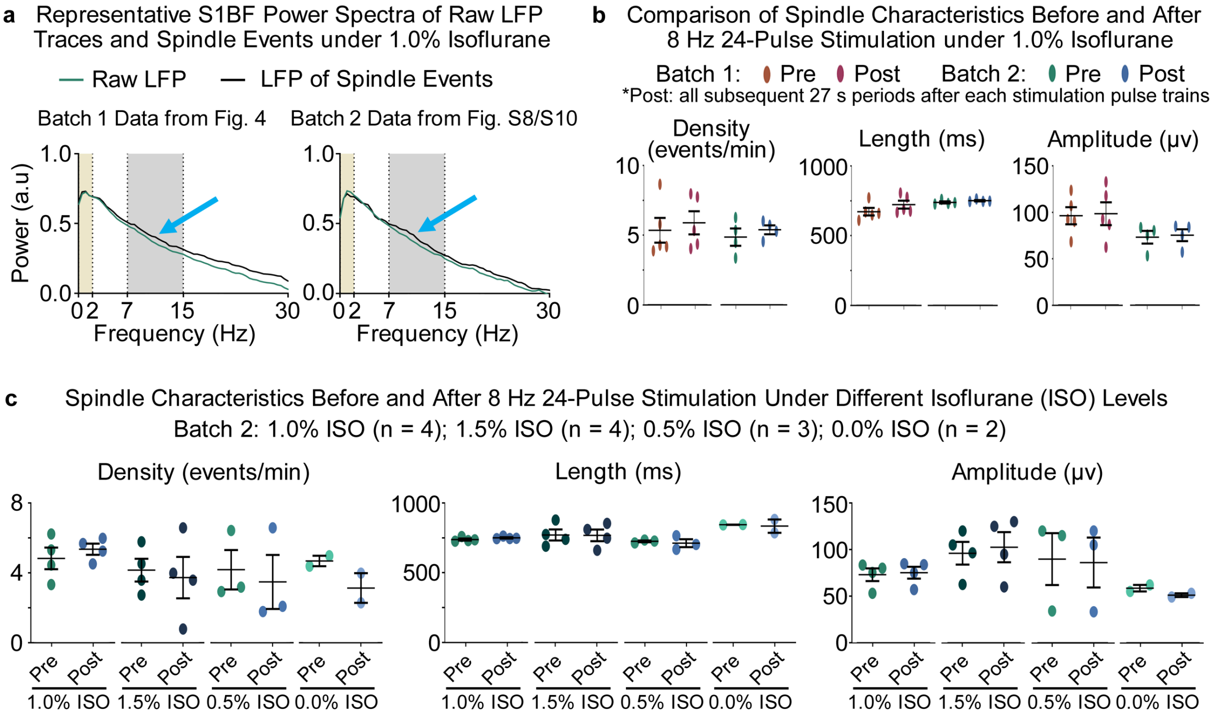

Figure S16. Representative power spectra and characteristics of detected spontaneous spindle activities in LFP data in the 8 Hz 24-pulse stimulation experiments. For batch 1 (data from **Figure 4**, n = 5), LFPs were recorded under 1.0% isoflurane. For batch 2 (data from **Figures S13** and **S15**, n = 5), LFPs were recorded at four isoflurane levels (1.0, 1.5, 0.5 and 0.0%) with the sequence of exposure to different isoflurane levels randomized across animals. **a** Representative power spectra of 30 min LFP traces (green) and all 5 s LFP traces of detected spontaneous spindle events (black) at S1BF under 1.0% isoflurane in the baseline period (prior to any optogenetic stimulation). Distribution of activities in the spindle (7–15 Hz) and slow oscillations (< 2 Hz) frequency ranges were observed. Overall, the spindle activities were more apparent in the spectra of detected spindle events (black line), as indicated by the slight increase in the power at the 7–15 Hz frequency range. **b** Comparisons of spindle density, length, and amplitude in the baseline period (Pre) prior to any optogenetic stimulation and after (Post) 8 Hz 24-pulse optogenetic stimulation across batch 1 and batch 2 LFP data under 1.0% isoflurane anesthesia (error bar indicates ± s.e.m.;

one-way ANOVA with post-hoc Bonferroni-corrected t-tests). Note that for the Post-stimulation periods, measurements of spontaneous spindle activities were made in the 27 s period after each 3 s optogenetic stimulation pulse train. Spontaneous spindle activity levels were not diminished under 1.0% isoflurane anesthesia as they were comparable to the documented range of spindle density (2–12 events/min) during natural sleep[1-4]. No significant changes in the length and amplitude of spontaneous spindle activities were also found after optogenetic stimulation. **c** Comparisons of spindle density, length, and amplitude across four different anesthesia levels (error bar indicates ± s.e.m.; one-way ANOVA with post-hoc Bonferroni-corrected t-tests). Changes in the anesthesia level within the 0.0–1.5% range did not lead to significant differences in spindle density, length, and amplitude.

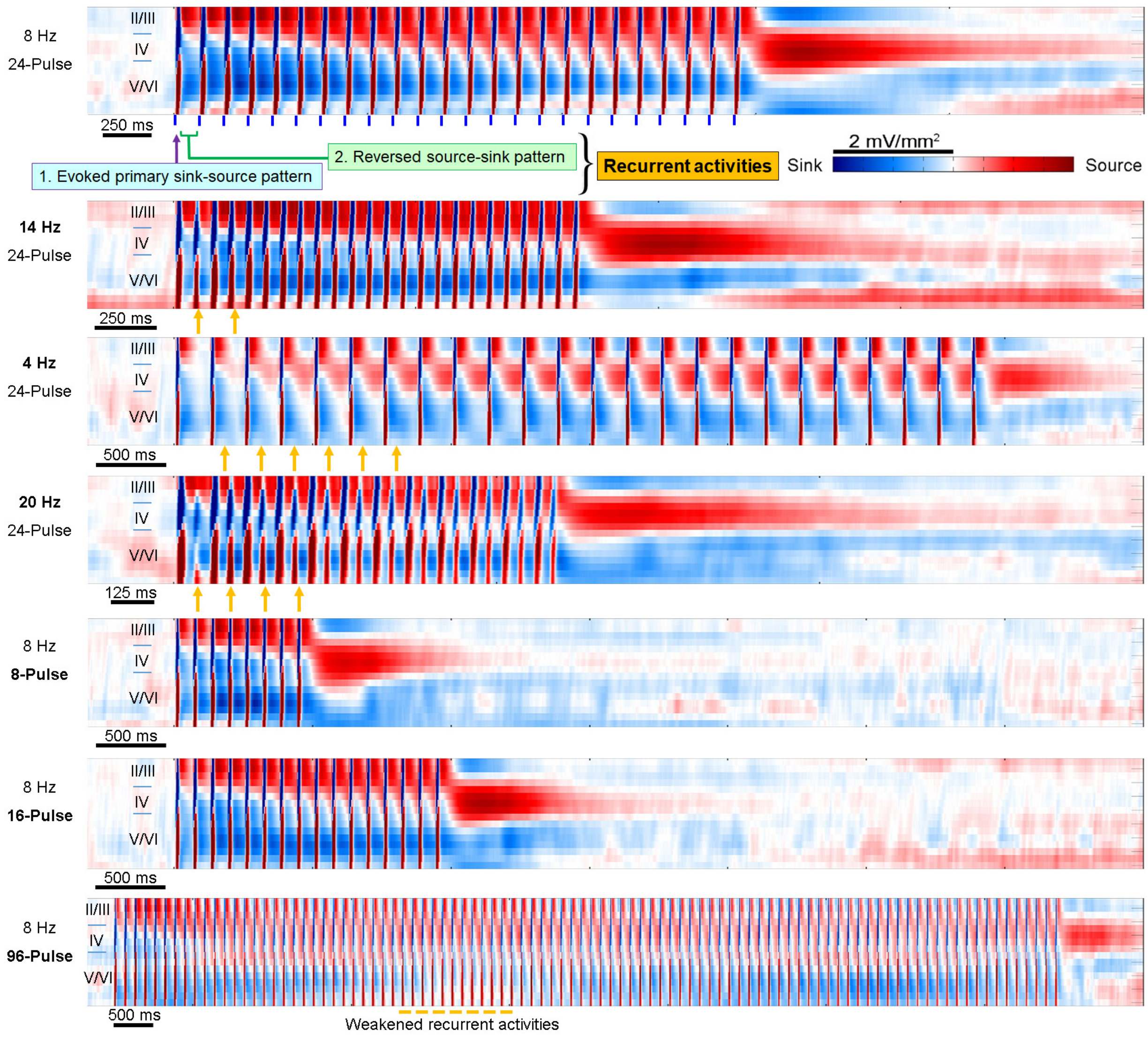

Figure S17. CSD analyses reveal recurrent activities evolving with a spindle-shaped profile. The progressive increase (during the first to third stimulation pulses) and subsequent decrease (after the fourth pulses) in amplitudes of the evoked CSD recurrent activities across all cortical layers (spindle-like evolution pattern) was observed for optogenetic stimulations at 8 and 14 Hz, but not 4 or 20 Hz. These recurrent activities were marked by the reversing of primarily evoked vertically-positioned sink-source patterns (indicated by the purple arrow; sink: layer IV to layer

II/III, source: layer V/VI) into source-sink patterns (indicated by the green brace; sink: layer V/VI, source: layer II/III) at ~50 ms after the onset of each stimulation pulses. Recurrent activities evoked by the 2nd, 4th, and 6th stimulation pulses at 14 and 20 Hz were suppressed or not facilitated (colors not darkened but lightened, areas shrunken, marked by yellow arrows). Reversed source-sink patterns evoked by the 2nd to 7th stimulation pulses at 4 Hz stimulation showed lighter color than those evoked by other frequencies, indicating non-facilitated recurrent activities after the first stimulation pulse. The recurrent activities evoked by the 8 Hz 96-pulse stimulation paradigm showed a weak spindle-shaped evolution pattern, especially after the beginning 3 s of stimulation (marked by the dashed line).

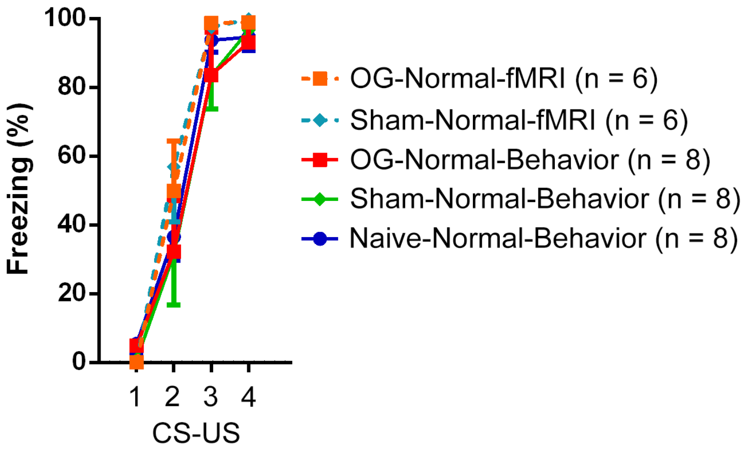

Figure S18. Comparison of visual-somatosensory associative fear memory acquisition for all normal animal groups. No significant differences were found between optogenetic (OG)-fMRI, Sham-fMRI, OG-Behavior, Sham-Behavior, and Naïve-Behavior groups in normal animals and they all reached a ~90% freezing rate level at the end of memory acquisition (error bar indicates ± s.e.m.; mixed-design two-way ANOVA with post-hoc Bonferroni-corrected t-tests). This indicated that all animal groups successfully acquired the visual-somatosensory associative fear memory, and any differences observed post learning were due to the modulatory effects of optogenetically-evoked spindle-like activities on memory consolidation.

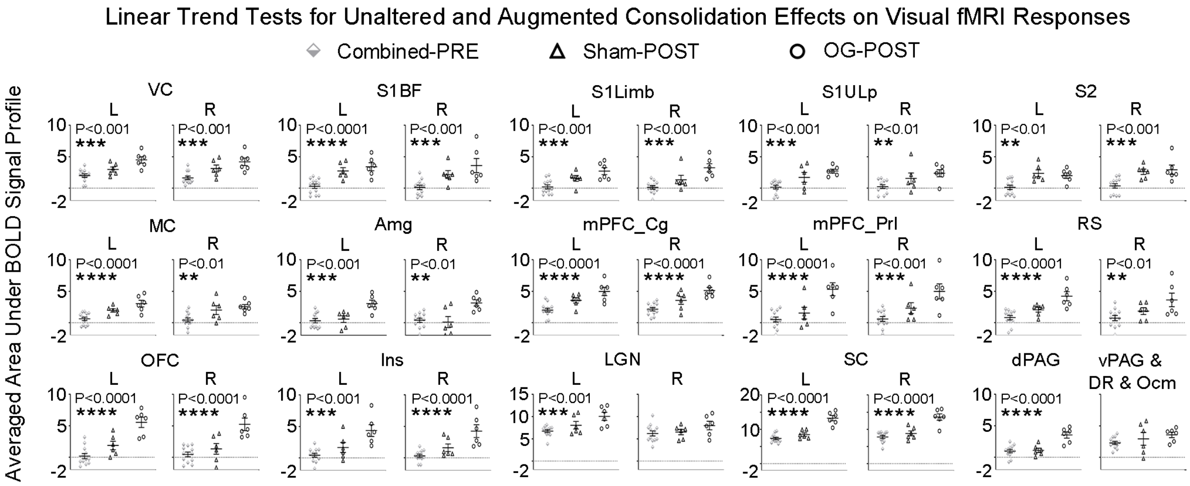

Figure S19. Brain-wide actions of spindle-like activities in normal OG animals can further strengthen visual-somatosensory associative memory representation in similar ways as the learning and consolidation-dependent potentiation effects observed in normal Sham animals. The analyses here supplement those shown in Figures 6 and 7. One-way ANOVA between-condition comparisons with post-hoc tests for linear trend were employed to examine whether the effects of augmented memory consolidation (i.e., differences between OG-POST and Sham-POST, **Figure 7d)** shared similar levels of visual fMRI BOLD response enhancement as those caused by the learning and consolidation-dependent potentiation effects (i.e., differences between Sham-POST and Sham-PRE, **Figure 6d**). Sham-PRE and OG-PRE conditions were treated as the baseline condition (Combined-PRE), and compared with Sham-POST and OG-POST conditions. Results of post-hoc tests for linear trend revealed that the further enhancement of visually-evoked BOLD responses in the OG-POST condition and the increase of visually-evoked BOLD responses in the Sham-POST condition significantly followed a linearly increasing trend in almost all examined regions (error bar indicates ± s.e.m.; n = 6 per group; *, **, *** and **** denote $P < 0.05$, $P < 0.01$, $P < 0.001$ and $P < 0.0001$, respectively). Exact P-values are provided in Source Data.

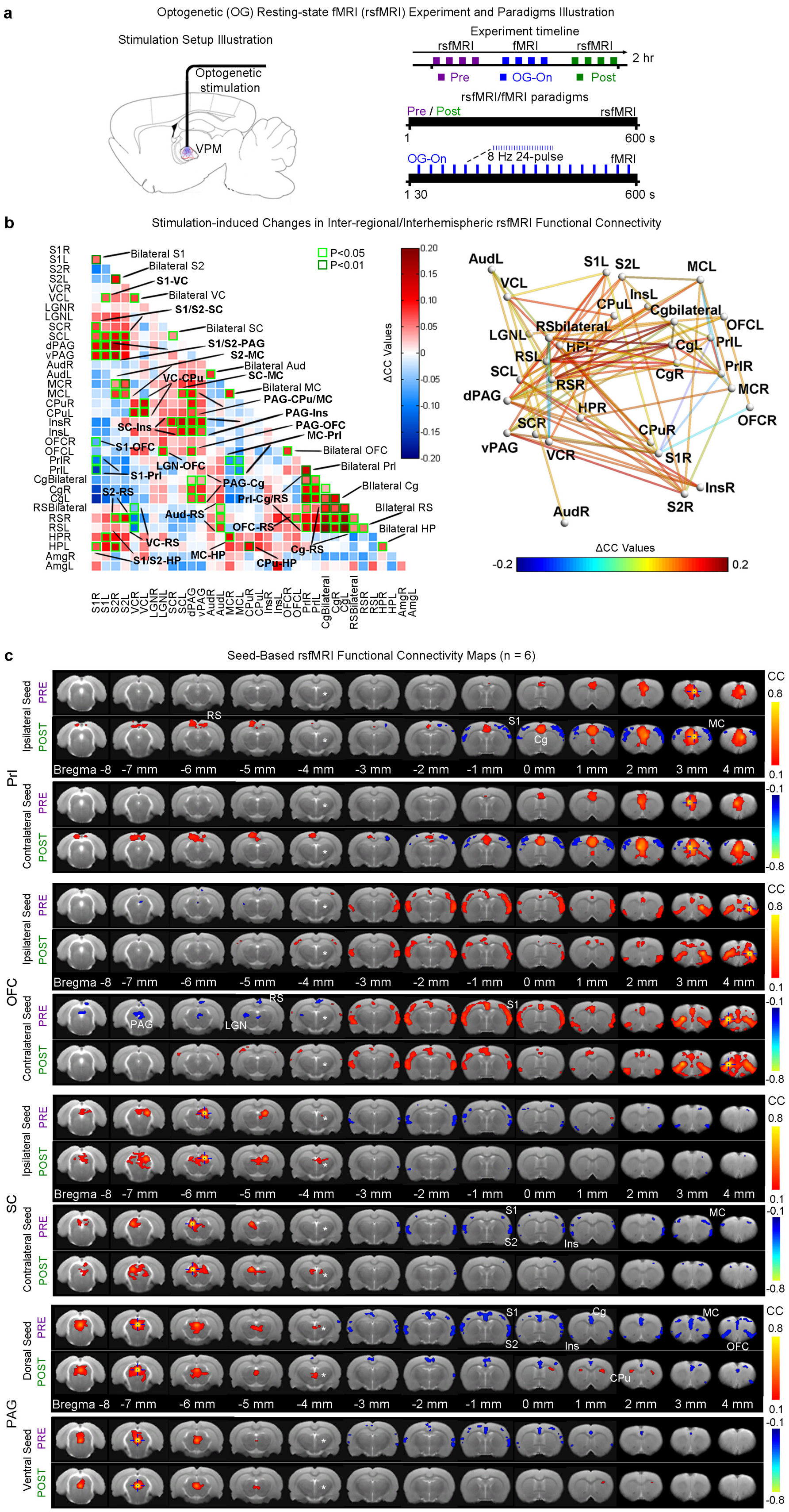
a
Optogenetic (OG) Resting-state fMRI (rsfMRI) Experiment and Paradigms Illustration
Stimulation Setup Illustration
Optogenetic stimulation
VPM
Experiment timeline
rsfMRI
fMRI
rsfMRI
2 hr
Pre
OG-On
Post
rsfMRI/fMRI paradigms
Pre / Post
rsfMRI
1
600 s
OG-On
8 Hz 24-pulse
fMRI
1 30
600 s
b
Stimulation-induced Changes in Inter-regional/Interhemispheric rsfMRI Functional Connectivity
P<0.05
P<0.01
ΔCC Values
ΔCC Values
c
Seed-Based rsfMRI Functional Connectivity Maps (n = 6)
Ipsilateral Seed
Contralateral Seed
Dorsal Seed
Ventral Seed
PRE
POST
Prl
OFC
SC
PAG
Bregma -8
-7 mm
-6 mm
-5 mm
-4 mm
-3 mm
-2 mm
-1 mm
0 mm
1 mm
2 mm
3 mm
4 mm
CC

Figure S20. Optogenetically-evoked spindle-like activities strengthen long-range inter-regional rsfMRI connectivity networks between key sensorimotor and limbic regions. Systems consolidation of memory requires inter-regional integration of representational information (5-7). To provide insight into where spindle-like activities may facilitate large-scale inter-regional functional integration (i.e., cooperation of different brain regions in information processing) to promote such processes (8-10), we probed their effects on brain-wide rsfMRI connectivity that can reflect functional integration during memory (9, 11-13). Specifically, we examined the rsfMRI connectivity of multiple sensorimotor and limbic targets of spindle-like activities and key regions identified in vfMRI memory tests, especially SC, PAG, Ins, Amg, mPFC (includes Prl and Cg), RS and OFC, before (PRE) and after (POST) 8 Hz 24-pulse optogenetic stimulation of VPM. **a** Optogenetic stimulation setup during rsfMRI experiment (left), experimental timeline and a typical rsfMRI scan (right). **b** Stimulation-induced changes in ROI-averaged rsfMRI connectivity correlation coefficient matrix among all examined regions and the network plot for the significantly altered connectivity (n = 6; two-tailed paired t-tests with FDR correction). Significantly enhanced inter-regional connectivity between SC/PAG/Ins/Prl/RS/OFC and other sensorimotor/limbic regions (e.g., S1, S2, MC, Aud, Cg, etc) were found (i.e., increased correlation for inter PAG-Cpu, Prl-Cg and Prl-RS, and decreased anti-correlation for inter SC-S1/S2, SC-MC, SC-Ins, PAG-S1/S2, PAG-MC, PAG-OFC, PAG-Ins, PAG-Cg, S1-RS, Aud-RS, Cg-RS, OFC-RS, OFC-LGN). Other significantly enhanced inter-regional connectivity were mainly linked to sensorimotor cortical regions (S1, S2, VC and MC) and other key sensorimotor or limbic integrative regions (Cpu and HP) (i.e., inter S1-VC, S1/S2-HP, VC-Cpu, MC-HP, and HP-Cpu). We also observed a few significantly decreased inter-regional connectivity between sensorimotor cortices and Prl/OFC/RS (i.e., inter S1-Prl, MC-Prl, S1-OFC and VC-RS). Despite a

trend toward increased Amg-Ins rsfMRI connectivity, we did not find significantly enhanced inter-regional connectivity linked to the Amg. Among the key integrative regions (SC, PAG, Ins, Amg, Prl, RS and OFC) primarily potentiated by spindle-like activities to promote associative memory consolidation and the regions linked to significantly modulated inter-regional rsfMRI connectivity, only SC, PAG and Ins exclusively showed strengthened connectivity. In addition, the strength of interhemispheric rsfMRI connectivity was significantly enhanced in SC, Prl, OFC, S1, S2, VC, Aud, MC, Cg, RS and HP after stimulation. **c** Group-averaged rsfMRI functional connectivity maps of SC, PAG, Prl and OFC seeds before (PRE) and after (POST) 40 min of optogenetically evoking spindle-like activities from VPM thalamocortical excitatory neurons (n = 6; asterisk, stimulation site; blue crosshair, seed and ROI location; two-tailed Pearson's correlation tests, correlation coefficient (CC) value > 0.1 corresponds to uncorrected $P < 0.05$).

Seed-Based Resting-state fMRI (rsfMRI) Functional Connectivity Maps (n = 6)

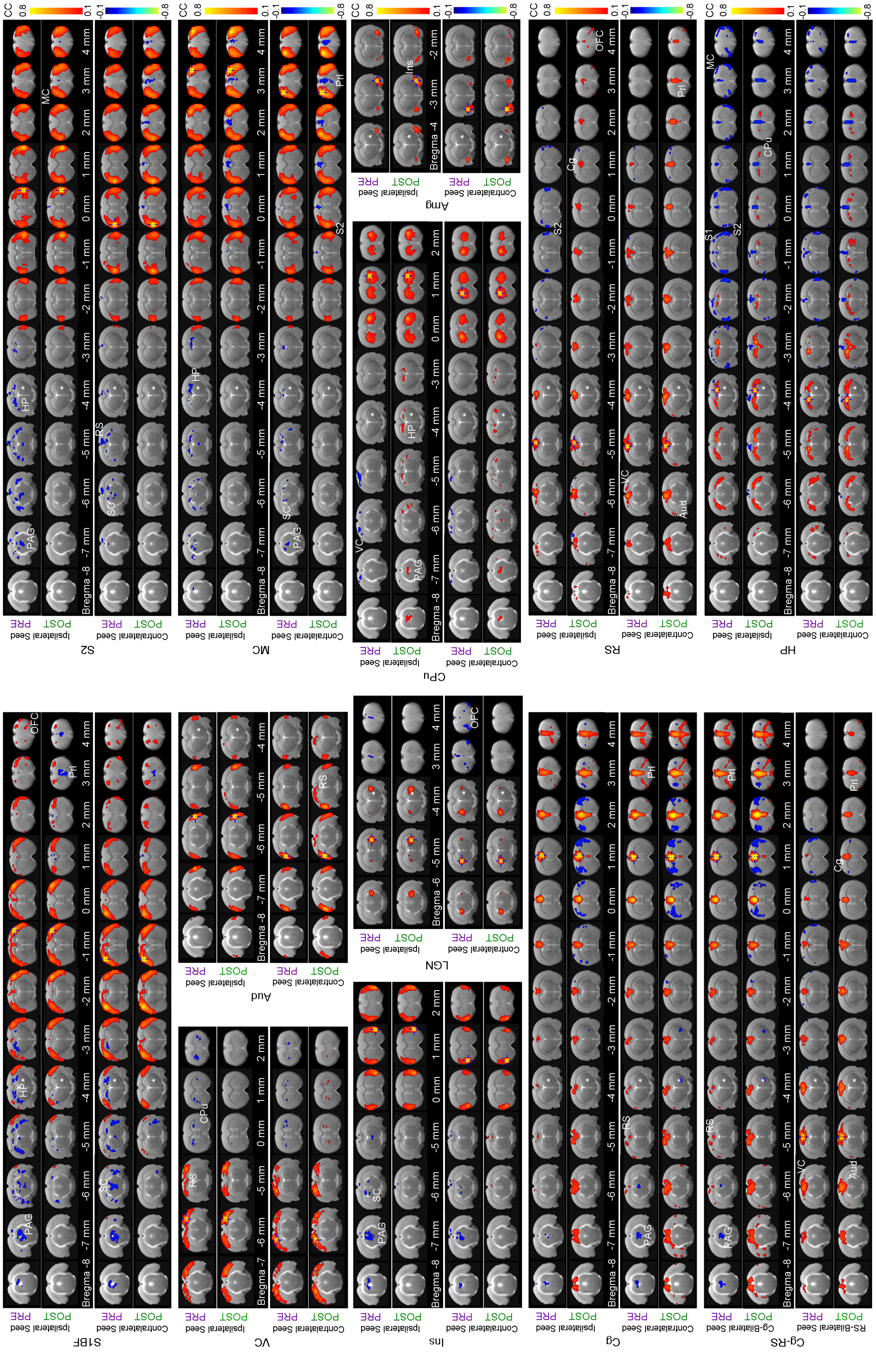

Figure S21. Brain-wide spindle-like activities strengthen brain-wide inter-regional (including interhemispheric) rsfMRI connectivity networks across sensorimotor and limbic regions. Group-averaged rsfMRI functional connectivity maps for ipsilateral and contralateral S1, S2, VC, MC, Aud, LGN, Amg, Ins, CPu, Cg, Prl and HP, and bilateral Cg and RS seeds before (PRE) and after (POST) optogenetically evoking spindle-like activities from VPM thalamocortical excitatory neurons (n = 6; asterisk, stimulation site; blue crosshair, seed and ROI location; two-tailed Pearson's correlation tests, correlation coefficient (CC) value > 0.1 corresponds to uncorrected $P < 0.05$).

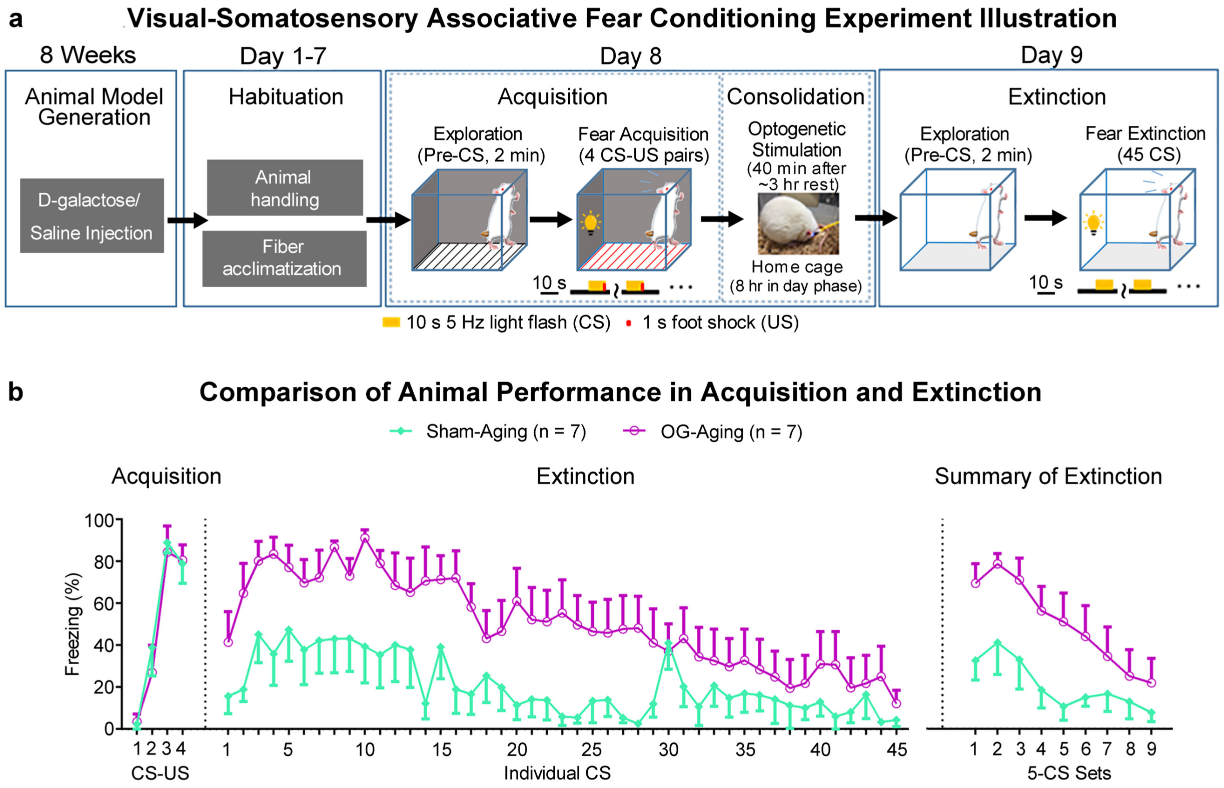

Figure S22. Optogenetically-evoked spindle-like activities alleviate visual-somatosensory associative memory consolidation dysfunction in accelerated aging animals. a Fear conditioning experimental design and timeline (CS: 10s 5Hz light flash; US: foot shock). **b** Comparison of freezing rates in accelerated aging and normal adult animals during acquisition and extinction of visual-somatosensory fear memory (n = 7 per group; error bar indicates ± s.e.m.; two-way ANOVA with: group, $F_{1,12} = 6.143$, $P = 0.029$; set, $F_{8,96} = 9.758$, $P < 0.0001$; group × set interaction, $F_{8,96} = 1.297$, $P = 0.254$; post-hoc Bonferroni-corrected t-tests: no simple effects found in individual time points). Exact P-values are provided in Source Data.

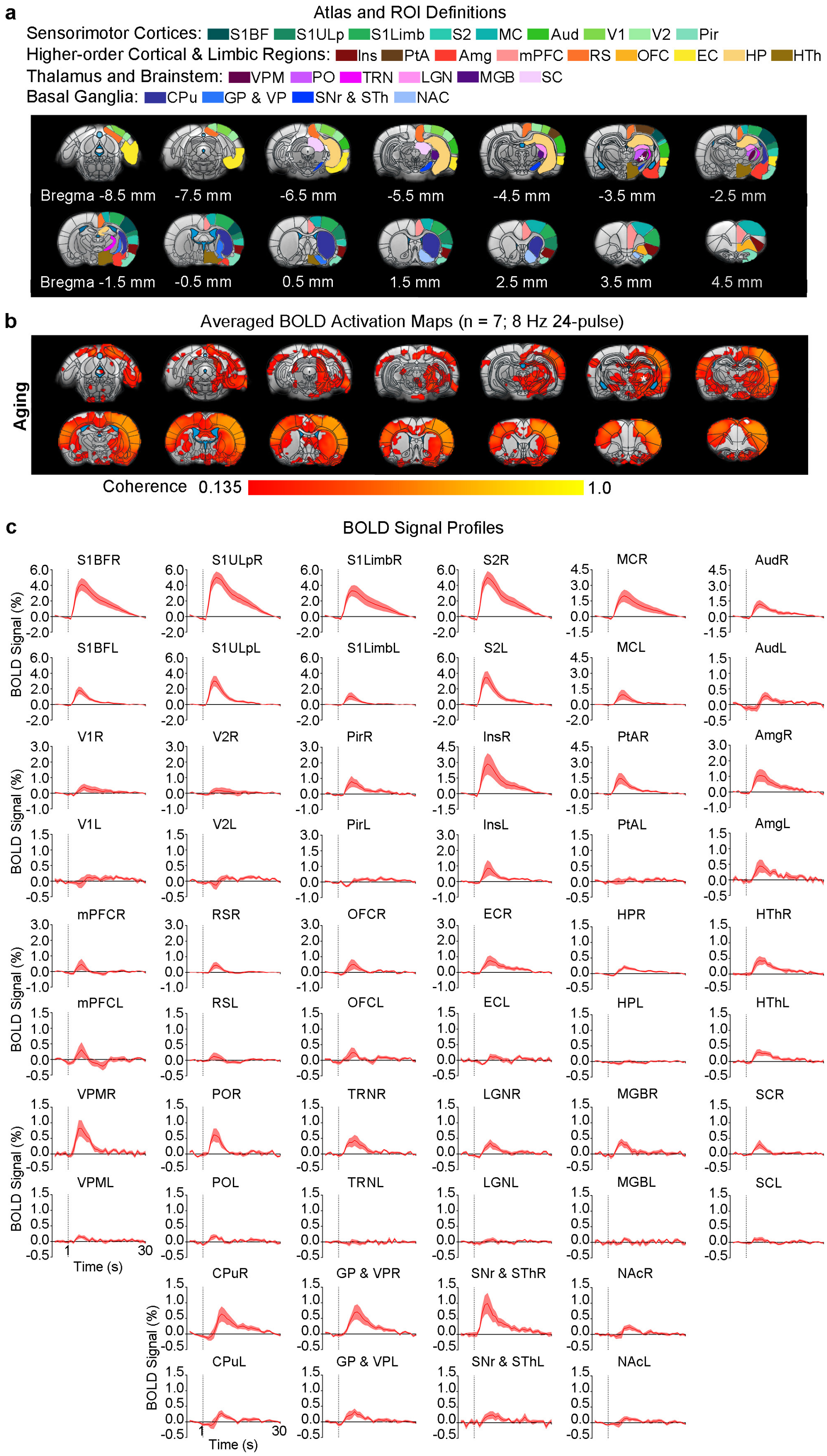
a
Atlas and ROI Definitions
Sensorimotor Cortices: S1BF S1ULp S1Limb S2 MC Aud V1 V2 Pir
Higher-order Cortical & Limbic Regions: Ins PtA Amg mPFC RS OFC EC HP HTh
Thalamus and Brainstem: VPM PO TRN LGN MGB SC
Basal Ganglia: CPu GP & VP SNr & STh NAC
Bregma -8.5 mm
-7.5 mm
-6.5 mm
-5.5 mm
-4.5 mm
-3.5 mm
-2.5 mm
Bregma -1.5 mm
-0.5 mm
0.5 mm
1.5 mm
2.5 mm
3.5 mm
4.5 mm
b
Averaged BOLD Activation Maps (n = 7; 8 Hz 24-pulse)
Aging
Coherence 0.135
1.0
c
BOLD Signal Profiles
S1BFR
S1ULpR
S1LimbR
S2R
MCR
AudR
S1BFL
S1ULpL
S1LimbL
S2L
MCL
AudL
V1R
V2R
PirR
InsR
PtAR
AmgR
V1L
V2L
PirL
InsL
PtAL
AmgL
mPFCR
RSR
OFCR
ECR
HPR
HThR
mPFCL
RSL
OFCL
ECL
HPL
HThL
VPMR
POR
TRNR
LGNR
MGBR
SCR
VPML
POL
TRNL
LGNL
MGBL
SCL
CPuR
GP & VPR
SNr & SThR
NAcR
CPuL
GP & VPL
SNr & SThL
NAcL
BOLD Signal (%)
Time (s)

Figure S23. BOLD activations in accelerated aging animals reveal brain-wide cross-modal targets of somatosensory thalamically-evoked activities upon optogenetic 8 Hz 24-pulse stimulation. a Illustration of atlas-based ROI definitions in the sensorimotor cortices, higher-order cortical and limbic regions, thalamus and brainstem, and basal ganglia (asterisk, stimulation site). **b** Averaged BOLD activation maps for 8 Hz 24-pulse optogenetic stimulation in aging animals (n = 7; asterisk, stimulation site; two-tailed coherence tests, coherence of 0.135 corresponds to $P < 0.001$, followed by two-tailed one-sample group level *t*-tests, TFCE-FWE corrected $P < 0.05$). Robust positive BOLD activations were observed in similar sensorimotor-related cortical, thalamic, brainstem and basal ganglia regions, and non-sensorimotor limbic regions as those in normal animals (**Figures 2, 3, S2 & S5**). **c** BOLD signal profiles extracted from atlas-based ROIs defined in A (error bar indicates ± s.e.m.).

References

1. Kim A, *et al.* (2012) Optogenetically induced sleep spindle rhythms alter sleep architectures in mice. *Proc Natl Acad Sci U S A* 109(50):20673-20678.
2. Latchoumane C-F, Ngo H-V, Born J, & Shin H-S (2017) Thalamic spindles promote memory formation during sleep through triple phase-locking of cortical, thalamic, and hippocampal rhythms. *Neuron* 95(2):424-435.
3. Bandarabadi M, *et al.* (2020) A role for spindles in the onset of rapid eye movement sleep. *Nat Commun* 11(1):5247.
4. Logothetis NK, *et al.* (2012) Hippocampal-cortical interaction during periods of subcortical silence. *Nature* 491(7425):547-553.
5. Dudai Y, Karni A, & Born J (2015) The consolidation and transformation of memory. *Neuron* 88(1):20-32.
6. Klinzing JG, Niethard N, & Born J (2019) Mechanisms of systems memory consolidation during sleep. *Nat Neurosci* 22(10):1598-1610.
7. Diekelmann S & Born J (2010) The memory function of sleep. *Nat Rev Neurosci* 11:114.
8. Helfrich RF, *et al.* (2019) Bidirectional prefrontal-hippocampal dynamics organize information transfer during sleep in humans. *Nat Commun* 10(1):3572.
9. Cowan E, *et al.* (2020) Sleep Spindles Promote the Restructuring of Memory Representations in Ventromedial Prefrontal Cortex through Enhanced Hippocampal-Cortical Functional Connectivity. *J Neurosci* 40(9):1909-1919.
10. Boutin A, *et al.* (2018) Transient synchronization of hippocampo-striato-thalamo-cortical networks during sleep spindle oscillations induces motor memory consolidation. *Neuroimage* 169:419-430.
11. Andrade KC, *et al.* (2011) Sleep spindles and hippocampal functional connectivity in human NREM sleep. *J Neurosci* 31(28):10331-10339.
12. Vahdat S, Fogel S, Benali H, & Doyon J (2017) Network-wide reorganization of procedural memory during NREM sleep revealed by fMRI. *Elife* 6:e24987.
13. Park HJ & Friston K (2013) Structural and functional brain networks: from connections to cognition. *Science* 342(6158):1238411.
