# Supplementary material for: Functional MRI reveals brain-wide actions of thalamically-initiated oscillatory activities on associative memory consolidation
Source: Nat Commun. 2023 Apr 17;14:2195. doi: 10.1038/s41467-023-37682-8 (PMC10110623; doi:10.1038/s41467-023-37682-8)
Supplement: Supplementary file 2 — Reporting Summary [file 41467_2023_37682_MOESM2_ESM.pdf]

# nature portfolio

Corresponding author(s): Ed X. WU

Last updated by author(s): Feb 25, 2023

# Reporting Summary

Nature Portfolio wishes to improve the reproducibility of the work that we publish. This form provides structure for consistency and transparency in reporting. For further information on Nature Portfolio policies, see our Editorial Policies and the Editorial Policy Checklist.

## Statistics

For all statistical analyses, confirm that the following items are present in the figure legend, table legend, main text, or Methods section.

| n/a | Confirmed | |
|---|---|---|
| ☐ | ☒ | The exact sample size ($n$) for each experimental group/condition, given as a discrete number and unit of measurement |
| ☐ | ☒ | A statement on whether measurements were taken from distinct samples or whether the same sample was measured repeatedly |
| ☐ | ☒ | The statistical test(s) used AND whether they are one- or two-sided<br>*Only common tests should be described solely by name; describe more complex techniques in the Methods section.* |
| ☐ | ☒ | A description of all covariates tested |
| ☐ | ☒ | A description of any assumptions or corrections, such as tests of normality and adjustment for multiple comparisons |
| ☐ | ☒ | A full description of the statistical parameters including central tendency (e.g. means) or other basic estimates (e.g. regression coefficient) AND variation (e.g. standard deviation) or associated estimates of uncertainty (e.g. confidence intervals) |
| ☐ | ☒ | For null hypothesis testing, the test statistic (e.g. $F$, $t$, $r$) with confidence intervals, effect sizes, degrees of freedom and $P$ value noted<br>*Give P values as exact values whenever suitable.* |
| ☒ | ☐ | For Bayesian analysis, information on the choice of priors and Markov chain Monte Carlo settings |
| ☒ | ☐ | For hierarchical and complex designs, identification of the appropriate level for tests and full reporting of outcomes |
| ☐ | ☒ | Estimates of effect sizes (e.g. Cohen's $d$, Pearson's $r$), indicating how they were calculated |

*Our web collection on statistics for biologists contains articles on many of the points above.*

## Software and code

Policy information about availability of computer code

| | |
|---|---|
| Data collection | MRI data were collected with ParaVision v5.1 provided by Bruker Biospin GmbH (Ettlingen, Germany);<br>Electrophysiological data were collected with Synapse v86 provided by Tucker Davis Technologies/TDT (Alachua, FL) and OpenEphys v0.4.4.0;<br>Behavior data were collected with Packwin v2.0 and StartFear System provided by Harvard Apparatus (Holliston, Massachusetts, USA). |
| Data analysis | The MATLAB R2018a (MathWorks, USA) was used to process the fMRI, electrophysiology, and behavioral data. The SPM12 fMRI toolbox (Wellcome Department of Imaging Neuroscience, University College London, UK) was used to process the fMRI data. The EEGlab v13.2.1 toolbox (Swartz Center for Computational Neuroscience, University of California San Diego, USA) was used to process the electrophysiological data. |

For manuscripts utilizing custom algorithms or software that are central to the research but not yet described in published literature, software must be made available to editors and reviewers. We strongly encourage code deposition in a community repository (e.g. GitHub). See the Nature Portfolio guidelines for submitting code & software for further information.

# Data

Policy information about availability of data

All manuscripts must include a data availability statement. This statement should provide the following information, where applicable:

- Accession codes, unique identifiers, or web links for publicly available datasets
- A description of any restrictions on data availability
- For clinical datasets or third party data, please ensure that the statement adheres to our policy

The fMRI, electrophysiology and behavioral data generated in in this study are under active use by the reporting laboratory; all the raw data that support the findings of this study are available from the corresponding author upon request. Source data for line graphs and scatter plots of evoked BOLD fMRI signals, electrophysiological traces and behavioral results in the main figures are provided with this paper in the Source Data file.

# Human research participants

Policy information about studies involving human research participants and Sex and Gender in Research.

| | |
|---|---|
| Reporting on sex and gender | N/A |
| Population characteristics | N/A |
| Recruitment | N/A |
| Ethics oversight | N/A |

Note that full information on the approval of the study protocol must also be provided in the manuscript.

# Field-specific reporting

Please select the one below that is the best fit for your research. If you are not sure, read the appropriate sections before making your selection.

- [x] Life sciences
- [ ] Behavioural & social sciences
- [ ] Ecological, evolutionary & environmental sciences

For a reference copy of the document with all sections, see nature.com/documents/nr-reporting-summary-flat.pdf

# Life sciences study design

All studies must disclose on these points even when the disclosure is negative.

| | |
|---|---|
| Sample size | No sample size calculation was performed. Sample sizes were estimated based on previous optogenetic, sensory and resting-state functional MRI (fMRI) and electrophysiology [Leong AT, et al. Long-range projections coordinate distributed brain-wide neural activity with a specific spatiotemporal profile. Proc Natl Acad Sci U S A 113(51):E8306-E8315, (2016) and Chan RW, et al. Low-frequency hippocampal–cortical activity drives brain-wide resting-state functional MRI connectivity. Proc Natl Acad Sci U S A 114(33):E6972-E6981, (2017) ] and behavioral studies [Latchoumane CV et al. Thalamic Spindles Promote Memory Formation during Sleep through Triple Phase-Locking of Cortical, Thalamic, and Hippocampal Rhythms. Neuron 95(2):424-435, (2017)] |
| Data exclusions | For all fMRI data, scans suffering from motion artifacts > 0.05 mm voxel shifts were discarded;<br><br>For behavioral only experiments, animals that showed over-generalized fear (freezing rate of > 40% during exploration, 2 Naïve-Normal, 2 OG-Normal and 2 Sham-Normal animals) and failed during the memory acquisition phase (freezing rate of < 50% during the presentation of the final CS-US pair, one Sham-Normal, one Sham-Aging and 2 OG-Normal animals) were excluded from further analysis; One OG-Normal animals that did not stay at quiescent or sleep states (i.e., with eyes closed and curled-up body posture) during stimulation was excluded from further experiments.<br><br>For animals that underwent both behavioral and visual fMRI experiments, animals that met the same exclusion criteria as in the behavioral only experiments or displayed weak baseline visual fMRI activations were excluded from further analysis. One animal that suffered from weak visual fMRI responses was excluded. |
| Replication | Experimental findings (i.e., fMRI, electrophysiology and behavioral experiments) were replicated across multiple animals (indicated by 'n' for each experimental findings presented in the figures). Furthermore, for each animal, fMRI experiment was repeated across numerous sessions (two for each stimulation paradigm in the optogenetic fMRI experiments, eight for each condition in the visual fMRI experiments, four for each condition in the resting-state fMRI experiments). We use electrophysiological recordings and behavioral experiments to cross-validate our fMRI findings. |
| Randomization | The animals were allocated randomly into different experimental groups. Different stimulation paradigms were interleaved within animal and sequence of different stimulation paradigms were randomized across different animals. |

| | |
|---|---|
| Blinding | For fear conditioning experiments (including those with visual fMRI experiments), blinding was not done, as we needed to counterbalance the time points when the animals underwent the tests, while the freezing values were calculated using a high sensitivity Weight Transducer System (StartFear System, Harvard Apparatus, Holliston, Massachusetts, USA) to avoid experimenter bias. For optogenetic fMRI, resting-state fMRI and electrophysiology experiments, blinding is not relevant as experimenters need to test all stimulation paradigms/conditions for all animals in each group. |

# Reporting for specific materials, systems and methods

We require information from authors about some types of materials, experimental systems and methods used in many studies. Here, indicate whether each material, system or method listed is relevant to your study. If you are not sure if a list item applies to your research, read the appropriate section before selecting a response.

## Materials & experimental systems

| n/a | Involved in the study | |
|---|---|---|
| ☐ | ☒ | Antibodies |
| ☒ | ☐ | Eukaryotic cell lines |
| ☒ | ☐ | Palaeontology and archaeology |
| ☐ | ☒ | Animals and other organisms |
| ☒ | ☐ | Clinical data |
| ☒ | ☐ | Dual use research of concern |

## Methods

| n/a | Involved in the study | |
|---|---|---|
| ☒ | ☐ | ChIP-seq |
| ☒ | ☐ | Flow cytometry |
| ☐ | ☒ | MRI-based neuroimaging |

## Antibodies

| | |
|---|---|
| Antibodies used | Primary antibodies against rabbit polyclonal to CaMKIIα (1:400; Abcam) and secondary antibodies Alexa Fluor 647 conjugate goat anti-rabbit IgG and Alexa Fluor 488 conjugate goat anti-guinea pig IgG (both 1:500; Molecular Probe) for immunohistochemistry preparation of brain slices. |
| Validation | Confocal microscopy to examine the florescence in neurons. |

## Animals and other research organisms

Policy information about studies involving animals; ARRIVE guidelines recommended for reporting animal research, and Sex and Gender in Research

| | |
|---|---|
| Laboratory animals | Adult male Sprague Dawley rats at 6-18 weeks of age were used in this study. |
| Wild animals | No wild animals were used in the study. |
| Reporting on sex | The research findings apply to only male rats. We did not consider sex in our experiments and did not perform sex-based analysis in this study as no consensus has been made in the field on the sex differences of spindle activities and their effects on memory consolidation. |
| Field-collected samples | No field collected samples were used in the study. |
| Ethics oversight | All animal experiments were approved by the University of Hong Kong's Committee on the Use of Live Animals in Teaching and Research (CULATR). |

Note that full information on the approval of the study protocol must also be provided in the manuscript.

## Magnetic resonance imaging

### Experimental design

| | |
|---|---|
| Design type | Task-based and resting-state fMRI. |
| Design specifications | For optogenetic fMRI experiments, stimulation pulse trains with varied frequencies (4, 8, 14, and 20 Hz for 24-pulse) or lengths (8-, 16-, 24-, and 96-pulse for 8 Hz) were used (light intensity = 8 mW: 40 mW/mm2; 10 ms pulse width). We covered slow and fast spindle frequencies (8 and 14 Hz, respectively), and frequencies below or above (4 and 20 Hz, respectively) the typical range of spindle frequency. Lengths for 8 Hz stimulations were chosen to be within or above the typical range of spindle length (i.e., 1, 2, 3, or > 3 s). Two 600 s sessions were acquired for each stimulation paradigm. For each session, the same stimulation pulse train was repeated every 30 s. Different stimulation paradigms were interleaved within animal and sequence of different stimulation paradigms were randomized across different animals.<br><br>The visual fMRI (vfMRI) paradigm used consisted of six blocks of 10 s visual stimulation and 30 s rest with each session lasting 270 s. Sixteen sessions (eight for PRE, eight for POST) were acquired in total for each animal. |

| | |
|---|---|
| | For resting-state fMRI (rsfMRI), a total of eight sessions were acquired for each animal, four before (PRE) and four after (POST) four sessions of optogenetic stimulation. For each session of optogenetic stimulation (OG-On), 8 Hz 24-pulse stimulation paradigm was employed. |
| Behavioral performance measures | No behavioral measures were performed during fMRI scans as the animals were anesthetized. |

## Acquisition

| | |
|---|---|
| Imaging type(s) | Functional and anatomical |
| Field strength | 7 Tesla |
| Sequence & imaging parameters | RARE T2-weighted (T2W) images were first acquired for accurate positioning and reference with field of view (FOV) = 32 × 32 mm2, matrix = 256 × 256, RARE factor = 8, echo time (TE) = 36 ms, repetition time (TR) = 4200 ms. Sixteen contiguous 1.0-mm slices were positioned in the transverse orientation according to the rat brain atlas to cover the majority of the brain. All fMRI, vfMRI, and rsfMRI data were obtained at the same geometry as the anatomical reference T2W images, using a single-shot gradient-echo echo-planar imaging (GE-EPI) sequence with FOV = 32 × 32 mm2, matrix = 64 × 64, flip angle = 56° (optogenetic fMRI and vfMRI) or 50° (rsfMRI), TE = 20 ms, TR = 1000 ms (optogenetic fMRI and vfMRI) or 750ms (rsfMRI). |
| Area of acquisition | Whole brain |

Diffusion MRI ☐ Used ☒ Not used

## Preprocessing

| | |
|---|---|
| Preprocessing software | Matlab R2018a with SPM12 toolbox; all EPI images were first corrected for slice timing differences and realigned (scans suffering from motion artifacts > 0.125 mm voxel shifts were discarded), the EPI images were registered to their T2W images before the T2W images from each animal were coregistered to a anatomical template brain. The EPI were spatially smoothed at full-width at half maximum (FWHM) of 0.5 x 0.5 mm. |
| Normalization | The EPI images from the same animal were registered to their T2W images before the T2W images from each animal were coregistered to a anatomical brain MRI template using affine transformation with a normalized mutual information matrix. |
| Normalization template | The anatomical reference image is a T2W image acquired and generated from a age-matched normal rat brain that best corresponds to the geometrical orientation utilized by the rat brain atlas. |
| Noise and artifact removal | fMRI scans suffering from motion artifacts (> 0.05 mm voxel shifts detected by realignment) and sudden physiological changes (i.e., abrupt changes in respiration pattern, heart rate, and oxygen saturation level) were discarded. Linear temporal detrending was applied for correction of baseline drift caused by physiological noises and system instability were applied. A high pass filtering was further employed to remove any signal drifts or confounds slower than 128 s/cycle. Band-pass filtering (0.005 - 0.1 Hz) was applied for rsfMRI data. |
| Volume censoring | No fMRI volume was censored during preprocessing. |

## Statistical modeling & inference

| | |
|---|---|
| Model type and settings | Optogenatic fMRI:  voxel-wise coherence analysis with reference to the stimulation presentation rate (once every 30 s) with TFCE-FWE or Bonferroni corrections to detect brain-wide activations of each stimulation paradigm; between-paradigm comparisons were done by performing voxel-wise two sample t-tests with GRF and TFCE-FWE multiple comparison corrections to reveal the significant influence of stimulation frequency and length on brain-wide cross-modal activations.<br><br>Visual fMRI: first level analysis using general linear model with reference to the stimulation design, followed by ROI-based activation quantification and comparisons between conditions (i.e., PRE vs. POST) using one-tailed paired t-tests to identify the learning-dependent (or learning & optogenetic stimulation-induced) changes in fMRI activations to visual stimuli in each animal group. Between-group comparisons were done by performing two-way ANOVA with FDR post hoc tests to reveal regions that showed stronger responses to visual stimuli in the OG group than in the Sham group. One-way ANOVA with post-hoc tests for linear trend was employed to examine whether the further enhancement of vfMRI responses in the OG group compared to Sham group shared similar levels of response enhancement as those observed in the Sham group caused by learning and consolidation-dependent potentiation effects.<br><br>rsfMRI connectivity: Seed-based voxel-wise Pearson correlation analysis; one sample t-tests ($P < 0.05$) within each condition (i.e., “Pre” and “Post”) followed by two-tailed paired t-tests with FDR corrections ($P < 0.05$) for between condition comparisons (i.e., “Pre” vs. “Post”). |
| Effect(s) tested | Optogenatic fMRI: Activations during each optogenetic stimulation paradigm; differences in activation maps between 8 Hz 24-pulse stimulation and other stimulation frequencies and lengths using two sample t-tests.<br><br>Visual fMRI: Activations during visual stimulation; increases of ROI-quantified activation levels due to fear conditioning or fear conditioning + optogenetic stimulation within each group (i.e., OG or Sham); differences in ROI-quantified activation levels between groups (i.e., OG vs. Sham) and conditions (i.e., PRE vs. POST) using two-way ANOVA; effects of increasing spindle activities on fMRI activations using one-way ANOVA (Combined-PRE, Sham-POST, and OG-POST). |

rsfMRI connectivity: the interhemispheric/inter-regional connectivity changes from Pre stimulation to Post stimulation conditions

Specify type of analysis: ☐ Whole brain ☐ ROI-based ☒ Both

Anatomical location(s): Based on Paxinos atlas.

| | |
|---|---|
| Statistic type for inference (See Eklund et al. 2016) | Optogenatic fMRI: Voxel-wise for coherence analyses and Student's t-tests.<br><br>Visual fMRI: Voxel-wise Student's t-test calculation, followed by ROI-based comparisons using Student's t-test for within-group between-condition comparisons and ANOVA for between-group between-condition comparisons.<br><br>rsfMRI: Seed-based voxel-wise calculation followed by ROI based comparison using Student's t-test. |
| Correction | Optogenatic fMRI: TFCE-FWE correction with $P < 0.05$, Bonferroni correction with $P < 0.05$; GRF correction with voxel level $P < 0.05$ and cluster level $P < 0.001$.<br><br>Visual fMRI: Student's t-test with a significance threshold of uncorrected $p < 0.001$, FDR corrections for multiple comparisons.<br><br>rsfMRI connectivity: FDR corrections for multiple comparisons. |

## Models & analysis

| n/a | Involved in the study |
|---|---|
| ☐ | ☒ Functional and/or effective connectivity |
| ☒ | ☐ Graph analysis |
| ☒ | ☐ Multivariate modeling or predictive analysis |

| | |
|---|---|
| Functional and/or effective connectivity | Seed-based Pearson correlation analysis were performed, followed by statistical comparison of ROI-based averaged of correlation coefficient values: one sample t-tests ($P < 0.05$) within each condition (i.e., "Pre" and "Post"), and two-tailed paired t-tests with FDR corrections ($P < 0.05$) were applied for between condition comparisons. |
